# Physiology of intracellular calcium buffering

**DOI:** 10.1152/physrev.00042.2022

**Published:** 2023-06-16

**Authors:** David Eisner, Erwin Neher, Holger Taschenberger, Godfrey Smith

**Affiliations:** ^1^Division of Cardiovascular Sciences, University of Manchester, Manchester, United Kingdom; ^2^Membrane Biophysics Laboratory, Max Planck Institute for Multidisciplinary Sciences, Göttingen, Germany; ^3^Cluster of Excellence “Multiscale Bioimaging: from Molecular Machines to Networks of Excitable Cells” (MBExC), University of Göttingen, Göttingen, Germany; ^4^Department of Molecular Neurobiology, Max Planck Institute for Multidisciplinary Sciences, Göttingen, Germany; ^5^School of Cardiovascular and Metabolic Health, College of Medical, Veterinary, and Life Sciences, University of Glasgow, Glasgow, United Kingdom

**Keywords:** buffer, calcium

## Abstract

Calcium signaling underlies much of physiology. Almost all the Ca^2+^ in the cytoplasm is bound to buffers, with typically only ∼1% being freely ionized at resting levels in most cells. Physiological Ca^2+^ buffers include small molecules and proteins, and experimentally Ca^2+^ indicators will also buffer calcium. The chemistry of interactions between Ca^2+^ and buffers determines the extent and speed of Ca^2+^ binding. The physiological effects of Ca^2+^ buffers are determined by the kinetics with which they bind Ca^2+^ and their mobility within the cell. The degree of buffering depends on factors such as the affinity for Ca^2+^, the Ca^2+^ concentration, and whether Ca^2+^ ions bind cooperatively. Buffering affects both the amplitude and time course of cytoplasmic Ca^2+^ signals as well as changes of Ca^2+^ concentration in organelles. It can also facilitate Ca^2+^ diffusion inside the cell. Ca^2+^ buffering affects synaptic transmission, muscle contraction, Ca^2+^ transport across epithelia, and the killing of bacteria. Saturation of buffers leads to synaptic facilitation and tetanic contraction in skeletal muscle and may play a role in inotropy in the heart. This review focuses on the link between buffer chemistry and function and how Ca^2+^ buffering affects normal physiology and the consequences of changes in disease. As well as summarizing what is known, we point out the many areas where further work is required.

## Graphical abstract

**Figure:**
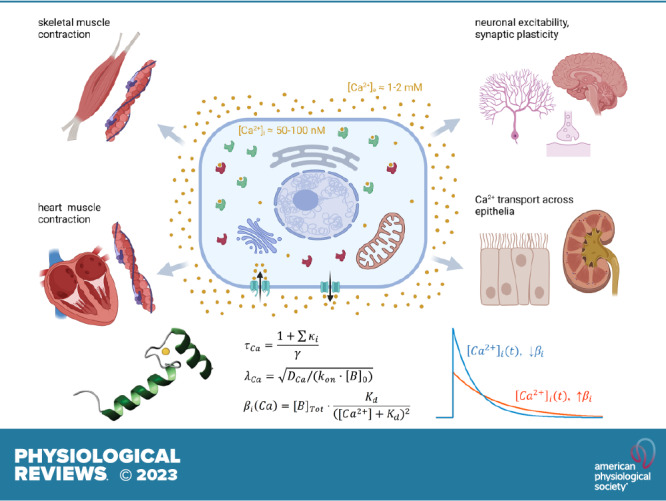

CLINICAL HIGHLIGHTS

- Intracellular calcium controls the function of all organs in the body.
- Most of the intracellular calcium is not free; rather, it is bound to calcium buffers. This affects the amplitude and time course of intracellular calcium transients.
- Disordered intracellular calcium regulation is linked to many pathological conditions. Abnormal calcium buffering, as opposed to changes in transmembrane fluxes, has been suggested as a primary pathological mechanism.
- For example, in common conditions such as atrial fibrillation and heart failure, altered intracellular calcium buffering is thought to contribute to proarrhythmic behavior.
- In rarer genetic conditions such as hypertrophic cardiomyopathy, altered calcium buffering caused by mutations in contractile proteins is thought to promote abnormal contraction time course and increased incidence of arrhythmias. Initial studies suggest that drugs targeting the abnormal calcium buffering may improve contraction kinetics and reduce arrhythmias.
- In neurons, calcium-binding proteins contribute to the regulation of electrical excitability and short-term synaptic plasticity.
- Altered neuronal expression of calcium-binding proteins has been reported in the context of neurological diseases including dementia, epilepsy, and ataxia, but whether this is the primary cause or a consequence of abnormal neural function is less established.
- This review introduces the readership of *Physiological Reviews* to the basic chemistry of calcium buffers and discusses how this relates to their function in both health and disease. Although modulation of intracellular calcium buffering may represent a promising novel therapeutic route, challenges such as the complex interdependence of calcium signaling and the need for tissue-specific interventions need to be addressed in future research.

## 1. INTRODUCTION

The importance of Ca^2+^ ions in regulating cell and tissue function is well established. Life begins with an increase of intracellular calcium concentration ([Ca^2+^]_i_) at the moment of fertilization (1). Cell death (apoptosis) is also accompanied by a Ca^2+^ signal (2). In between, the function of virtually every cell and tissue is controlled or influenced by changes of [Ca^2+^]_i_, and many important diseases involve disorders of Ca^2+^ signaling (for reviews see, e.g., Refs. 3–10). This Ca^2+^-dependent regulation is mediated by Ca^2+^ ions binding to proteins and thereby changing their structure and function. However, a consequence of this binding is that changes in cytoplasmic free Ca^2+^ are strongly constrained or “buffered,” with typically only ∼1% being free and ionized at resting [Ca^2+^]_i_. The chemistry of ionic buffering was first described with respect to the hydrogen ion (H^+^) in the early twentieth century and quantified initially by Koppel and Spiro (11) (see also Ref. 12) and subsequently by van Slyke (13). One of the first physiological indications of Ca^2+^ buffering, noted >70 years ago, was that intracellular Ca^2+^ is relatively immobile (14). Subsequent work on the squid axon led to the concept that most cytoplasmic Ca^2+^ is bound to Ca^2+^-binding molecules rather than being freely ionized (15), and similar conclusions have been reached for other tissues.

This buffering function of Ca^2+^-binding molecules means that not only the amplitude but also the time course of changes of [Ca^2+^]_i_ may depend as much on the properties of the Ca^2+^ buffers as on the magnitude of the underlying Ca^2+^ fluxes. As described in this review, Ca^2+^ buffers are essential regulators for such important and diverse functions as muscle contraction, neuronal excitability and synaptic facilitation, epithelial transport, and killing of bacteria. The effects of buffers are not restricted to the cytoplasm but also extend to endoplasmic reticulum (ER), mitochondrial, and nuclear Ca^2+^.

Much is now known about the molecules that buffer Ca^2+^ and how their chemical properties determine this buffering. For many tissues, however, the quantitative contribution of the various potential buffers still remains to be established. Buffers differ in the affinity and speed with which they bind and release Ca^2+^ ions, and this binding can also be affected by other ions such as Mg^2+^ and protons. It is therefore important to understand how functional properties of Ca^2+^ buffers relate to their structure and chemistry. Some Ca^2+^ buffers are mobile, and binding to these can accelerate the rate at which Ca^2+^ diffuses within the cytoplasm. Another important and underappreciated issue concerns the factors that determine the strength or power of Ca^2+^ buffering. For example, how does buffering depend on the Ca^2+^ concentration and the affinity of the buffer for Ca^2+^, and how is it different for buffers that bind more than one Ca^2+^ in a cooperative manner?

As well as being physiologically important, experimentally added Ca^2+^ buffers have been used to control Ca^2+^ concentrations and probe cellular mechanisms of Ca^2+^ handling. Ca^2+^-sensitive fluorescent indicators used to measure [Ca^2+^]_i_ are Ca^2+^ buffers and accordingly alter Ca^2+^ signaling.

Despite its importance, buffering is much less well understood than are many other aspects of Ca^2+^ signaling. In this review, we first consider the chemistry of Ca^2+^ buffering and the factors that determine the extent and speed of buffering. We emphasize the importance of the quantitative properties of buffering, deriving these from physicochemical principles and simplifying assumptions to describe general relationships. We show how the physiology of cells as diverse as epithelial cells, neurons, and muscle and immune cells is shaped by the chemistry of Ca^2+^ buffering and how a given buffer can be used to control the function of diverse tissues. Finally, there is much that is still not understood about buffering, and we highlight new directions for research.

## 2. CHEMISTRY OF Ca^2+^ BUFFERING

**FIGURE 1.**
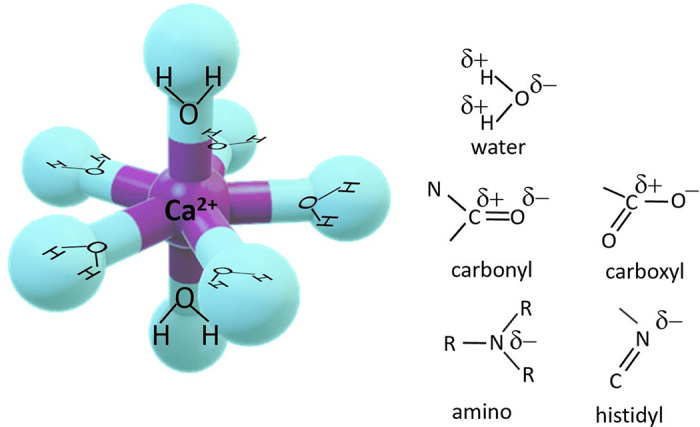
Pentagonal bipyramid arrangement of water around the Ca^2+^ ion in aqueous solution. In the chelation process, each water molecule is replaced by an organic residue that has an electronegative pole to support a dative covalent bond with Ca^2+^. The common groups that participate in chelation are shown with the electronegative poles designated.

**FIGURE 2.**
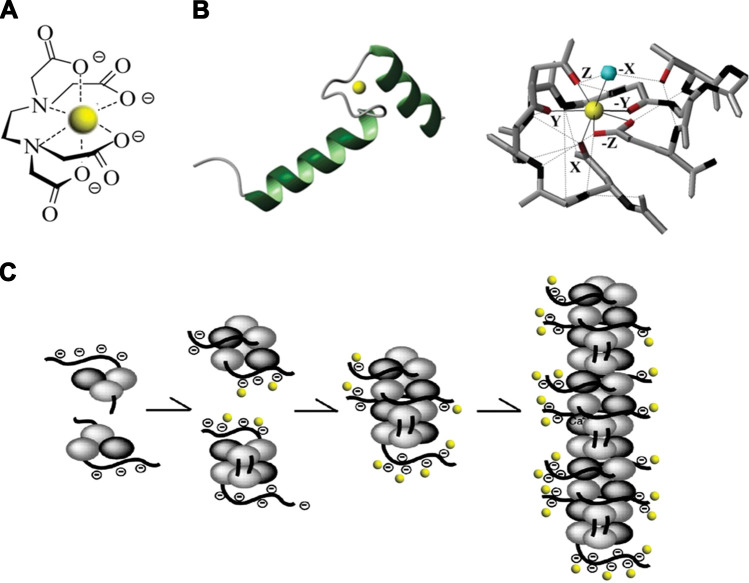
Examples of chelate structures. *A*: the small molecule EDTA adapted from Ref. 19, with permission from *Fibres and Textiles in Eastern Europe. B*: the EF-hand domain in terms of the helix-loop-helix structure (*left*) and the detail of the chelation site for the canonical form (*right*). Adapted from Ref. 17, with permission from *Biochemical Journal*. *C*: the proposed reaction scheme for the polymerization of calsequestrin (CSQ). Ca^2+^ ions are denoted as yellow balls in *A–C*; adapted from Ref. 20, with permission from *Journal of Biological Chemistry*.

In aqueous solution, Ca^2+^ ions electrostatically form a solvation sphere with 6–10 water molecules (16). Chelation is the process of replacing two or more of these water molecules with dative covalent bonds between the Ca^2+^ ion and a ligand molecule and is the mechanism through which Ca^2+^ ions in solution bind to both small synthetic molecules and various Ca^2+^-binding proteins. In biological environments, the energetically optimum coordination of water around Ca^2+^ is 7, with 5 points arranged almost in a planar pentagon and 2 on an orthogonal plane creating a pentagonal bipyramid (17) (FIGURE 1). Most synthetic and biological chelators replace 6 or 7 of the water molecules with a chelation structure. Molecules interact directly with Ca^2+^ via electronegative points on the ligand molecule, typically negatively charged oxygen on a carboxylate group, the electronegative carbonyl group, or the lone pair of electrons of amino or histidyl groups (18) (FIGURE 2). Synthetic or natural Ca^2+^-binding molecules present an array of these electronegative residues to form a clawlike structure around each Ca^2+^ ion. The dative covalent bonds can form on each of the *X*, *Y*, and *Z* planes around the ion, thereby excluding most of the water molecules in the solvation sphere (17, 18).

### 2.1. Kinetics and Affinity


reaction 1 $$\mathrm{Ca}+B\overset{k_{\mathrm{on}}}{\underset{k_{\mathrm{off}}}{⇌}}\mathrm{CaB}$$

The simplest Ca^2+^ binding scheme is described in *reaction 1*. More complicated reactions, involving cooperative binding to multiple sites or competition with other ions are considered below. Here, Ca^2+^ binds to the buffer (B) with a rate constant k_on_ (in M^−1^·s^−1^) and dissociates with rate constant k_off_ (in s^−1^). As described in sect. 3, for a total buffer concentration, [B]_Tot_, the equilibrium concentration of Ca^2+^ bound to buffer, [CaB], will be given by

(*1*) $$[\mathrm{CaB}]={\left[B\right]}_{\mathrm{Tot}}\cdot \frac{{\left[Ca^{2+}\right]}_{i}}{K_{d}+{\left[Ca^{2+}\right]}_{i}}$$

The equilibrium dissociation constant is given by *K*_d_ = k_off_/k_on_. In chemistry, it is more customary to describe the association reaction of *reaction 1* with an association constant (*K*_a_ = k_on_/k_off_). However, *K*_a_ has units of M^−1^, and its inverse, the *K*_d_, is easier to appreciate as it is equivalent to the concentration of free Ca^2+^ at which 50% of the buffer has Ca^2+^ bound. Interestingly, almost regardless of the structure, the rate of Ca^2+^ binding to a buffer is generally rapid and close to the diffusion limit of 10^3^ to 10^4^ µM^−1^·s^−1^ (10^9^ to 10^10^ M^−1^·s^−1^) in aqueous media (21). Such rates are technically difficult to measure and involve measurements on isolated proteins/molecules under chemical conditions that are far from physiological. Dissociation rate constants and equilibrium constants are easier to measure accurately, and therefore very fast association rate constants are often calculated as the ratios of k_off_ over *K*_d_. Values of *K*_d_s vary greatly across a range of chelation structures, with low-affinity buffers resulting from structures with high k_off_ values.

### 2.2. Competition with Mg^2+^ and Protons

A mechanism that is frequently involved in lowering the affinity of a buffer is a decreased effective k_on_ of the chelation/association reaction due to sequestering of the free buffer by other competing cations. As discussed below and illustrated in *reactions 2A* and *2B*, if Mg^2+^ or protons are bound to a buffer, these ions must dissociate before Ca^2+^ can bind. This additional step slows down the overall rate constant for the Ca^2+^-binding reaction and therefore decreases the “apparent” affinity of the buffer under these conditions. For some buffers (for example ATP), even in the presence of Mg^2+^ or H^+^, Ca^2+^ binding is still rapid, whereas for others the slowing is profound (TABLE 1).

reaction 2A $${\text{Ca}}^{\text{2}+}+\text{BMg}\overset{{\text{k}}_{\text{on},\text{Mg}}}{\underset{{\text{k}}_{\text{off},\text{Mg}}}{⇋}}{\text{Mg}}^{\text{2}+}+{\text{Ca}}^{\text{2}+}+\text{B}\;\overset{{\text{k}}_{\text{on},\mathrm{Ca}}}{\underset{{\text{k}}_{\text{off},\mathrm{Ca}}}{⇌}}\text{CaB}+{\text{Mg}}^{\text{2}+}$$

reaction 2B $${\text{Ca}}^{\text{2}+}+\text{BH}\overset{{\text{k}}_{\text{on},\text{H}}}{\underset{{\text{k}}_{\text{off},\text{H}}}{⇋}}{\text{H}}^{+}+{\text{Ca}}^{\text{2}+}+\text{B}\;\overset{{\text{k}}_{\text{on},\text{Ca}}}{\underset{{\text{k}}_{\text{off},\text{Ca}}}{⇌}}\text{CaB}+{\text{H}}^{+}$$

**Table 1.** Examples of K_d_ and rate constants (both measured and apparent), for buffers which have a significant affinity for Mg^2+^ and or H^+^ in addition to Ca^2+^

Buffer	*K*_d_, μM	k_on_, µM^−1^·s^−1^	k_off_, s^−1^	*K*_d,app_, µM (pH 7.2, 1 mM Mg^2+^)	k_on,app_, µM^−1^·s^−1^ (pH 7.2, 1 mM Mg^2+^)
EDTA	2.5 × 10^-5 a^	2,500^a^	0.7^a^	15.7^a^	0.06^a^
EGTA	1 × 10^-5 b^	2,500^c^	0.5^d^	0.18^c^	2.7^d^
BAPTA	0.17^d^	450^d^	79^d^	0.176^d^	440^d^
ATP	170^l^	150^k^	30,000^k^	2,496^l^	12^l^
PV	0.012^e,g^	100^e,g^	1^e,f^	0.16^g^	6.2
CB-D28k	0. 312^i,h^	110^i,h^	34^h^	0.434^i^	78^i^
Isolated skeletal TnC	3.3^j^	157^j^	342^j^	5.9^j^	45^j^

The listed measured equilibrium constants (*K*_d_) and rate constants k_on_ and k_off_ values apply to Ca^2+^ binding to the buffer in the presence of a range of conditions including very low H^+^ and Mg^2+^ concentrations. The apparent equilibrium constants (*K*_d,app_) were taken from measured values at 1 mM Mg^2+^, pH 7.2. The rate constants k_on,app_ were estimated under those conditions based on measured k_off_ values. Note that parvalbumin (PV), calbindin-D28k (CB-D28k), and Troponin-C (TnC) have multiple Ca^2+^ binding sites, and the k_on_ values represent the highest measured at a single site unless the dissociation constants are identical. The parameters for a single ligand can come from different studies: the measured *K*_d_ does not equal the expected ratio of the rate constants. ^a^Ref. 22, 25°C, no added Mg^2+^; ^b^Ref. 23; ^c^Ref. 24, 35°C, no added Mg^2+^; ^d^Ref. 25, 22°C, no added Mg^2+^; ^e^Ref. 26, whiting (fish) protein, k_on_ is highest of two binding sites, ∼20°C, no added Mg^2+^; ^f^Ref. 27, bovine chromaffin cells, K_d_ and k_off_ at 20°C, 0.14 mM Mg^2+^; ^g^Ref. 28, bullfrog skeletal muscle, *K*_d_ values for Ca^2+^ and Mg^2+^ (∼90 μM) [note that another study found a 2-fold lower value for K_d_(Mg) ∼40 μM (29)]; ^h^Ref. 30, recombinant protein, highest k_on_ of 2 binding sites, 20°C, no added Mg^2+^; ^i^Ref. 31, recombinant human protein, highest *K*_d_ value of 4 binding sites, 20°C; ^j^Ref. 32, isolated TnC in the absence and presence of 3 mM Mg^2+^; ^k^Ref. 33, measured in solution at 20°C in 0.1 M KCl, from a compilation of stability constants; ^l^Ref. 34, rate constants based on measurements in free solution but corrected for intracellular conditions.

The equilibria describing the reactions between protons, Mg^2+^, and Ca^2+^ with a buffer can be expressed by modifying *Eq. 1* such that

(*2*) $$[\mathrm{CaB}]={\left[B\right]}_{\mathrm{Tot}}\cdot \frac{{\left[Ca^{2+}\right]}_{i}}{K_{d,\mathrm{app}}+{\left[Ca^{2+}\right]}_{i}}$$

where

(*3*) $$K_{d,\mathrm{app}}=K_{d,\mathrm{Ca}}\cdot \left(1+\frac{{\left[Mg^{2+}\right]}_{i}}{K_{d,\mathrm{Mg}}}+\frac{{\left[H^{+}\right]}_{i}}{K_{d,H}}\right)$$

*K*_d,Ca_, *K*_d,Mg_, and *K*_d,H_ are the equilibrium dissociation constants, for Ca^2+^, Mg^2+^, and H^+^, respectively, such that *K*_d,Ca_ = k_off,Ca_/k_on,Ca_, *K*_d,Mg_ = k_off,Mg_/k_on,Mg_, and similarly for H^+^.

In other words, as [Mg^2+^]_i_ or [H^+^]_i_ increases, the apparent *K*_d_ for Ca^2+^ increases. *Equations 2* and *3* quantify the extent to which H^+^- or Mg^2+^-bound forms of the buffer will affect the overall Ca^2+^ equilibration position. The apparent equilibrium constant (*K*_d,app_) can be used to calculate the apparent association rate constant k_on,app_ = k_off_/*K*_d,app_ using the measured dissociation rate constant (k_off_), which only involves Ca^2+^ and the buffer and is therefore unaffected by Mg^2+^ or H^+^. Some examples of measurements that demonstrate the reduction of the rate constant are given in TABLE 1 and discussed in sects. 2.3 and 2.5. It should be noted that the numbers presented come from many different studies, not necessarily performed at the same temperature, pH, and ionic strength and using material from different sources. Therefore, one cannot expect quantitative agreement in all cases. As will become apparent in subsequent sections, uncertainty about Ca^2+^ binding under physiological conditions makes it difficult to predict the properties of Ca^2+^ buffering from the known concentrations of buffers, and it will be important to address this in future work.

### 2.3. Small Synthetic Molecule Ca^2+^ Buffers

Small synthetic molecule buffers have many uses in experimental biology, forming the basis of many Ca^2+^-indicator dyes (35, 36), and are used for determining or altering the free Ca^2+^ concentration of solutions (35, 37, 38). In addition, “caged” Ca^2+^ chelators can change their affinity for Ca^2+^ upon photolytic exposure to light, thereby either releasing (39–41) or chelating (42) Ca^2+^ ions. Furthermore, understanding how the different chelation structures of the small synthetic molecules correlate with changes in affinity, selectivity, and kinetics helps explain the range of properties of the various Ca^2+^-binding molecules seen in nature. The kinetics and equilibria of Ca^2+^ binding depend on the structure of the ligand relative to the ionic radius of the cation. In the case of ethylenediaminetetraacetic acid (EDTA; FIGURE 2*A*), the ligand structure chelates Ca^2+^ but also accommodates Mg^2+^, an abundant intracellular divalent cation with a smaller ionic radius and a larger solvated radius. Although the affinity of EDTA for Mg^2+^ is 100-fold lower than for Ca^2+^ cytosolic levels of Mg^2+^ are >1,000-fold higher, and thus Mg^2+^ binding dominates over Ca^2+^ in an intracellular environment. Under these conditions, buffering or binding Ca^2+^ inevitably involves displacement of bound Mg^2+^, and therefore the complete reaction scheme incorporates Mg^2+^ dissociation/association (*reaction 2A*). As discussed in sect. 2.2, this feature, together with the effect of H^+^ binding, reduces the affinity (by ∼400,000 fold) and slows the overall kinetics of Ca^2+^ buffering. Furthermore, Ca^2+^ binding to EDTA releases Mg^2+^ from the buffer molecule and potentially increases the free Mg^2+^ level. In turn, buffering by EDTA will depend on Mg^2+^, which is buffered by free ATP^2−^ and therefore depends on the metabolic state of the cell.

The related ligand ethylene glycol-bis(β-aminoethyl ether)-*N*,*N*,*N*′,*N*′-tetraacetic acid (EGTA) has a higher selectivity for Ca^2+^ over Mg^2+^ (10,000 fold) because of a longer backbone between the two amino groups that is more suited to chelate the larger ionic radius of Ca^2+^. Therefore, Mg^2+^ binding is minimal and interferes less with the Ca^2+^ buffering action. This means that k_on_ of Ca^2+^ binding to the unprotonated form of EGTA approaches the diffusion limit of 10^3^ to 10^4^ µM^−1^·s^−1^ (24). However, at neutral pH, >98.5% of EGTA is in the diprotonated form, with protons bound to the two amino groups that participate in chelation, and this form of the molecule has a much-reduced ability to bind Ca^2+^, with a *K*_d_ ∼10^5.6^ times greater than in the absence of protons and therefore not significantly contributing to chelation. Significant amounts of the unprotonated form are only available to bind Ca^2+^ metal ions after the dissociation of protons (*reaction 2B*) (25); thus the effective k_on_ for Ca^2+^ binding is ∼100-fold less than the diffusion limit because of the additional steps of proton dissociation as part of the chelation reaction. This property not only slows buffering but also makes it steeply pH dependent, and for every Ca^2+^ ion that binds to EGTA approximately two H^+^ ions are released (23). In consequence, EGTA-containing solutions will acidify upon addition of Ca^2+^. Therefore, solutions should be pH adjusted after adding Ca^2+^, and EGTA-loaded cells should be strongly pH buffered to avoid pH changes upon Ca^2+^ influx. It should be noted that around pH 7.2 a titration error of only 0.1 pH units will cause a 50–80% error in the apparent *K*_d_ of EGTA (23, 43, 44).

In contrast to EDTA and EGTA, for which the rate of Ca^2+^ binding is limited by the need for Mg^2+^ or protons to dissociate, 1,2-bis(2-aminophenoxy) ethane-*N*,*N*,*N*′,*N*′-tetraacetic acid (BAPTA) has fast Ca^2+^ binding kinetics at neutral pH (35). This molecule combines a high selectivity for Ca^2+^ over Mg^2+^ arising from the optimum backbone length and a low affinity for protons on the two amine groups, allowing it to be relatively unaffected by the Mg^2+^ concentration and pH of the solution. Ca^2+^ binding can therefore occur without any significant exchanges with Mg^2+^ or H^+^, and this explains the fast k_on_ for Ca^2+^ association (>10^2^ µM^−1^·s^−1^) (25). It is worth emphasizing that the intrinsic rate of Ca^2+^ binding by BAPTA is actually lower than for EDTA and EGTA (TABLE 1). It is the lack of competition with H^+^ or Mg^2+^ that ensures an effective fast binding rate under physiological conditions.

### 2.4. Small Biological Molecule Ca^2+^ Binding

A range of small-molecule ligands is endogenous to the cellular environment. One example is that of humic and fulvic acids, comprising a mixture of many different molecules resulting from the breakdown of organic matter, which buffer a range of environmental divalent cations including Ca^2+^, Cd^2+^, and Pb^2+^, with the *K*_d_ of humic acid for Ca^2+^ being of the order of 1 mM at neutral pH (45, 46). In biological systems, individual amino acids such as l-arginine and l-lysine have significant single-site Ca^2+^ affinities, whereas di- and tripeptides, e.g., Gly-Glu, bind Ca^2+^ with chelation structure and *K*_d_s (0.1–1 µM) similar to synthetic chelators such as EGTA and BAPTA (47). These Ca^2+^-peptide complexes may have a biological function, for example facilitating Ca^2+^ transport across the intestinal epithelium (48).

#### 2.4.1. Histidyl dipeptides, including carnosine.

Another function of Ca^2+^ binding to dipeptides is suggested by studies of the histidyl dipeptides (HDPs, e.g., carnosine) in striated muscle (49). Although possessing a relatively low Ca^2+^ affinity (*K*_d_ ∼1 mM) (50, 51), the very high intracellular levels (10–20 mM) mean that these dipeptides may bind significant amounts of intracellular Ca^2+^. However, there is uncertainty over the apparent Ca^2+^ affinity of carnosine under physiological conditions, in particular over the role of an amino group with a pK of ∼9 (50), which will be protonated at normal intracellular pH. If this is involved in chelation, Ca^2+^ binding will be negligible at normal pH (34). However, a recent study suggests that carnosine does bind Ca^2+^ with millimolar affinity at pH 7.2 (52). That carnosine can bind Ca^2+^ at physiological pH is also suggested by the observation that acidification from 7.3 to 6.7 results in the release of Ca^2+^ ions from carnosine in a mock intracellular solution (49). This pH sensitivity is, however, greater than what would be expected from measured binding constants (52). Given the high intracellular concentration of carnosine and its potential importance as a buffer, it is essential to address these issues.

#### 2.4.2. ATP.

Another important biological small-molecule buffer is ATP; this is almost entirely bound to Mg^2+^ under physiological conditions. The molecule also binds Ca^2+^ with a low affinity relative to intracellular Ca^2+^ concentrations [*K*_d_ ∼ 0.2 mM (33)], but the high intracellular concentration of ATP (∼5 mM) makes it a significant Ca^2+^ buffer in many cell types (34). Most importantly, the fact that ATP is freely diffusible enables it to facilitate diffusion of Ca^2+^ (see sects. 4.5, 6.3, 7.6, and 9.2).

#### 2.4.3. Inorganic phosphate.

$PO_{4}^{3-}$ is in equilibrium with $\mathrm{HP}O_{4}^{2-}$ and $H_{2}PO_{4}^{-}$, with the relative proportions being sensitive to pH. All forms have the ability to bind Ca^2+^ (33). At normal intracellular pH (∼7.0) the apparent *K*_d_ for Ca^2+^ binding is ∼16 mM, so for [Ca^2+^]_i_ ≤ 100 nM, the total Ca^2+^ bound to 1 mM phosphate will be ≤10 nM. In other words, phosphate makes a negligible contribution to cytoplasmic Ca^2+^ binding but may be more relevant to Ca^2+^ buffering in mitochondria (sect. 2.6.2.1) or organelles with higher [Ca^2+^] such as sarcoplasmic reticulum (SR), endoplasmic reticulum, and the nuclear envelope.

#### 2.4.4. Other intracellular anions including gluconate.

Intracellular anions include intracellular proteins (see above) and the phosphate and carbonyl groups of lipids in the inner leaflet of the plasma membrane (53, 54), as well as a series of small-molecular weight anions, the relative concentration of which varies depending on cell type. A surface array of negative charges represented by the phospholipids of the inner surface of the plasma membrane is thought to influence the physical chemistry of Ca^2+^ in cells in two ways: *1*) by contributing to intracellular buffering, which can be approximated by a contribution to global cytoplasmic binding (54) [such a role of negative surface charges has featured in models of intracellular Ca^2+^ binding (55)], but its contribution to bulk Ca^2+^ buffering is estimated to be small, and *2*) electrostatic interactions between Ca^2+^ and the surface of fixed negative charges may create a layer of 2- to 3-nm depth with a higher Ca^2+^ concentration than bulk concentration (56). This may influence the apparent affinity of Ca^2+^ binding to proteins within the membrane. Again, the influence on bulk buffering will be small apart from regions of the cell where the relative surface area of membrane to cytoplasmic volume is very high, e.g., the diadic/triadic clefts in striated muscle. Under these circumstances, in the presence of significant transmembrane Ca^2+^ fluxes, Ca^2+^ binding to nearby surface membranes may be a significant factor in determining the extent and time course of free Ca^2+^.

The inorganic anions include Cl^−^, $NO_{2}^{-}$, $NO_{3}^{-}$, $H_{2}PO_{4}^{-}$/$\mathrm{HP}O_{4}^{2-}$, and $SO_{4}^{2-}$ and the organic anions include amino acids and dipeptides, all of which associate via various forms of electrostatic interactions with the cations of the intracellular medium including Ca^2+^ and Mg^2+^ ions (57). Replicating this complex range of anions in mock intracellular solutions can be difficult, and unphysiological levels of some anions can have effects on intracellular processes, e.g., higher than normal intracellular chloride concentrations inhibit G protein-related reactions (58). Gluconate has become a popular choice for the major anion of pipette solutions in whole cell patch-clamp studies on neurons. Although earlier work had not assigned any Ca^2+^-binding capacity to this compound (59), a subsequent study (60) demonstrated low-affinity Ca^2+^ binding with a *K*_d_ of 57 mM. As shown in sect. 9.4, this cannot be neglected given the relatively high concentrations of this anion in pipette-filling solutions.

### 2.5. Structural Ca^2+^-Binding Motifs in Proteins

The reader is referred to several review articles on the general subject of Ca^2+^-binding proteins (CBPs) (61–64). Most proteins can bind Ca^2+^ at two types of sites: the electronegative sites of salt bridges and the hydrogen bonds formed between carbonyl/carboxylate side chains and an amino/imidazole residue of nearby amino acids. These interactions form loose chelate structures that bind ionized Ca^2+^ and Mg^2+^ with a range of low affinities such that the relationship between Ca^2+^ binding and concentration is approximately linear even at the millimolar levels of ionized Ca^2+^ in the extracellular space, as in the case of serum albumin (65). However, such low-affinity interactions produce little Ca^2+^ binding at cytoplasmic Ca^2+^ concentrations. At rest, intracellular [Ca^2+^] is ∼2,000- to 20,000-fold lower than extracellular, and in the presence of millimolar levels of Mg^2+^, specific Ca^2+^ binding requires precise chelate structures for selectivity and affinity. Within eukaryotes, the Ca^2+^-binding motifs of intracellular CBPs conform to a limited set of designs, discussed below in this section; variants of each form with different Ca^2+^ binding characteristics allow their use in a range of intracellular processes.

#### 2.5.1. Ca^2+^ buffering by the EF-hand domain.

The EF hand is a helix-loop-helix motif that generates a chelation structure in the loop segment (FIGURE 2*B*); many CBPs contain multiple paired EF-hand domains allowing the structure to interact with intracellular Ca^2+^ over a specific concentration range. Ca^2+^ binding to an EF-hand domain changes the tertiary structure. A commonly applied distinction is between CBPs acting solely as buffers with little conformational change upon Ca^2+^ binding and those acting as signaling molecules (“Ca^2+^ sensors”). Although this distinction is not always justified (66), for some CBPs such as parvalbumin (PV; Ref. 565) and calbindin-D_9k_ (CB-D9k), which function primarily as buffers or to facilitate Ca^2+^ diffusion, Ca^2+^ binding-induced conformational changes can be small. In contrast, many EF-hand proteins undergo large changes of tertiary structure on binding Ca^2+^, thus altering the properties of the protein containing the EF hand. This mechanism is used to control the activity of proteins in many aspects of cellular function including cell mitosis, movement, sensory function, and molecular memory. Examples include Ca^2+^ binding to the Troponin-C (TnC) subunit causing major structural changes in striated muscle thin filament proteins, thereby allowing cross-bridge activity and contraction, and Ca^2+^ binding to calmodulin (CaM), which exposes specific hydrophobic structures allowing binding to corresponding sites on the regulatory domain of CaM kinase, displacing the autoinhibitory domain and activating this enzyme. For instance, Ca^2+^/calmodulin kinase II (CaMKII) and its activation by Ca^2+^ are essential for induction of long-term synaptic plasticity and memory formation (67). The S100 protein group is a large family (25 to date) of related proteins with 2 EF-hand motifs/protein. As reviewed previously (68), their role is varied, with some entirely acting inside the cell, some with both intracellular and extracellular actions, and others with a purely extracellular role. Inside the cell, S100 proteins generally endow Ca^2+^ sensitivity to cellular processes; for example, the abovementioned CB-D9k is also known as S100G (68). Interestingly, some of the S100 proteins bind Zn^2+^, Cu^2+^, and Mn^2+^ at the interface of S100 dimers, i.e., sites distinct from the EF motifs. The sequestering or buffering of these transition metal ions modulates biological pathways; for example, Mn^2+^ binding by extracellular S100 proteins is thought to be responsible for aspects of “nutritional immunity” (see Ref. 69 for review and sect. 11).

The canonical EF motif structure has a 12-residue loop containing the amino acids aspartate and glutamate that commonly provides 4 carbonyl and 2 carboxylate groups for coordination to Ca^2+^; the seventh coordination site is provided by a water molecule hydrogen-bonded to the carboxylate of the aspartate at residue 9 (17). This structure was first discovered in PV almost 50 years ago (70), and >4,400 EF motif structures have since been identified in >1,600 proteins (71). When incorporated into a protein, the EF-hand motif exists in closely associated pairs that are not identical in structure; binding in one motif can influence the binding of the partner, resulting in cooperative Ca^2+^ binding characteristics (see sect. 3.3). CaM (72) and CB-D9k (73) are well-documented examples of this phenomenon. The two EF-hand motifs are positioned such that the Ca^2+^-binding domains face each other and in such proximity that the bound Ca^2+^ ions are only 10 Å apart (17). EF hands can have high affinities for Ca^2+^ even under intracellular conditions. For example (TABLE 1), CB-D28k has a measured *K*_d_ in the absence of Mg^2+^ of ∼0.12 µM (30, 31), which increases to ∼0.24 µM at intracellular Mg^2+^ levels.

##### 2.5.1.1. Mg^2+^ binding by the ef domain.

For the reasons outlined above, Mg^2+^ would be expected to bind to the EF-hand motif (74). Since the canonical EF motif structure has a *K*_d_ for Mg^2+^ in the millimolar range, ∼50% of binding sites have Mg^2+^ bound at resting levels of [Ca^2+^]_i_. There are two forms of site-selective interactions between Ca^2+^, Mg^2+^, and the EF hand, which render the respective sites as either *1*) Ca^2+^-selective or *2*) “Ca/Mg” sites.

*1*) Ca^2+^-selective sites: The coordination of Mg^2+^ involves only four residues on the NH_2_-terminal loop region; none of the residues in the COOH-terminal section of the loop normally participates. With this type of interaction, Mg^2+^ binding acts to stabilize the apo state (without Ca^2+^ bound), the inactive form of the EF motif. In contrast, Ca^2+^ binding recruits both NH_2_- and COOH-terminal regions of the loop and activates the motif. Therefore, as with EDTA, the presence of intracellular Mg^2+^ will slow Ca^2+^ binding and decrease its affinity at those sites (75, 76). There are other potential consequences of Mg^2+^ binding: if it is to one site of a pair of adjacent EF sites, Mg^2+^ binding can act cooperatively to enhance Ca^2+^ binding to the other site (75, 77). Therefore, the effects of Mg^2+^ binding to the COOH-terminal section of these Ca^2+^-selective sites are complex, and the consequences for the Ca^2+^ sensitivity of the proteins with canonical EF-hand sites have yet to be fully explored (76, 77).

*2*) Ca/Mg sites: In some noncanonical forms of the EF-hand motif, the sequences in the loop region form a smaller chelate structure that engages both NH_2_-terminal and COOH-terminal regions when either Ca^2+^ or Mg^2+^ is bound, and therefore both ions activate the motif; these sites are classified as Ca/Mg sites. In many instances, there is little unbound buffer, and at low intracellular Ca^2+^ levels (100 nM), the majority of the sites have either Ca^2+^ or Mg^2+^ bound. This chronically activated EF-hand domain is important for protein shape (i.e., structural sites). This is the case for the EF-hand domains of sites III and IV in striated muscle TnC, the so-called “nonspecific sites.” Both are Ca/Mg sites and, in the resting/inactive muscle, are almost fully occupied with Ca^2+^ (∼49%) or Mg^2+^ (49%) (78, 79). Sustained increases of cytoplasmic Ca^2+^ will slowly displace the bound Mg^2+^ on sites III and IV, accounting for the slow kinetics of Ca^2+^ buffering (for discussion see Refs. 80–82). One of the best-characterized Ca/Mg sites is that on PV, where the slow Ca^2+^ binding and Mg^2+^ dissociation have important consequences for skeletal muscle physiology and synaptic function and plasticity (see sects. 7.2, 9.4, and 9.5).

Both the Ca/Mg and Ca^2+^-selective sites bind Mg^2+^, but only in the former does this binding mimic that of Ca^2+^ binding. Thus, in both Ca/Mg and Ca^2+^-selective EF-hand domains, Mg^2+^ will slow the kinetics of Ca^2+^ binding and reduce affinity. Interestingly, Mg^2+^-bound states are often not included in computational models of Ca-EF hand interactions (83–86), although it has been observed that Mg^2+^ is bound to the Ca^2+^-selective sites of troponin-C and may slow Ca^2+^ binding and thence the development of force (87).

Approximately 80 different subfamilies of EF-hand domains have been recognized (88). Insertions, deletions, and substitutions in the loop region chelation site are believed to be the cause of the range of Ca^2+^ affinities that these EF-hand structure variants display, ranging from a *K*_d_ of ∼10^−9^ M seen in some CBPs such as PV to *K*_d_ values close to 10^−3^ M in the CREC, a group of ER-based proteins (89) (see sect. 2.6.1.1). A systematic study of the various EF-hand chelation structures and associated Ca^2+^ affinities across a range of EF-hand domain sequences is lacking, preventing the prediction of affinity from structure alone (89).

Almost one-third of known EF-hand structures do not bind Ca^2+^ (88), supposedly because of changes in the amino acid composition of the loop region including the substitution of the amino acids with oxygen-containing side chains. A well-documented example of a nonbinding EF-hand motif is that of site I in the striated muscle protein troponin-C. In the fast-twitch skeletal form both sites I and II bind Ca^2+^, whereas in the cardiac and slow-twitch forms only site II can do so. It has been suggested that site I cannot bind Ca^2+^ because of the disruption of the chelation site by an insertion (V28) and two key Ca^2+^-binding amino acid substitutions (D29L and D31A) (90).

##### 2.5.1.2. h^+^ influence on ef-hand buffering of ca^2+^.

As mentioned above, some small-molecule Ca^2+^ chelators have a steep dependence on pH due to the direct involvement of amino groups (with p*K*_a_ close to neutral pH) in the coordination of Ca^2+^. In contrast, the chelation structure of EF-hand domains uses only the carboxylate groups on glutamate and aspartate residues to coordinate Ca^2+^. The amino groups on other residues do not participate directly in chelation. These carboxylate groups have a p*K*_a_ of ∼2 and would therefore be expected to be fully dissociated at normal intracellular pH. Hydrogen ions, however, can form a loose association by bridging carboxylate and carbonyl groups of adjacent residues. The shared H^+^ has a p*K*_a_ of ∼6.2, close to the physiological pH range, and is thought to be one of the main causes of pH dependence of Ca^2+^ binding (91) and for the stoichiometry of ∼1 H^+^ released per Ca^2+^ bound to an EF-hand domain at neutral pH (92). This interaction between Ca^2+^ and protons also means that a decrease of pH will release Ca^2+^ from the EF hand, and this has been suggested to account for the acid-induced increase of [Ca^2+^]_i_ seen in cardiac muscle (49, 93).

#### 2.5.2. Buffering due to cytosolic Ca^2+^ binding to P-type ATPases.

The P-type transport ATPases constitute a family of proteins involved in the pumping of cations including Ca^2+^ across plasma membranes using a binding and translocation process. Although they are included in some models of Ca^2+^ dynamics in myocytes (e.g., Ref. 80), it is often overlooked that, in addition to their role in active transport, the chelation sites of these proteins contribute to the static intracellular Ca^2+^ buffering component of the cell (94). The two main forms of Ca^2+^ pumps in mammalian cells are the sarco(endo)plasmic reticulum Ca^2+^-ATPase (SERCA) and the plasma membrane Ca^2+^ ATPase (PMCA) (for reviews see Refs. 6, 95). These pumps are further divided into isoforms (SERCA1–3 and PMCA1-4), with alternative splicing generating ∼15–20 variants. The two types of Ca^2+^ pumps have a common general structure composed of E1 and E2 conformational states. The E1 state binds Ca^2+^ on the cytosolic side, and the E2 state releases Ca^2+^ on the intraluminal (SERCA) or extracellular (PMCA) side (96, 97). As part of the Ca^2+^ pump cycle, the Ca^2+^ binding to SERCA follows a sequential cooperative reaction scheme involving the two binding sites. It can be described by a Hill slope of 1.9 and a *K*_app_ ∼0.4 µM (see sect. 3.3). The two individual sites have quite different *K*_d_ values (20 nM and 8.3 µM, respectively) (98). The cooperative Ca^2+^ binding has consequences for the Ca^2+^ buffer characteristics of SERCA, reducing its buffer contribution at lower [Ca^2+^]_i_ (see sect. 3.3). The buffer characteristics of SERCA interact with its function as a pump (94, 99, 100), leading to paradoxical consequences of altering SERCA expression. For example, increased expression of SERCA increases the Ca^2+^ content of the ER/SR and therefore the amount released on stimulation, but the accompanying increased cytoplasmic buffer power may limit the rise in free Ca^2+^ levels and curtail the peak of the Ca^2+^ transient (99, 101).

In contrast to SERCA, PMCA pumps bind only one Ca^2+^ per cycle/ATP with a *K*_d_ of ∼1 µM (102). Because of this simpler reaction scheme, the PMCA has a different [Ca^2+^]-buffer power relationship (see sect. 3.3). As with SERCA, the pump turnover complicates the analysis of the overall buffer function of the PMCA. Its peripheral location and a relatively limited PMCA expression mean that the direct contribution to buffering may be small compared to the role in control of local subplasmalemma Ca^2+^ levels within cells (103).

#### 2.5.3. Ca^2+^ binding by the C2 domain.

The conserved domain 2 (C2) is another Ca^2+^ binding motif that endows Ca^2+^ sensitivity to a series of membrane-targeted signaling molecules that include membrane receptors, kinases, G proteins, and various synaptic proteins. The numbers of different C2 and EF-hand designs are comparable in eukaryotes (104). Although less studied in terms of the physical chemistry of Ca^2+^ binding than the EF-hand domain, the C2 domains are found in a range of signaling pathways including kinases that phosphorylate membrane proteins, vesicle-targeting proteins, enzymes that modify signaling phospholipids, transmembrane pore proteins, and Ca^2+^ sensors for neurotransmitter and hormone release. A C2 domain is made up of ∼140 amino acids and typically forms a three-loop structure that links two β-sheets (105), chelating normally two and sometimes three Ca^2+^ using carboxylates and carbonyl residues in adjacent regions of two loops with relatively low affinity. Binding of the second Ca^2+^ within a C2 site usually shows strong cooperativity and generates a steep Ca^2+^ dependence and a switchlike response to increased intracellular Ca^2+^ (105). The Ca^2+^ levels required to activate C2-dependent signaling tend to be higher than those for common EF-hand domain motifs (106–108). The overall lower affinity for Ca^2+^ and Mg^2+^ implies that the low-affinity C2 domains do not have significant Ca^2+^ or Mg^2+^ bound under resting conditions. The high-affinity C2 domain on the common PKC isoforms has a *K*_d_ of 1–5 µM compared to ∼20 µM on the second C2 domain (107). In summary, the C2 domains represent a chelation design used in various signaling proteins that require [Ca^2+^]_i_ in the micromolar or tens of micromolar range to activate. These concentrations of free Ca^2+^ are observed in the cytoplasm of skeletal muscle during a tetanus but otherwise are typically seen only within local Ca^2+^ domains, for instance near open Ca^2+^ channels at active zones of synapses (109) (see sects. 4.7.2 and 9.4). Therefore, C2 domains do not contribute substantially to overall Ca^2+^ buffering power but respond to local Ca^2+^ signals that are shaped by the actions of Ca^2+^ flux and other dominant buffers.

#### 2.5.4. Ca^2+^ binding to annexins.

Another common Ca^2+^ binding site that is distinct from EF-hand and C2-protein domains is the endonexin fold seen in the Annexin group of proteins (110, 111). This large group is expressed across many species and has the general property of mediating Ca^2+^-sensitive phospholipid binding that allows increased cytoplasmic Ca^2+^ to mediate events such as membrane fusion. Twelve types of annexins are expressed in mammalian cells, and although their intracellular roles have not been fully explored, their low expression levels and generally low Ca^2+^ affinity means they do not participate in intracellular Ca^2+^ buffering or diffusion (110, 111).

### 2.6. Ca^2+^ Buffering in Organelles

#### 2.6.1. ER and SR Ca^2+^ buffers.

A major role of the endoplasmic and sarcoplasmic reticulum is to release Ca^2+^ ions into the cytoplasm through inositol trisphosphate (IP_3_) and ryanodine receptors (RyRs), respectively. The free Ca^2+^ concentration in the ER and SR is typically between 100 µM and 1 mM (112–115), i.e., 1,000 to 10,000 times greater than that in the cytoplasm, thereby requiring different Ca^2+^ buffers, which are discussed below in this section.

##### 2.6.1.1. ef-hand buffers.

Reticulocalbin (44 kDa) was identified as a CBP of the ER lumen containing six EF hands (116, 117). Subsequent work showed the existence of a class of such proteins, the CREC family comprising also Calumenin, ER Ca^2+^-binding protein of 55 kDa, and Calumenin 1 (see Refs. 89, 118 for reviews). Calumenin (37 kDa) (119) binds Ca^2+^ with a low affinity (*K*_d_ ∼600 µM) at seven EF-hand sites (120). Binding of Ca^2+^ results in major changes of structure, from disordered at low Ca^2+^ to alpha-helical at higher (121). These proteins may also regulate the activity of other proteins including RyR1 in skeletal muscle (122) and SERCA2 in cardiac muscle (123). Another lumen-resident protein is Stromal Interacting Molecule (STIM 1 and 2). This uses an EF hand-based Ca^2+^ sensor to detect the decrease of luminal Ca^2+^ associated with ER-mediated Ca^2+^ release. Ca^2+^ free STIM interacts with the plasma membrane Ca^2+^ release-activated calcium channel protein ORAI1 to increase Ca^2+^ influx into the cell, a process known as store-operated calcium entry (SOCE) (see Ref. 124 for review). Because the concentration of these proteins is low in comparison to those discussed in sect, 2.6.1.2, they will not play a major role in buffering Ca^2+^ in the ER lumen (117).

##### 2.6.1.2. calreticulin, calnexin, and calmegin.

The bulk of endoplasmic reticulum Ca^2+^ buffering results from proteins with structures very different from those discussed in the previous section. Calreticulin [CRT, molecular weight (MW) 46,000] is predominantly an ER luminal CBP and molecular chaperone that promotes protein folding but also has many other functions (125). There are three distinct regions in the protein: the N globular domain, the P-arm domain, and the C domain. None of these sites conforms to EF-domain or C2 protein configurations. The N domain binds Zn^2+^ and participates in chaperone interactions. The proline-rich P arm is key to the protein folding function and contains a single Ca^2+^ binding domain with a *K*_d_ of ∼1–10 µM (126). At the high Ca^2+^ concentration of the ER lumen, this site is normally fully Ca^2+^ bound and crucial for the tertiary structure of the protein. Finally, the COOH terminal of the protein ends in a region containing highly acidic residues (35 glutamate and aspartate residues out of 50), and this region can bind up to 25 Ca^2+^ ions per molecule with a *K*_d_ of ∼1 mM (for reviews see Refs. 125, 127). The degree to which the binding reactions involve cooperative interactions is not clear. The chelation mechanism uses two acidic groups on Asp and Glu residues to bind to Ca^2+^ with a k_on_ close to the diffusion limit (∼10^3^ µM^−1^·s^−1^) and a rapid k_off_ (∼10^6^ s^−1^). CRT accounts for ∼50% of the Ca^2+^ buffering within the ER. Ca^2+^ binding to the COOH-terminal sites not only buffers Ca^2+^ but also alters the structure of this region, which in turn alters the affinity of CRT for interacting proteins (chaperones) within the ER (125, 128). Through this mechanism, the intra-ER Ca^2+^ concentration modulates the types of proteins processed (129). Measurements of free Ca^2+^ concentration within the lumen of the ER with targeted sensors suggest a maximal concentration of 300–400 µM (113, 114). The minimal Ca^2+^ concentration when the ER is depleted has not been accurately measured, as few studies have used luminal indicators with appropriate sensitivities, but concentrations lower than 5–10 µM may destabilize the tertiary structure of CRT by dissociation of Ca^2+^ from the high-affinity structural site. The ER also contains two structurally related proteins, calnexin and calmegin. These differ from calreticulin in that they are membrane bound (see Ref. 130 for review). Interestingly, in calnexin the Ca^2+^ binding C domain is exposed to the cytoplasm (131) (for review see Ref. 125), raising the possibility that a single molecule can buffer Ca^2+^ on both sides of the ER membrane.

##### 2.6.1.3. calsequestrin.

The related protein calsequestrin (CSQ; MW 44,000) is the main Ca^2+^ buffer in skeletal, cardiac, and smooth muscle sarcoplasmic reticulum and comprises two isoforms (CSQ1 and CSQ2). Aspects specific to skeletal muscle function are considered in sect. 7.5. Analogous to CRT, CSQ has a limited number (2 or 3) of high-affinity Ca^2+^ binding sites; occupancy of these is key to the tertiary structure of the CSQ monomer. However, most Ca^2+^ binding occurs at multiple low-affinity binding sites arising from the numerous aspartate and glutamate residues particularly on the COOH-terminal but also the NH_2_-terminal end of the monomer. These acidic groups can bind >40 Ca^2+^ ions per molecule, with *K*_d_ values ranging from 0.1 to 10 mM (132). The isoform CSQ2, present in slow-twitch skeletal, cardiac, and smooth muscle, has a modified COOH terminal and binds up to 20 Ca^2+^ ions per molecule (133). As with CRT, the coordination of Ca^2+^ by these low-affinity sites is via pairs of acidic resides binding a single Ca^2+^. An aspect unique to CSQ is the process through which Ca^2+^ binding to the monomer allows CSQ to polymerize and generate a structure with further Ca^2+^ binding sites (FIGURE 2*C*). This is a cooperative process, as described in sect. 3.3. In both skeletal (134) and cardiac (135, 136) muscle, in addition to buffering SR Ca^2+^, CSQ2 can interact with the RyR and thereby provide a mechanism for luminal Ca^2+^ controlling RyR opening. It can therefore be challenging to separate the consequences of CSQ2-RyR interactions from Ca^2+^ buffering effects.

Mg^2+^ can also bind to CSQ. However, evidence suggests that, unlike many chelation interactions, the Mg^2+^ binds at sites distinct from Ca^2+^ (137). The two sites interact such that Ca^2+^ binding to CSQ displaces a fraction of bound Mg^2+^ (137). In common with other CBPs that involve carbonyl-carboxylate interactions, a proton can bridge between these two residues in the absence of Ca^2+^ (p*K*_a_ ∼6.2). Therefore Ca^2+^ binding to CSQ will displace significant amounts of protons (137, 138) and may change intraluminal pH, influencing other processes including SERCA and the Ca^2+^ release channel.

#### 2.6.2. Buffering in other organelles.

The reader is referred to an earlier review for a discussion of this area (139). Here we provide brief comments on mitochondria and the nucleus.

##### 2.6.2.1. mitochondria.

Regulation of mitochondrial matrix [Ca^2+^] ([Ca^2+^]_mito_) is important since it regulates various mitochondrial enzymes, and thus ATP production (140, 141). The introduction of Ca^2+^-sensitive indicators targeted to the mitochondrial matrix has suggested that [Ca^2+^]_mito_ is normally similar to cytoplasmic [Ca^2+^]_i_, ∼100 nM (142, 143). As previously reviewed (144), there is controversy as to the extent to which mitochondrial matrix Ca^2+^ responds to brief changes of cytoplasmic Ca^2+^. For example, in cardiac muscle, some studies have reported that the cytoplasmic Ca^2+^ transients result in beat-to-beat changes of [Ca^2+^]_mito_ (145–147), whereas other work has reported little or no change of [Ca^2+^]_mito_ in response to fluctuations of cytoplasmic [Ca^2+^]_i_ (148). Even when [Ca^2+^]_mito_ transients are observed, they can decay very slowly (142), such that stimulation at normal rates results in a maintained increase of [Ca^2+^]_mito_ with little or no beat-to-beat fluctuations (see Ref. 9 for review). There are two explanations for this slow decay of [Ca^2+^]_mito_: *1*) a low activity of the mitochondrial sodium/calcium exchange (NCLX) that pumps Ca^2+^ out of the mitochondria (149) and *2*) a high mitochondrial matrix buffer power. This emphasizes the need to quantify mitochondrial Ca^2+^ buffer power.

Most measurements have been made in isolated mitochondria, often at [Ca^2+^]_mito_ greater than those thought to occur physiologically (see below); and they may be susceptible to changes resulting from mitochondrial isolation. One such study found that total Ca^2+^ concentration was ∼1,000 times greater than free in heart mitochondria (150). High Ca^2+^ buffer powers were also found in heart and liver mitochondria (151). Another study reported a value of up to ∼150,000, indicating a total mitochondrial Ca^2+^ concentration of the order of 100 mM (152). These high levels of total Ca^2+^ are thought to exist mainly as calcium phosphate crystals (153), in which case the relevance of such buffering will depend critically on the kinetics of crystal formation and dissolution. A more recent study on isolated mitochondria used changes in the free Ca^2+^ of the external fluid to calculate Ca^2+^ fluxes into and out of mitochondria and compared this with changes of [Ca^2+^]_mito_ (154). Modeling of the data (155) suggested two classes of buffers. One was tentatively attributed to phospholipids and metabolites. The other was estimated to bind Ca^2+^ in a cooperative manner and was suggested to involve annexins acting as “nucleation factors” to promote binding of Ca^2+^ with phosphate. Another study on isolated guinea pig heart mitochondria found that phosphate is a significant buffer at elevated mitochondrial Ca^2+^ but that some, unidentified, mitochondrial buffer contributes at lower [Ca^2+^]_mito_ levels (156).

Work on intact cells found that the change of [Ca^2+^]_mito_ during a cardiac cytoplasmic Ca^2+^ transient was 2–10 nM (157). The authors pointed out that if the mitochondrial Ca^2+^ buffering power was 100 (similar to that of cytoplasm) the magnitude of the measured changes of [Ca^2+^]_mito_ is consistent with the calculated contribution of mitochondria to Ca^2+^ removal from the cytoplasm (158). More precise measurements are required, but it is difficult to imagine how total mitochondrial Ca^2+^ can be measured in intact cells. Another obvious question relates to whether the mitochondrial Ca^2+^ buffering power is identical in cells from all tissues.

Given the challenges involved in direct measurements of mitochondrial Ca^2+^ buffering, estimating it from the known composition of the matrix is a useful alternative. One suggested contributor is inorganic phosphate (for review see Ref. 159). Considering the binding constants of the various forms of phosphate (33), and assuming a total matrix phosphate concentration of 1 mM, then at 100 nM [Ca^2+^]_mito_ the contribution to buffer power will be only 1.5. There is also ∼5 mM ATP present, which will provide a buffer power of 7.2. This is greater than the expected buffer power of 3.6 for the cytoplasm of cardiac muscle (see TABLE 5) because of the more alkaline pH (8.0) in mitochondria. Thus, the anticipated Ca^2+^ buffer power of the mitochondria is considerably lower than that of cytosol, and it is not obvious how the known buffers can account for some of the above measurements. Further work is required to determine the identity of mitochondrial Ca^2+^ buffers.

##### 2.6.2.2. nucleus.

One might expect the Ca^2+^ buffering of the nucleus to be different from that of cytoplasm. For example, troponin, which is a major cytoplasmic buffer in striated muscle, is absent from the nucleus and cannot contribute to buffering there. Some work has produced results consistent with lower Ca^2+^ buffering in the nucleus than in cytoplasm. For example, Ca^2+^ puffs, which are mediated by Ca^2+^ release from the ER through IP_3_ receptors and occur near the nuclear membrane, spread further in the nucleus than in the cytoplasm (160). Similar results have been seen for Ca^2+^ released from Golgi (161). The apparent diffusion coefficient for Ca^2+^ is similar in nucleus and cytoplasm (162, 163). It should, however, be noted that there is no simple relationship between Ca^2+^ diffusion and buffering: Mobile buffers can increase while fixed buffers decrease diffusion. Electron microprobe measurements have reported a higher concentration of total Ca^2+^ in the nucleus compared with the cytoplasm (164). Assuming that the free Ca^2+^ concentration is the same in both regions, this would imply greater Ca^2+^ buffering in the nucleus. The highest total Ca^2+^ concentrations were found in areas occupied by heterochromatin (condensed DNA). This is interesting, given that high (∼1 mM) Ca^2+^ has been reported to be bound to DNA and that this binding alters the structure of DNA (165). Separate studies on isolated chromosomes suggest that Ca^2+^ binding alters chromosome structure during mitosis (166). It should, however, be noted that the nuclear envelope, which contains high total Ca^2+^, invaginates the nucleus as the so-called “nucleoplasmic reticulum” (167, 168). It is possible that Ca^2+^ in this structure contributes to the high total Ca^2+^ measurements from the nucleus, whereas in the nuclear lumen total Ca^2+^ content and buffering power may in fact be lower.

The work described above does not provide quantitative measurements of nuclear Ca^2+^ buffering. Another approach is to look for differences in the concentrations of candidate Ca^2+^ buffers between nucleus and cytoplasm. For example, a study on smooth muscle cell lines derived from human aorta and jejunum characterized the distribution of various S100A proteins (see sect. 2.5.1). S100A2 and A6 were found mainly in the nucleus, whereas A1 and A4 were largely in the cytosol (169). The effects of increasing nuclear Ca^2+^ buffering have been studied in cultured hippocampal neurons by targeting parvalbumin to the nucleus. This decreased the rise of nuclear Ca^2+^ produced by electrical stimulation but had no effect on the cytoplasmic [Ca^2+^]_i_. A functional consequence of increased nuclear Ca^2+^ buffering was a change of cell morphology, in particular decreased dendrite length and complexity (170, 171). Work on human eggs and preimplantation embryos found that various CBPs including calreticulin and calsequestrin were distributed in the cytoplasm but not evident in the nucleus (172).

## 3. QUANTIFICATION OF BUFFER POWER

**FIGURE 3.**
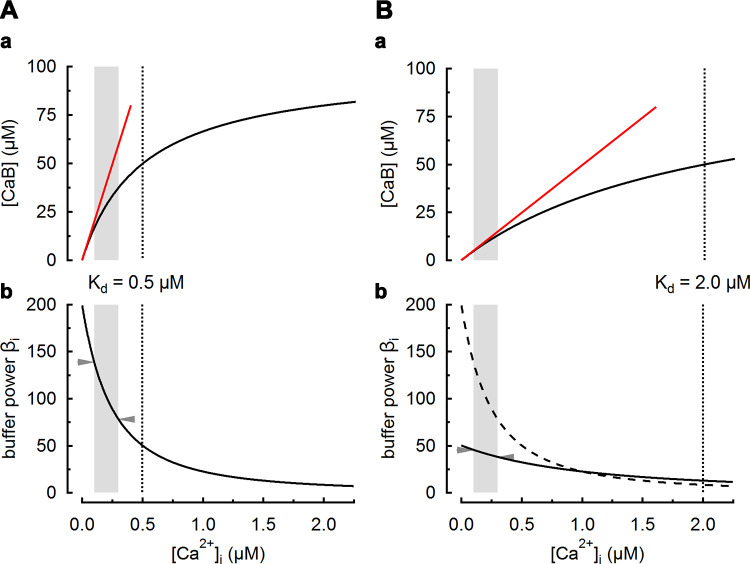
Dependence of buffer power on Ca^2+^. *A*, *a*: bound Ca^2+^ as a function of intracellular calcium concentration ([Ca^2+^]_i_). The black curve represents binding to a simple buffer according to *Eq. 1*, with an equilibrium dissociation constant (*K*_d_) of 0.5 µM (vertical dotted line). Total buffer concentration ([B]_Tot_) is 100 µM. The red line is a fit to the initial part of the curve with a slope of [B]_Tot_/*K*_d_. *b*: Buffer power as a function of [Ca^2+^]_i_. *B*: similar to *A* but for a buffer with a *K*_d_ of 2 µM (dotted line). The dashed curve in *Bb* shows the buffer power-[Ca^2+^]_i_ relationship of the buffer in *A* superimposed for comparison. The gray shaded regions in all panels represent the [Ca^2+^]_i_ range from 100 nM (resting [Ca^2+^]_i_) to 300 nM. For a [Ca^2+^]_i_ transient covering this range, buffer power is predicted to decrease by 44% (arrowheads, from 139 to 78) for the *K*_d_ of 0.5 µM (*A*) and by 17% (arrowheads, from 45 to 38) for the *K*_d_ of 2.0 µM (*B*).

The degree of Ca^2+^ buffering can be defined as “buffer power.” This is illustrated for the case of a simple buffer in FIGURE 3. For a total buffer concentration [B]_Tot_, bound Ca^2+^ ([CaB]) and free Ca^2+^ ([Ca^2+^]) are described by *Eq. 1* above.

The total Ca^2+^ concentration ([Ca^2+^]_Tot_) is given by the sum of free and bound:

(*4*) $${\left[Ca^{2+}\right]}_{\mathrm{Tot}}=\left[Ca^{2+}\right]+\left[\mathrm{Ca}B\;\right]$$

(*5*) $${\left[Ca^{2+}\right]}_{\mathrm{Tot}}=\left[Ca^{2+}\right]+\;{\left[B\right]}_{\mathrm{Tot}}\cdot \frac{\left[Ca^{2+}\right]}{K_{d}+\left[Ca^{2+}\right]}$$

Buffer power has been defined by relating changes of either total or bound Ca^2+^ to those of free. In the former case it is quantified [by analogy with the original definition of pH buffering (11, 13)] as the ratio of the change in total to that of free concentration (d[Ca^2+^]_total_/d[Ca^2+^]_free_), which is often (including this article) represented by the symbol β and is commonly used in experimental muscle physiology.

By differentiating *Eq. 5* with respect to [Ca^2+^] at constant *K*_d_ one obtains

(*6*) $$\beta \left(\mathrm{Ca}\right)=\frac{d{\left[Ca^{2+}\right]}_{\mathrm{Tot}}}{d\left[Ca^{2+}\right]}=1+\;{\left[B\right]}_{\mathrm{Tot}}\cdot \frac{K_{d}}{{\left(\left[Ca^{2+}\right]+K_{d}\right)}^{2}}$$

A slightly different definition of buffer power, often referred to in the neuroscience literature as differential Ca^2+^-binding ratio (173) and customarily designated as κ, describes the buffering by individual buffers or groups of buffers. It is the ratio of the change in bound to that of free (d[Ca^2+^]_bound_/d[Ca^2+^]_free_).

(*7*) $$\kappa \left(\mathrm{Ca}\right)=\frac{d\left[\mathrm{CaB}\right]}{d\left[Ca^{2+}\right]}={\left[B\right]}_{\mathrm{Tot}}\cdot \frac{K_{d}}{{\left(\left[Ca^{2+}\right]+K_{d}\right)}^{2}}$$

That there are two definitions is largely a consequence of differences in experimental approaches. In many studies on muscle, changes of [Ca^2+^]_i_ are compared with total changes of Ca^2+^ as measured from Ca^2+^-specific ionic currents. This corresponds to the buffer power, as defined by β (*Eq. 6*). In contrast, in the neuroscience literature buffer power is often measured by the “added buffer approach” (see below), which does not invoke total changes in Ca^2+^ but rather a comparison of effects of an endogenous buffer with those of a known buffer, usually that of a Ca^2+^-indicator dye. It should be noted (cf. *Eqs. 6* and *7*) that β is equal to 1 + κ. Therefore, for values of β or κ commonly measured in physiology (>20) there is very little difference between the two values.

As expressed in *Eq. 6*, β contains contributions from both free Ca^2+^ and Ca^2+^ bound to buffer (respectively, the first and second terms on the right-hand side). In the remainder of this review, it will be convenient to quantify the contribution to buffering of particular buffers. We therefore define β*_i_* as the contribution to buffering provided by the *i*th buffer.

(*8*) $$\beta_{i}\left(\mathrm{Ca}\right)=\;{\left[B\right]}_{\mathrm{Tot}}\cdot \frac{K_{d}}{{\left(\left[Ca^{2+}\right]+K_{d}\right)}^{2}}$$

The contributions of the *n* buffers can then be summed and added to 1 (representing free Ca^2+^) to give the overall value of β:

(*9*) $$\beta \left(\mathrm{Ca}\right)=\;1+\sum_{i=1}^{n}\beta_{i}\left(\mathrm{Ca}\right)$$

Likewise, κ can be annotated with a subscript *i* when individual buffers are considered, such as in *Eq. 29* below. β*_i_* is identical to κ*_i_*, and in this article, which aims to bring together literature across physiology, the terms are used interchangeably depending on the context.

### 3.1. Effects of Ca^2+^ Concentration on Buffer Power

*Equation 8* shows that the buffer power contribution is greatest at very low [Ca^2+^]_i_, where it can be approximated by ([B]_Tot_/*K*_d_), illustrated by the red lines that are tangents to the binding curves of FIGURE 3. As shown in FIGURE 3, *bottom*, as [Ca^2+^]_i_ increases more buffer has Ca^2+^ bound and its contribution to buffer power declines eventually to a value very close to zero at high [Ca^2+^], when effectively all binding sites are occupied. Expressed as a multiple of [B]_Tot_/*K*_d_, buffer power decreases from 1 at 0 [Ca^2+^] to 0.44 when [Ca^2+^] = 0.5 × *K*_d_ and 0.25 when [Ca^2+^] = *K*_d_. When [Ca^2+^] rises to 2 × *K*_d_, buffer power falls to 0.11.

If [Ca^2+^] changes over a finite but small range (≪*K*_d_), β*_i_* can be taken as constant and calculated at the average level of [Ca^2+^]_i_. Therefore, relating changes in total Ca^2+^ bound, ΔCaB, to those of free Ca^2+^, Δ[Ca^2+^]:

(*10*) $$\beta i\;\left(\mathrm{\Delta Ca}\right)=\;\frac{\Delta \mathrm{CaB}}{\Delta \left[Ca^{2+}\right]}$$

For larger values of Δ[Ca^2+^], β*_i_* can be replaced by (174)

(*11*) $$\kappa \text{′}=\beta_{i}\left(\mathrm{\Delta Ca}\right)=\;{\left[B\right]}_{\mathrm{Tot}}\cdot \frac{K_{d}}{\left({\left[Ca^{2+}\right]}_{1}+K_{d}\right)\cdot \left({\left[Ca^{2+}\right]}_{2}+K_{d}\right)}\;$$

Here, [Ca^2+^]_1_ and [Ca^2+^]_2_ are [Ca^2+^] values before and after the addition of Ca^2+^ ions to a buffer system as in TABLE 5 and TABLE 6. For more complex reaction mechanisms, β*_i_*(ΔCa) can be calculated from the binding curves as shown in FIGURE 3 and FIGURE 5.

Does the dependence of buffer power on Ca^2+^ concentration have any physiological significance? One can speculate that a high buffer power at low [Ca^2+^] will stabilize the low resting levels of cytosolic Ca^2+^ whereas at elevated [Ca^2+^]_i_ a smaller increase in total Ca^2+^ is required to produce a given rise of [Ca^2+^]_i_, and this may be energetically advantageous. Evidently, an increase of resting [Ca^2+^]_i_ will decrease buffer power (but see sect. 3.3.1 for discussion of cooperative buffers). This has two consequences. *1*) As discussed in the context of skeletal muscle and nerve physiology (sects. 7.4 and 9.5), an initial increase of [Ca^2+^]_i_ will decrease buffer power such that a subsequent identical increase of total Ca^2+^ will increase [Ca^2+^]_i_ more, thereby contributing to phenomena such as synaptic facilitation (175–178) and muscle contraction (82, 84, 179). *2*) As discussed in sect. 7.1, some of the variation of buffer power reported for a given tissue may reflect the range of [Ca^2+^]_i_ over which it was measured. In experimental studies, it is important to investigate whether changes of buffer power arise from alteration of the properties of the buffer or, alternatively, are a consequence of changes of [Ca^2+^]_i_. For example, a measured decrease of buffer power could be a consequence of either a change in the concentration or properties of buffers or, alternatively, an increase of resting [Ca^2+^]_i_.

### 3.2. Effects of Buffer K_d_

**FIGURE 4.**
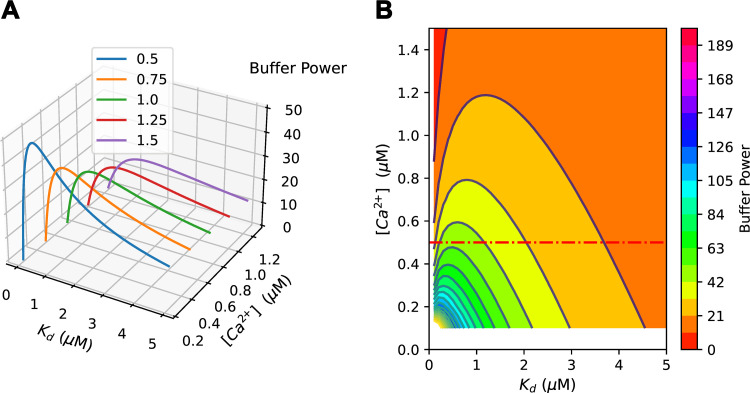
The dependence of buffer power on equilibrium dissociation constant (*K*_d_) and calcium concentration ([Ca^2+^]). *A*: 3-dimensional (3-D) plot showing buffer power (*z*-axis) as a function of *K*_d_ at the various [Ca^2+^] values indicated. A buffer concentration of 100 µM was assumed. Note that at each [Ca^2+^] buffer power increases and then decreases with increasing *K*_d_. *B*: contour plot showing buffer power as a function of K_d_ (*x*-axis) and [Ca^2+^] (*y*-axis). The horizontal dashed red line shows how buffer power changes with *K*_d_ at constant [Ca^2+^].

The total buffering produced by a given concentration of any buffer is fixed, and changing *K*_d_ simply shifts the range of [Ca^2+^] over which buffering occurs. This is exemplified in FIGURE 3, *bottom*, for two buffer species with fourfold different *K*_d_ values. Whereas the maximum β is four times larger for the high-affinity buffer (FIGURE 3*A*), β decreases more rapidly with increasing [Ca^2+^] compared to the low-affinity buffer (FIGURE 3*B*). FIGURE 4*A* shows buffer power (β) as a function of *K*_d_ at various values of [Ca^2+^]. At each [Ca^2+^], increasing *K*_d_ first increases and then decreases buffer power for that buffer (82, 180). As *K*_d_ decreases below an optimal value, the buffer becomes increasingly saturated with Ca^2+^ and its ability to buffer is decreased. As *K*_d_ increases above the optimum, less Ca^2+^ binds for a given increase of [Ca^2+^] and, again, buffer power falls. Buffer power for an individual buffer is therefore zero at both limiting low and high values of *K*_d_. This relationship can be seen in the contour plot of FIGURE 4*B* and can be examined by differentiating *Eq. 8* with respect to *K*_d_ at constant [Ca^2+^]:

(*12*) $$\frac{d\beta_{i}}{dK_{d}}={\left[B\right]}_{\mathrm{Tot}}\cdot \frac{\left(\left[Ca^{2+}\right]-K_{d}\right)}{{\left(\left[Ca^{2+}\right]+K_{d}\right)}^{3}}$$

For a given [Ca^2+^], the maximum buffer power (β*_i_*_,max_) of an individual buffer will occur when $\frac{d\beta_{i}}{dK_{d}}=0$ and therefore *K*_d_ = [Ca^2+^]. At this value of [Ca^2+^], substitution into *Eq. 8* shows that the maximum buffer power will be given by

(*13*) $$\beta_{i,\max}=\frac{{\left[B\right]}_{\mathrm{Tot}}}{4\cdot K_{d}}\;$$

Therefore, the lower the *K*_d_, the greater the maximum buffer power (cf. FIGURE 3, *bottom*). One important issue concerns the effect that a given change of *K*_d_ will have. Consider a case when a physiological or pathological change increases buffer *K*_d_ from *K*_d,1_ to *K*_d,2_. From *Eq. 8* the buffer power will be identical when

$$\frac{K_{d,1}}{{\left(\left[Ca^{2+}\right]+K_{d,1}\right)}^{2}}=\frac{K_{d,2}}{{\left(\left[Ca^{2+}\right]+K_{d,2}\right)}^{2}}$$

This is satisfied when [Ca^2+^] equals the geometric mean of the two values of *K*_d_, i.e.,

(*14*) $$\left[Ca^{2+}\right]=\sqrt{K_{d,1}\cdot K_{d,2}\;}$$

For example, when comparing β_i_ for various [Ca^2+^] for the two buffers exemplified in FIGURE 3, it is seen that their buffer power is identical at [Ca^2+^] = 0.5 μM×2.0 μM = 1 μM (FIGURE 3*Bb*). Below this level of [Ca^2+^] the increase of *K*_d_ from 0.5 to 2 µM will decrease buffer power, and above it buffer power will increase. As illustrated below (see FIGURE 10 and sect. 4.3), changes of buffer *K*_d_ are predicted to have complicated effects on the kinetics of decay of the [Ca^2+^] transient, depending on whether the level of [Ca^2+^] is below or above the geometric mean of the *K*_d_s.

### 3.3. Buffers with Cooperative Ca^2+^ Binding

Above, we have described the features of simple interactions between Ca^2+^ ions and small buffer molecules and the independent binding of Ca^2+^ ions to larger molecules. Frequently, however, Ca^2+^ binding to multiple sites on a protein is either positively or negatively cooperative: binding one Ca^2+^ ion enhances or diminishes the affinity of an adjacent site during a subsequent binding step. This type of interaction is commonly seen with Ca^2+^ binding to buffers such as EF-hand proteins and Ca^2+^ pump proteins. Both sequential and independent schemes can be summarized by *reactions 3A* and *3B*:

reaction 3A $${\text{CaB}}_{a}▂{\text{B}}_{b}\overset{K_{\text{d}(a)}}{⇌}{\text{B}}_{a}▂{\text{B}}_{b}+\text{ Ca}$$

reaction 3B $${\text{B}}_{a}▂{\text{B}}_{b}\text{Ca}\overset{K_{\text{d}(b)}}{⇌}\;{\text{B}}_{a}▂{\text{B}}_{b}+\text{ Ca}$$

B*_a_* and B*_b_* are two sites on the same protein with individual affinities described by *K*_d(_*_a_*_)_ and *K*_d(_*_b_*_)_. When two binding sites are in close proximity, the binding of Ca^2+^ to an array of negative charges may be expected to reduce the chances of a second Ca^2+^ binding purely on the basis of electrostatics, thus causing negative cooperativity (17). The fact that positive cooperativity is commonly seen in adjacent Ca^2+^ binding sites means the effect of favorable structural changes evoked by binding of the first Ca^2+^ ion must outweigh the electrostatic effects. Cooperative Ca^2+^ binding may also arise from interactions between linked protein molecules, for example in the case of Troponin-C (TnC), where binding of Ca^2+^ to one TnC will change the structure of the thin filament and increase binding to other TnC proteins bound to adjacent sites on the thin filament (32, 181, 182).

In many cases, the properties of the individual binding sites are unknown and the cooperative binding can be usefully approximated by *reaction 4*.

(reaction 4) $$n\text{Ca }+\text{ B}⇌{\text{BCa}}_{n}$$

As discussed previously (183), this can be described mathematically by the Hill equation (*Eq. 15*) (184). Note that in *Eqs. 15*–*28* we abbreviate [Ca^2+^] to Ca for simplicity:

(*15*) $$\frac{\mathrm{BC}a_{n}}{{\left[B\right]}_{\text{Tot}}}=\;\frac{Ca^{n}}{K_{\mathrm{app}}^{n}+Ca^{n}}$$

Here, *K*_app_ is the concentration of Ca^2+^ at which half the binding sites are occupied. *n* is the Hill slope, which represents the degree of cooperativity, with a value of 1 meaning no cooperativity. For a molecule with two sites that bind Ca^2+^ cooperatively, *K*_app_ and *n* can be calculated from the individual dissociation constants [*K*_d(_*_a_*_)_ and *K*_d(_*_b_*_)_] as follows (185): $K_{\mathrm{app}}=\sqrt{K_{d(a)}\cdot K_{d(b)}}$ and $n\mathrm{=2/}\left(\mathrm{1+}\sqrt{K_{d(a)}/K_{d(b)}}\right)$. In the special case where *K*_d(_*_b_*_)_ ⪢ *K*_d(_*_a_*_)_, it can be seen that *n* approaches 2. We have used this method to calculate values of *K*_app_ and *n* in TABLE 2.

**Table 2.** Examples of individual K_d_ values from pairs of EF-hand domains in different Ca^2+^ binding proteins along with the associated K_app_ value and the Hill slope

Buffer Name	*K*_a_, µM	*K*_b_, µM	K_app_, µM	Hill Slope	Ref.
CB-D28K	0.41	0.24	0.31	1.1	30
CB-D9k	0.31	0.15	0.21	1.2	186
CR	2.8	0.068	1.38	1.5	185
CaM (COOH terminus)	28	0.26	2.7	1.8	187
CaM (NH_2_ terminus)	193	0.79	12.7	1.9	187
SERCA	8.3	0.02	0.41	1.9	98

*K*_a_ represents the equilibrium constant for the initial binding and *K*_b_ the equilibrium constant created by the cooperative interaction. CalbindinD9k (CB-D9k) has 1 pair of Ca^2+^-binding EF-hand motifs and calretinin (CR) and calbindinD28k (CB-D28K) have 2 pairs; the values listed apply to both pairs in that molecule. Note that CR has an additional functional EF-hand site that is not described here. The pairs of EF-hand domains situated at the COOH- and NH_2_-terminal ends of calmodulin (CaM) are shown separately. SERCA, sarco(endo)plasmic reticulum Ca^2+^-ATPase.

Ca^2+^ binding domains can exist in pairs, and in many CBPs multiple pairs of domains exist. The structure and therefore function of these domains are not identical; some differ to the extent that Ca^2+^ cannot bind to one of the pairs of sites, in other domains Mg^2+^ or Ca^2+^ can bind and engage (Ca/Mg sites), and in others Mg^2+^ can bind but not activate the site (Ca^2+^-selective sites). In the case of some well-studied CBPs such as calmodulin (4 EF sites) (72), calretinin (5 EF sites) (185), and calbindin-D_9k_ (2 EF sites) (186), the cooperativity is between the two adjacent EF-hand domains. TABLE 2 shows examples of several CBPs and the varying extent of cooperativity that has been measured. Detailed information on all individual Ca^2+^ binding sites in a single protein is only available for a few members of the large family of CBPs. More commonly, only general descriptions of Ca^2+^ binding in terms of the Hill *K*_app_ and slope value have been reported, and we now consider cooperative buffering in these terms.

#### 3.3.1. The effects of cooperativity on buffer power.

**FIGURE 5.**
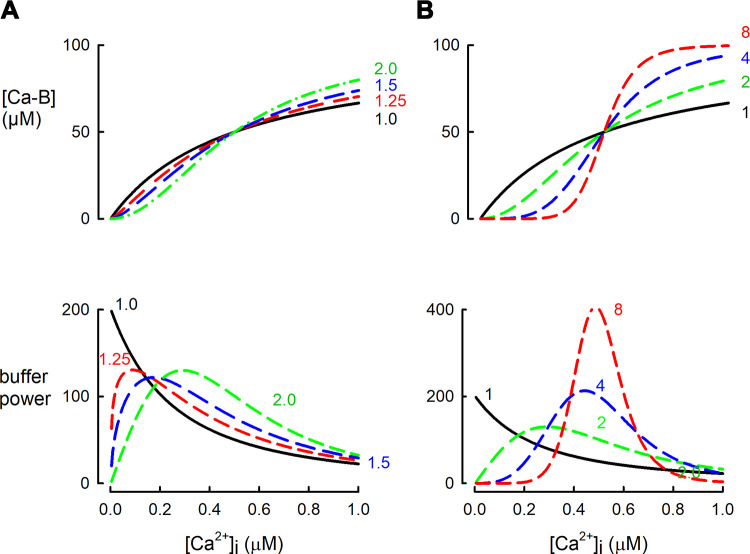
Dependence of buffer power on intracellular calcium concentration [Ca^2+^]_i_ for buffers with cooperative Ca^2+^ binding. *A* and *B*: bound Ca^2+^ (*top*) and buffer power (*bottom*), both as a function of [Ca^2+^]. All buffers are present at a concentration giving a total of 100 µM binding sites and have a [Ca^2+^] at which half the binding sites are occupied (*K*_app_) of 0.5 µM. *A*: cooperative binding described by the Hill equation with values of Hill slope (*n*) of 1.0, 1.25, 1.5, and 2.0. *B*: comparison of higher values of *n* (4 and 8) with 1 and 2.

The effects of a cooperative Ca^2+^ binding scheme on the relationship between free [Ca^2+^], Ca^2+^ binding, and buffer power are shown in FIGURE 5. As the Hill slope (*n*) value increases, so does the steepness of the dependence of bound on free Ca^2+^ (FIGURE 5, *top*). An instructive comparison is between *n* values of 1.0 and 1.25 (FIGURE 5*A*). The difference in the binding curves (FIGURE 5*A*, *top*) would be difficult to distinguish experimentally, at least with the techniques applied to intact cells and tissues. However, it has marked effects on the dependence of buffer power on [Ca^2+^] (FIGURE 5*A*, *bottom*).

The effects of cooperative binding on Ca^2+^ buffering have been considered previously (188). Here, we calculate the buffer power as a function of [Ca^2+^] by differentiating *Eq. 15*:

(*16*) $$\beta_{i}\left(\mathrm{Ca}\right)=\frac{\mathrm{dCaB}}{\mathrm{dCa}}={\left[B\right]}_{\text{Tot}}\cdot \frac{n\cdot K_{\mathrm{app}}^{n}\cdot Ca^{n-1}}{{\left(K_{\mathrm{app}}^{n}+Ca^{n}\right)}^{2}}$$

The dependence of the buffer power on [Ca^2+^] can be shown by differentiating β*_i_* with respect to [Ca^2+^] to obtain

(*17*) $$\frac{d\beta_{i}}{\mathrm{dCa}}={\left[B\right]}_{\mathrm{Tot}}\cdot \frac{n\cdot K_{\mathrm{app}}^{n}\cdot Ca^{n-2}\cdot \left[\left(n-1\right)\cdot K_{\mathrm{app}}^{n}-\left(n+1\right)\cdot Ca^{n}\right]\;}{{\left(K_{\mathrm{app}}^{n}+Ca^{n}\right)}^{3}}$$

When dβ*_i_*/dCa = 0, buffer power (β) will have a maximum value, which will be obtained at a value of [Ca^2+^] given by

(*18*) $${\left[Ca^{2+}\right]}_{i}={\left(\frac{n-1}{n+1}\right)}^{1/n}\cdot K_{\mathrm{app}}$$

Consistent with *Eq. 8*, this predicts for *n* = 1 that the highest buffer power is obtained at zero [Ca^2+^]. In contrast, when the buffer is cooperative (*n* > 1), buffer power is low at low [Ca^2+^] and peaks at intermediate [Ca^2+^], before decreasing to zero at higher [Ca^2+^] (188) (FIGURE 5). Two other conclusions can be derived from FIGURE 5: *1*) An increase of *n* reduces the range of [Ca^2+^] over which buffering occurs. Since the total amount of Ca^2+^ bound is unaffected, this means that the maximum buffer power is greatest at higher values of *n*. Indeed, for *n* = ∞ (not shown), the buffer power curve will have an infinitely high and narrow peak. *2*) The value of [Ca^2+^] at which the maximum buffer power is obtained increases with *n*, approaching a value of *K*_app_ at high *n*.

As regards the dependence of buffer power on *K*_app_, one can differentiate *Eq. 16* with respect to *K*_app_ and obtains

(*19*) $$\frac{d\beta_{i}}{dK_{\mathrm{app}}}={\left[B\right]}_{\mathrm{Tot}}\cdot n^{2}\cdot Ca^{n-1}\cdot K_{\mathrm{app}}^{n-1}\frac{\left(Ca^{n}-K_{\mathrm{app}}^{n}\right)}{{\left(K_{\mathrm{app}}^{n}+Ca^{n}\right)}^{3}}$$

For a given [Ca^2+^]_i_, the maximum buffer power is obtained when this derivative is equal to zero. Irrespective of the value of *n*, this occurs when *K*_app_ = [Ca^2+^]. Substituting into *Eq. 16* and expressing as buffer power (β) gives

(*20*) $$\beta_{i,\max}=\frac{{\left[B\right]}_{\mathrm{Tot}}\cdot n}{4\cdot K_{\mathrm{app}}}$$

In other words, the maximum buffer power (at [Ca^2+^]_i_ = *K*_app_) is proportional to *n*. For the simple case of *n* = 1 (no cooperativity), this reduces to *Eq. 13*.

It can also be shown that *Eq. 14* holds for cooperative buffers. If *K*_app_ increases from *K*_app,1_ to *K*_app,2_, there will be no change of buffer power at a [Ca^2+^] given by the geometric mean ($\sqrt{K_{\mathrm{app},1}\cdot K_{\mathrm{app},2}}$).

**FIGURE 6.**
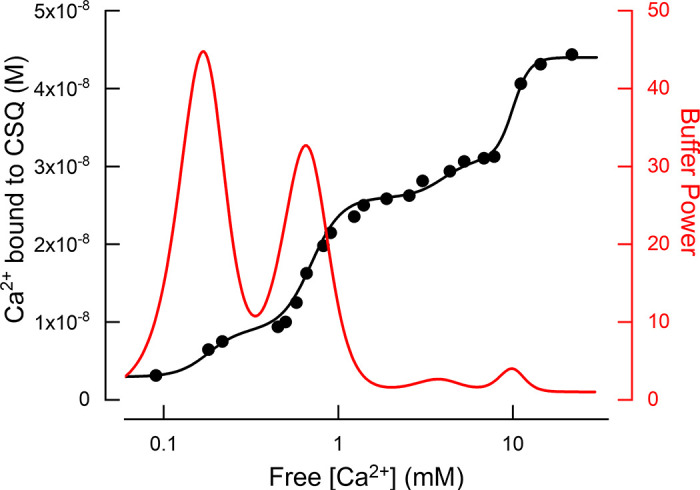
Ca^2+^ dependence of buffering by calsequestrin (CSQ). The data points featured are measurements of Ca^2+^ binding to CSQ at different free calcium concentration ([Ca^2+^]) by Park et al (133). The solid black line represents a multiple summed sigmoidal relationship that best fit the data. The red line is the buffer power calculated from the black fit. Note that the multiphasic steep relationship results in limited epochs of Ca^2+^ buffer power over the range of luminal [Ca^2+^] expected.

A good example of the effects of cooperativity on buffer power is provided by calsequestrin (CSQ). At [Ca^2+^] < 50 µM, CSQ exists as monomers; an increase of free Ca^2+^ causes binding to acidic residues at the NH_2_ terminal and COOH terminals, changing their tertiary structure allowing NH_2_-to-NH_2_ terminal and COOH-to-COOH terminal region binding. This results initially in the formation of dimers, then as [Ca^2+^] increases tetramers, and at the highest Ca^2+^ concentration (above ∼5 mM) polymers. Recent work suggests that CSQ forms polymers of helically arranged CSQ monomers (138). This change in tertiary structure exposes further binding sites on the outer surface and the water-filled lumen of the helix. This highly cooperative reaction scheme means that, unlike the case of a noncooperative buffer, the buffer power of CSQ increases with Ca^2+^ binding. As shown in FIGURE 6, buffer power calculated from published data on Ca^2+^ binding to CSQ (133) shows a complex relationship between luminal Ca^2+^ concentration and Ca^2+^ buffer power as a consequence of the multiphasic and cooperative binding curve. The extent and kinetics of this reaction are not fully understood. There are no values for *n* in the literature, but a value of 10 has been used in modeling (189), and values of 5–9 fit the data of Park et al. (133) (see FIGURE 6). The properties of CSQ are discussed more fully in the context of skeletal muscle (sect. 7.5).

For the different CBPs listed in TABLE 2, the cooperativity arises from the interaction between the two adjacent EF-hand domains on the same protein or, in the case of the binding sites on SERCA, from a sequential reaction scheme. For Ca^2+^ binding to isolated fast-twitch skeletal TnC, the adjacent EF-hand domains (sites I and II) have distinct *K*_d_ values (affinity of site II is ∼10 fold higher than that of site I) as a consequence of sequential cooperative binding (32, 190), i.e., Ca^2+^ binding to site II is necessary to generate the higher-affinity structure of site I. Note that slow-twitch skeletal and cardiac TnC binds Ca^2+^ only to site II, as site I is nonfunctional (181). Therefore, the cooperativity of Ca^2+^ binding to slow-twitch/cardiac TnC observed when TnC is bound to the troponin-thin filament-myosin complex (191) is a consequence of long-range effects of Ca^2+^ TnC binding transmitted through the thin filament to adjacent TnC sites. Other examples of sequential cooperative schemes can be seen in intraorganelle proteins such as calreticulin and calsequestrin, which can bind up to 20 Ca^2+^ ions per molecule (see sects. 2.6.1 and 7.5).

As mentioned above, detailed kinetic parameters for cooperative Ca^2+^ binding exist only for a few CBPs. A study of Ca^2+^ binding to calretinin revealed two pairs of sites with cooperative interactions and a single independent site (185). The cooperative sites consisted of a low-affinity rapid binding and a higher-affinity slower binding site. A protein with such kinetically heterogeneous sites will maintain rapid Ca^2+^ binding kinetics across a wide range of baseline Ca^2+^ concentrations, a feature not possible in simpler buffer systems (185). This unusual buffer feature is consistent with the experimental observation that calretinin modifies the time course of the IP_3_-evoked Ca^2+^ transient in ways that cannot be reproduced by either BAPTA or EGTA (192) and illustrates the importance of understanding other intracellular Ca^2+^ buffers in detail to fully appreciate the functional consequences of buffer action.

## 4. THE EFFECTS OF Ca^2+^ BUFFER PROPERTIES ON Ca^2+^ SIGNALING

### 4.1. Effects of Buffers on [Ca^2+^]_i_

**FIGURE 7.**
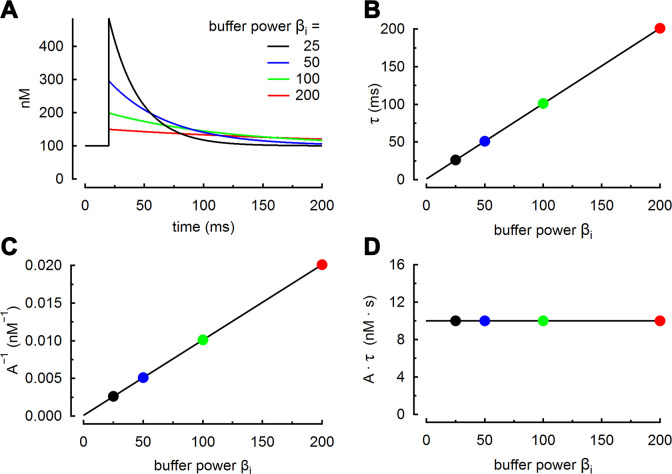
Simulation of the effects of altering buffer power (β*_i_*) on amplitude and decay of a cytosolic intracellular calcium concentration ([Ca^2+^]_i_) transient. *A*: [Ca^2+^]_i_ is controlled by a constant Ca^2+^ leak into the cell that is balanced by a Ca^2+^ pump with an activity equal to γ × [Ca^2+^]_i_ where γ = 1,000 s^−1^. At 20 ms, 10 µmol of Ca^2+^ was added per liter of cytosol. The 4 traces denote simulations performed with the buffer powers indicated. A very low-affinity buffer was assumed. Note that increasing β*_i_* decreases the amplitude and slows the decay of the transient. The area under the [Ca^2+^]_i_ transient above resting [Ca^2+^]_i_ of 100 nM is unchanged (see also *Eqs. 30–32*). *B–D*: predicted dependence on β*_i_* of decay time constant τ (*B*); reciprocal of the [Ca^2+^]_i_ transient amplitude A (*C*); and the product A × τ (*D*). Symbols represent the respective values for the sample traces in *A* with corresponding colors. The lines in *B* and *C* intersect with the *x*-axis at a value of β*_i_* of −1.

It is important to consider the roles that Ca^2+^ buffers play in the regulation of cytoplasmic Ca^2+^ concentration ([Ca^2+^]_i_). Buffers decrease the change of [Ca^2+^]_i_ produced by a given flux of Ca^2+^. A similar effect can be produced by transport of Ca^2+^ into organelles, and the combined effects have been referred to as “muffling” (193). In this review, however, we are concerned primarily with physicochemical buffering as opposed to transport into and out of intracellular organelles. It is important to note that, in contrast to the use of the term “buffer” in chemistry, Ca^2+^ buffers cannot alter the steady-state level of [Ca^2+^]_i_ in an intact cell. At steady state, the level of [Ca^2+^]_i_ reflects a simple balance between Ca^2+^ entry and efflux at the surface membrane (194–196), whereas buffers are equilibrated with Ca^2+^ according to their *K*_d_. A caveat applies to whole cell patch-clamp recordings during which the intracellular medium is connected to a large volume of buffered pipette solution, which can influence the steady state [Ca^2+^]_i_. What buffering does under normal cellular conditions is to slow down and decrease the effects of changes of total Ca^2+^ on changes in free Ca^2+^. FIGURE 7 simulates the changes in free Ca^2+^ resulting from a bolus of Ca^2+^ entering the cytoplasm. [Ca^2+^]_i_ increases in a steplike fashion and subsequently decays back to its basal value. It is assumed that Ca^2+^ is removed from the cytosol by a plasma membrane Ca^2+^ pump with activity proportional to [Ca^2+^]_i_. Four scenarios are shown, for β*_i_* equal to 25, 50, 100, or 200. In all cases, it is assumed that Ca^2+^ binds instantaneously to the buffer and that diffusion of Ca^2+^ and buffer across the volume of interest is rapid. Higher buffer power strongly attenuates the rise of free [Ca^2+^]_i_. Equally important is the prominent slowdown of the decay, since the pump will have to remove not only free Ca^2+^ but also the Ca^2+^ that is dissociating from buffers while free Ca^2+^ decays back to baseline values. Decay time constant τ and inverse of the peak amplitude (A^−1^) are plotted as a function of β*_i_* in FIGURE 7, *B* and *C*, respectively. These plots illustrate that the fraction by which the decay is slowed is identical to that by which the amplitude is reduced (see also sect. 4.7.1.1 and *Eqs. 30–32*). If Ca^2+^ removal is a linear function of [Ca^2+^]_i_, then the area under the curve (time integral A × τ) and correspondingly the time-averaged [Ca^2+^]_i_ during the transient will be unaffected by the presence of buffers (FIGURE 7*D*). The effects of experimental alteration of buffer power on Ca^2+^ transients are illustrated in FIGURE 12, FIGURE 16B, and FIGURE 21A (see also sect. 9.7 for a discussion of the effects of changing the concentration of the buffer calretinin on the changes of [Ca^2+^]_i_). The impact of buffering on processes regulated by [Ca^2+^]_i_ will depend on the activation mechanisms of these processes. For a process that is activated linearly with [Ca^2+^]_i_, the presence of a buffer will not alter the cumulative effect of Ca^2+^ transients. If the Ca^2+^-regulated process has a steep supralinear dependence on [Ca^2+^]_i_, then increased buffering will decrease the end effect, since high Ca^2+^ concentrations are ablated. On the other hand, a process with a saturating Ca^2+^ dependence will actually be enhanced by the presence of a buffer, since the latter attenuates the amplitudes of the [Ca^2+^]_i_ transients, thereby preventing saturation of the Ca^2+^-dependent process, while prolonging the duration of Ca^2+^ action.

For these conclusions to be quantitatively correct, it has to be assumed that the on and off rate constants are infinitely fast such that bound and free Ca^2+^ are always in equilibrium, that diffusion of buffers and Ca^2+^ across the cellular dimensions is rapid, and that buffering power is constant over the relevant range of changes in [Ca^2+^]_i_ (see sects. 4.2 and 4.7.1).

#### 4.1.1. Effects of buffers on the calculations of fluxes.

Fluxes of Ca^2+^ across cell membranes are often estimated by measuring the resulting changes of [Ca^2+^]_i_, and it is often overlooked that changes of Ca^2+^ buffering will alter the change of [Ca^2+^]_i_ produced by a given flux. For example, an increase of buffer power will attenuate the increase of [Ca^2+^]_i_ produced by a given influx of Ca^2+^ through a membrane Ca^2+^ channel. Furthermore, the activities of Ca^2+^ removal processes are often assessed from the time constant of decay of [Ca^2+^]_i_. However, as shown in FIGURE 7, *A* AND *B*, this time constant is also affected by Ca^2+^ buffering. This raises the possibility that an increase of the time constant of decay of [Ca^2+^]_i_ could result from an increase of buffering as opposed to a decrease of pumping (197). Given these issues, it is best to calculate Ca^2+^ fluxes from changes of total Ca^2+^ estimated from the buffer properties and changes of [Ca^2+^]_i_ (80, 197).

### 4.2. Effects of Buffer Kinetics

**FIGURE 8.**
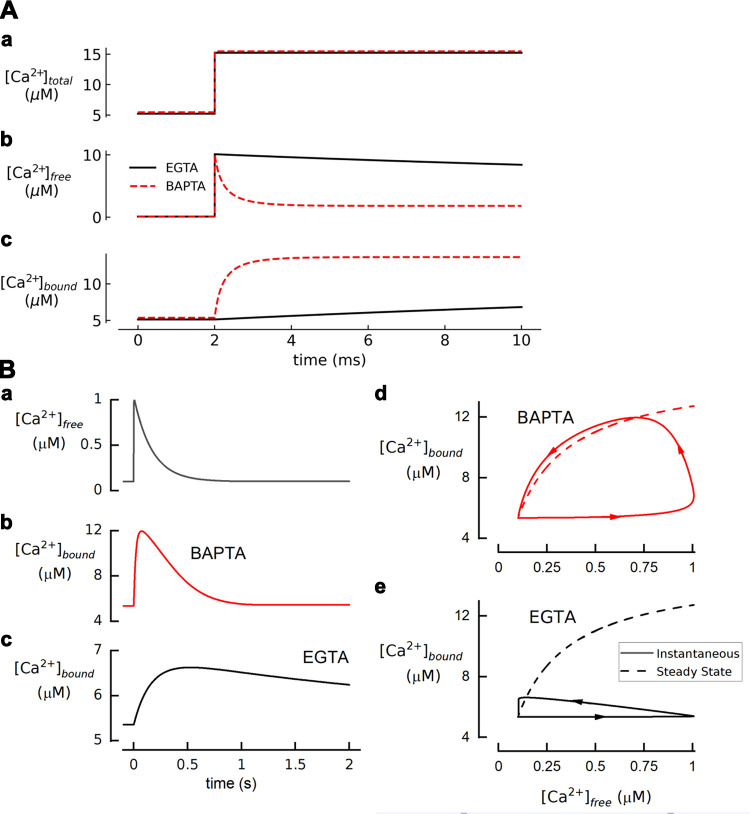
Effects of buffer kinetics. *A*: response to a bolus of total Ca^2+^. *a*: Total Ca^2+^ was increased by 10 µM. *b*: Free calcium concentration ([Ca^2+^]_free_). *c*: Ca^2+^ bound to buffer. Simulations are shown for both EGTA (black solid lines) and BAPTA (red dashed lines). Note the transient overshoot of intracellular [Ca^2+^] ([Ca^2+^]_i_) for both buffers, the decay of which is scarcely apparent at this timescale for the case of EGTA. *B*, *a*: a transient rise of [Ca^2+^]_i_ was imposed. *b* and *c*: The calculated change of Ca^2+^ bound to BAPTA (*b*) and EGTA (*c*). The solid lines in *d* and *e* show hysteresis plots of bound Ca^2+^ plotted as a function of free Ca^2+^ calculated from the responses shown in *b* and *c*, respectively. The arrows indicate the direction of time. The dashed lines show the steady-state relationship between free and total Ca^2+^.

It is important to distinguish between instantaneous and steady-state buffering. As shown in *reaction 1*, a simple buffer can be characterized by binding (k_on_) and unbinding (k_off_) rate constants, which determine the time over which buffering occurs. FIGURE 8*A* shows the response of both free and bound Ca^2+^ to a step increase of total [Ca^2+^] for buffers with a similar *K*_d_ but different rate constants. For simplicity we assume that there is no pumping of Ca^2+^ and therefore changes of [Ca^2+^]_i_ are determined solely by Ca^2+^ binding to and Ca^2+^ unbinding from the buffer. The resulting time courses of total, free, and bound Ca^2+^ are shown for a slow (EGTA) and a fast (BAPTA) buffer. In both cases, all added Ca^2+^ initially appears as free Ca^2+^. As Ca^2+^ progressively binds to the buffer, [Ca^2+^]_i_ decreases, and this occurs more quickly with the faster buffer. Finally, in the case of BAPTA, [Ca^2+^]_i_ approaches a steady state within a few milliseconds, at which it is in equilibrium with the bound state according to *Eq. 1*. EGTA is sufficiently slow that [Ca^2+^]_i_ is still declining at the end of the period shown. Two further points are worth noting: *1*) The transient overshoot of [Ca^2+^]_i_ will only occur if the rise of total Ca^2+^ is very fast relative to k_on_; if slower, the fast phase of decay of free Ca^2+^ will be obscured. *2*) Even if the fast phase of decay does occur, the ability to detect it will require a fast Ca^2+^ indicator and a fast acquisition rate.

It is also instructive to see how Ca^2+^ binding to a buffer tracks a rise of free [Ca^2+^]. Comparison of FIGURE 8, *Bb* AND *Bc*, shows that binding to even the fast buffer BAPTA lags slightly behind free Ca^2+^ and the lag is much greater for EGTA. The hysteresis curves (FIGURE 8, *Bd* AND *Be*) plot bound as a function of free Ca^2+^, both for the rise and fall of [Ca^2+^]. For BAPTA, the hysteresis curve only deviates markedly from the steady-state relationship during the very fast rise of free [Ca^2+^]. In contrast, for the slower buffer EGTA, there is a marked difference between the instantaneous and steady-state relationships throughout. FIGURE 8*Be* also shows that less Ca^2+^ is bound at a given free [Ca^2+^] during the transient compared to the steady state (when bound and free Ca^2+^ are in equilibrium), indicating that EGTA buffers more weakly than BAPTA during a transient.

**FIGURE 9.**
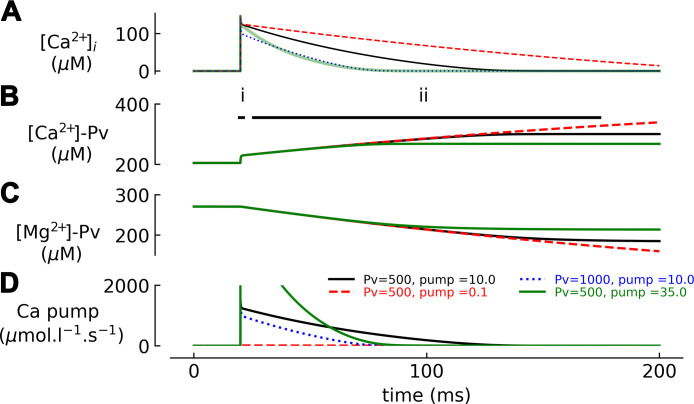
Comparison of a slow Ca^2+^ buffer with a Ca^2+^ pump. All simulations use a slow buffer, here taken to be parvalbumin (PV) at the concentrations indicated (500 or 1,000 µM). Steady-state intracellular calcium concentration ([Ca^2+^]_i_) was controlled by a leak flux opposed by a pump. Pump rate is given by pump·[Ca^2+^]_i_, where the rate constant of pump (units of s^−1^) is defined in the key. The leak was adjusted to give a resting [Ca^2+^]_i_ of 100 nM in all cases. Total Ca^2+^ was increased by 150 µM at 20 ms. *A*: [Ca^2+^]_i_. *B*: Ca^2+^ bound to buffer. *C*: Mg^2+^ bound to buffer. *D*: Ca pump rate. The fast and slow phases of Ca^2+^ binding are denoted by *i* and *ii*. Note that the data for 1,000 µM PV case are off the top of the range shown in *B* and *C*.

As discussed in sects. 2.2 and 2.5.1.1, most slow kinetics arise not from intrinsically slow kinetics of Ca^2+^ binding but rather from the time it takes for Mg^2+^ or H^+^ to dissociate, making binding sites available for Ca^2+^. If [Ca^2+^]_i_ increases, free Ca^2+^ can quickly bind only to free buffer, which may be a small fraction of the total. By mass action, the resulting decrease in concentration of the free buffer will decrease the concentration of the Mg^2+^- or H^+^-bound forms, thereby regenerating free buffer for Ca^2+^ to bind to. Thus, the steady-state buffering is much greater than the instantaneous one. The speed at which this occurs is limited by the rate at which the competing ions dissociate. This can result in two phases of Ca^2+^ binding: rapid binding to the free buffer and delayed binding to that which had Mg^2+^ bound. This is illustrated in FIGURE 9, which also shows how a slow buffer (here PV as an example) and a Ca^2+^ pump combine to promote relaxation of [Ca^2+^]_i_. The results should be compared with the simulation of FIGURE 7 for fast buffering. The simulation illustrated by the black solid line in FIGURE 9 assumes a total concentration of 500 µM PV. Initially, only 25 µM PV is free, with 205 and 270 µM, respectively, bound to Ca^2+^ (FIGURE 9*B*) and Mg^2+^ (FIGURE 9*C*). The addition of 150 µM total Ca^2+^ results in a rise of [Ca^2+^]_i_ (FIGURE 9*A*), leading to a rapid increase of Ca^2+^-bound PV (FIGURE 9*B*) (over the period indicated by *i*), as Ca^2+^ binds to available free PV. The kinetics of this increase are determined by the binding and dissociation rate constants of Ca^2+^ with PV. This is accompanied by an initial rapid fall of [Ca^2+^]_i_. A slower phase (*ii*) then ensues when Mg^2+^ dissociates from Mg^2+^-bound PV (FIGURE 9*C*) in exchange for Ca^2+^ binding (FIGURE 9*B*). There is also a decrease of free PV (not shown). On a longer timescale, beyond the period shown, the concentrations of all species will return to the initial levels. Such biphasic decays have been demonstrated when PV was added to adrenal chromaffin cells (27) and also recorded in cardiac muscle when slow buffers were added to the cytoplasm (198, 199).

FIGURE 9 also serves to compare the effects of PV with those of a Ca pump. The red dashed lines show the effects of reducing the pump to 1%. [Ca^2+^]_i_ still decreases, and this is accompanied by larger changes of bound Mg^2+^ and Ca^2+^ indicating that PV is compensating for the loss caused by the pump. The two final simulations show that either increasing PV (dotted blue lines) or the pump (green solid lines) can accelerate the slow decay component of [Ca^2+^]_i_ to similar levels, with the former resulting in more Ca^2+^ binding to PV and the latter in less. FIGURE 9*D* indicates the effects on pump activity, which are discussed in more detail in sect. 7.2.

### 4.3. Effects of K_d_ and Ca^2+^ Dependence of Buffering

**FIGURE 10.**
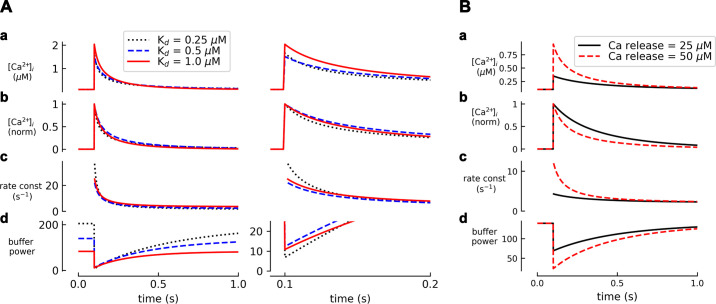
Predicted effects of changing the buffer affinity and amount of Ca^2+^ released on the decay of intracellular calcium concentration ([Ca^2+^]_i_). *A*: effects of *K*_d_. Model similar to that of FIGURE 7 except that the buffer had different *K*_d_ values, as indicated. As for FIGURE 7, the buffer is assumed to bind Ca^2+^ instantaneously. *a*: [Ca^2+^]_i_. *b*: Normalized [Ca^2+^]_i_. *c*: Instantaneous rate constant of decay. *d*: Buffer power. A portion of the records at expanded timescale is displayed on *right*. Note the expanded vertical scale on the *right* graph of *d*. *B*: effects of changing the amount of Ca^2+^ released. The same saturating buffer (*K*_d_ = 500 nM) was used throughout. The increase of total cytoplasmic Ca^2+^ was changed from 25 (black) to 50 (red) µM.

The influence of buffer *K*_d_ on Ca^2+^ signaling can be appreciated from FIGURE 10*A*, which shows a simulation of the effects of adding the same amount of Ca^2+^ to the cytoplasm in the presence of fast buffers with different values of *K*_d_. The effect of buffer *K*_d_ on the decay depends on the fact that (see sects. 3.1. and 3.2) buffer power is sensitive to both *K*_d_ and cytosolic [Ca^2+^]_i_. In contrast to the simulations of FIGURE 7, where buffer power was assumed to be constant (Δ[Ca^2+^]_i_ ≪ *K*_d_), the decays of [Ca^2+^]_i_ are not single exponentials as shown (FIGURE 10*Ab*) by the curves intersecting. This is emphasized in FIGURE 10*Ac*, which plots the instantaneous rate constant of decay of [Ca^2+^]_i_. For a simple exponential decay this would be constant. However, the instantaneous rate constants of all [Ca^2+^]_i_ transients shown are initially high and then decrease. This is caused by the buffer saturation and, consequently, decreased buffer power at high [Ca^2+^]_i_ (FIGURE 10*Ad*). Thus, a given rate of Ca^2+^ pumping results in a faster fall of [Ca^2+^]_i_. Such saturation of Ca^2+^ buffers has been suggested to account for the initial rapid decay of the caffeine-evoked Ca^2+^ transient in ventricular myocytes (197). As evident from the normalized traces (FIGURE 10*Ab*) and especially the instantaneous rate constants, the buffer with the highest affinity (*K*_d_ = 0.25 µM) causes a faster initial decay of the [Ca^2+^]_i_ transients than do the others, because of the greater binding of Ca^2+^ and consequent decrease of buffer power at the peak (FIGURE 10*Ad*). As [Ca^2+^]_i_ approaches resting levels, buffer power of the high-affinity buffer increases and the Ca^2+^ transient decays more slowly. In other words, for a given amount of Ca^2+^ added to the cytoplasm, increasing the affinity of the buffer causes stronger buffer saturation and produces a [Ca^2+^]_i_ decay that crosses over with the lower-affinity cases (see sects. 6.2.2–6.2.5 for other practical consequences in cardiac muscle).

FIGURE 10*B* demonstrates the predictions for increasing the amount of added Ca^2+^. Doubling the Ca^2+^ release into the cytosol results in a larger Ca^2+^ transient (FIGURE 10*Ba*), which initially decays much faster because of buffer saturation (FIGURE 10*Bb*). Once again, it is clear from the plot of instantaneous rate constants (FIGURE 10*Bc*) that the decays are not single exponentials. However, this might not be apparent in real, noisy data, and it would be all too easy to assume that the larger transient decays more quickly because of enhanced Ca^2+^ pumping as opposed to buffer saturation.

### 4.4. The Effects of Cooperative Buffers

**FIGURE 11.**
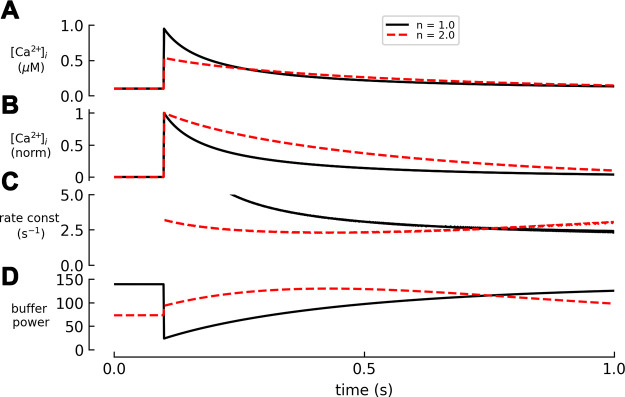
The effect of a cooperative buffer on the decay of intracellular calcium concentration [Ca^2+^]_i_. Model similar to FIGURE 10. The buffer had a *K*_app_ of 0.5 µM. Traces show the predicted effects of values of Hill coefficient (*n*) of 1 (black solid lines) and 2 (red dashed lines). *A*: [Ca^2+^]_i_. *B*: normalized [Ca^2+^]_i_. *C*: instantaneous rate constant of decay. *D*: buffer power.

As shown above, for noncooperative buffers buffer power decreases as [Ca^2+^]_i_ increases. In contrast, for a cooperative buffer, buffer power will first increase but then decrease once [Ca^2+^]_i_ rises above an optimal concentration (FIGURE 5). FIGURE 11 demonstrates that this may have marked effects on Ca^2+^ signaling. The black traces show a simulation (similar to that of FIGURE 10*A*) for a noncooperative buffer (*n* = 1). The red trace shows the prediction for a cooperative buffer (*n* = 2). Here the peak value of [Ca^2+^]_i_ is greater than the level at which maximum buffering occurs, resulting in an increase of buffer power. As [Ca^2+^]_i_ falls toward the level at which peak buffer power occurs, buffer power increases further and this is accompanied by a decrease of the instantaneous rate constant of decay of [Ca^2+^]_i_. However, as [Ca^2+^]_i_ decays below the level of peak buffering, buffer power decreases and the rate constant of decay will increase. Therefore, in contrast to a simple buffer, where for large Ca^2+^ transients the instantaneous rate constant of decay is expected to decrease as [Ca^2+^]_i_ falls, a slowing followed by acceleration should be observed. It should, however, be noted that the kinetics of decay of the Ca transient will also be affected by the dependence of Ca^2+^ pumping on [Ca^2+^]_i._

### 4.5. The Influence of Buffering on the Diffusion of Ca^2+^

Diffusion of Ca^2+^, i.e., the ability of the ion to move along its concentration gradient, is affected by interactions of solvated Ca^2+^ with the components in the medium. A striking physiological example of the ability of Ca^2+^ buffers to enhance diffusion is that of the calbindin proteins, which facilitate Ca^2+^ diffusion across epithelial cell layers (200) (see sect. 10). Often, the bulk of cytoplasmic Ca^2+^ buffering is from fixed structures, i.e., cytoskeleton, membranes, contractile proteins, and pumps on organelles, which will not contribute to the diffusive flux of Ca^2+^ (173, 201). Therefore, even if mobile cytoplasmic Ca^2+^ buffers only make a small contribution to the total cellular buffer power, they can have a large role in diffusion (174, 202–204). The exact contribution will depend on the relative diffusion coefficient of the Ca^2+^ bound forms and on their buffering power (see *Eq. 29*). An estimate for the diffusion coefficient (*D*) can be obtained from the known dependence of *D* on molecular weight (M) as described by a simplified form of the Stokes–Einstein equation (*D* ∝ 1/M^r^); r can be measured empirically and is 0.33–0.40 over a wide range of M (205, 206). In this review, we use a value of 0.33.

Kinetic considerations aside, intracellular Ca^2+^ buffers will affect Ca^2+^ diffusion in two distinct ways: *1*) for any cytosolic influx/efflux event, buffers will reduce the free Ca^2+^ concentration gradient generated and therefore the rate of intracellular diffusion and *2*) the reduction in diffusive flux of Ca^2+^ due to a lower free Ca^2+^ concentration gradient may be offset by a contribution to diffusion by the Ca^2+^-bound form of the buffer; the magnitude of this component will depend on the value of the diffusion coefficient of the complex and the concentration gradient of the bound Ca^2+^ (178, 202, 204).

Specifically, the flux of free Ca^2+^ due to diffusion is described by the Fick equation.

(*21*) $$J_{\mathrm{Ca}}=-D_{\mathrm{Ca}}\cdot \frac{\mathrm{dCa}}{dx}$$

where *D*_Ca_ is the diffusion coefficient of free Ca^2+^ and dCa/d*x* is its concentration gradient.

The flux of bound Ca^2+^ is similarly

(*22*) $$J_{\mathrm{CaB}}=-D_{\mathrm{CaB}}\cdot \frac{\mathrm{dCaB}}{dx}$$

where *D*_CaB_ is the diffusion coefficient of Ca^2+^ bound to buffer and dCaB/d*x* is its concentration gradient.

The total flux is

(*23*) $$J_{\mathrm{Ca}(\mathrm{total})}=J_{\mathrm{Ca}}+J_{\mathrm{CaB}}=-D_{\mathrm{Ca}}\cdot \frac{\mathrm{dCa}}{dx}-D_{\mathrm{CaB}}\cdot \frac{\mathrm{dCaB}}{dx}$$

Henceforth, we assume that Ca^2+^ binding and dissociation are fast enough such that the local bound and free Ca^2+^ are in equilibrium; therefore dCaB/d*x* can be replaced by the product of dCa/d*x* (the free Ca^2+^ gradient) and dCaB/dCa (the buffer power), resulting in

(*24*) $$J_{\mathrm{CaB}}=-D_{\mathrm{CaB}}\cdot \frac{\mathrm{dCa}}{dx}\cdot \frac{\text{dCaB}}{\mathrm{dCa}}$$

which can be rewritten as follows:

(*25*) $$J_{\mathrm{CaB}}=-D_{\mathrm{CaB}}\cdot \frac{\mathrm{dCa}}{dx}\cdot \beta_{i}\left(\mathrm{Ca}\right)$$

Dividing *Eq. 25* by *Eq. 21* gives the relative contribution made by the flux of bound compared to free Ca^2+^ to diffusion as

(*26*) $$\frac{J_{\mathrm{CaB}}}{J_{\mathrm{Ca}}}=\;\frac{D_{\mathrm{CaB}}}{D_{\mathrm{Ca}}}\cdot {\text{β}}_{i}\left(\mathrm{Ca}\right)$$

This means that buffers make the biggest fractional contribution to Ca^2+^ diffusion when they also buffer Ca^2+^ most strongly (β*_i_*_,max_). For a simple noncooperative buffer, where the binding is described by a single dissociation constant (*K*_d_), we can substitute from *Eq. 8* for β*_i_*(Ca) to give

(*27*) $$\frac{J_{\mathrm{CaB}}}{J_{\mathrm{Ca}}}=\frac{D_{\mathrm{CaB}}}{D_{\mathrm{Ca}}}\cdot {\left[B\right]}_{\mathrm{Tot}}\cdot \frac{K_{d}}{{\left(\mathrm{Ca}+K_{d}\right)}^{2}}$$

More generally, including cooperative buffers, we substitute from *Eq. 16*:

(*28*) $$\frac{J_{\mathrm{CaB}}}{J_{\mathrm{Ca}}}=\frac{D_{\mathrm{CaB}}}{D_{\mathrm{Ca}}}\cdot {\left[B\right]}_{\mathrm{Tot}}\cdot \frac{n\cdot K_{\mathrm{app}}^{n}\cdot Ca^{n-1}}{{\left(K_{\mathrm{app}}^{n}+Ca^{n}\right)}^{2}}$$

Hence, noncooperative buffers contribute most to Ca^2+^ diffusion at low [Ca^2+^] (*Eq. 27*). As [Ca^2+^] reaches high levels, the buffer will become saturated [β(Ca) = 0], there will be no gradient of CaB, and therefore no contribution to net diffusion. In contrast, for cooperative buffers, their contribution to Ca^2+^ diffusion will be low at both very low and high [Ca^2+^] and greatest at a value of [Ca^2+^] given by *Eq. 18*. As regards the dependence on *K*_d_, for noncooperative buffers their contribution to diffusion will be largest when *K*_d_ = [Ca^2+^]_i_ (*Eq. 13*) (207), and a similar condition holds for cooperative buffers (*Eq. 20*).

*Equations 21–28* describe the local fluxes contributed by individual Ca^2+^-binding components of the cytosol. When confronted with the problem of solving the spatiotemporal pattern of diffusion of several interacting components, this can, of course, be accomplished by numerical integration (see sect. 4.7). For certain limiting cases, however, simple analytical solutions can be derived, which often provide a more intuitive understanding of the processes involved.

Thus, for the case of small changes in [Ca^2+^] around low values, where the buffering power of all Ca^2+^-binding species is constant, the “apparent diffusion coefficient” for Ca^2+^ (*D*_app_) can be calculated as

(*29*) $$D_{\mathrm{app}}=D_{\mathrm{Ca}}\cdot \frac{1+\sum \left(\kappa_{i}\cdot D_{i}/D_{\mathrm{Ca}}\right)}{1+\sum \kappa_{i}}$$

where the sums extend over all Ca^2+^-binding species (178, 202). The use of κ in *Eq. 29* reflects the original papers, but the equation could equally well be written in terms of β*_i_* (sect. 3).

This equation provides an intuitive understanding in the sense that in the numerator the individual terms of the sum specify contributions of the respective buffers to Ca^2+^ mobility, normalized by the sum of Ca^2+^ binding ratios of all species, irrespective of whether they are mobile or stationary.

TABLE 3 gives values for some important mobile intracellular buffers. The respective relative diffusion constant (*D*_i_/*D*_Ca_), appearing in *Eqs. 26–29*, is given in the table for each buffer. One major contributor to *D*_app_ is ATP, which is present at 5–8 mM in virtually all cell types (34, 203). ATP binds Ca^2+^, Mg^2+^, and H^+^ and, despite its relatively low Ca affinity (*K*_d_ of ∼1 mM), because of its high concentration it can nevertheless bind significant amounts of intracellular Ca^2+^ (sect. 2.4.2). This and its low molecular weight mean that the diffusive flux carried by Ca-ATP will be greater than that due to free Ca^2+^ (see TABLE 3 and *Eq. 27*). This contribution will be larger in cytoplasmic regions close to channels/exchangers where Ca^2+^ influx can increase the local [Ca^2+^] to higher levels, which will saturate higher-affinity buffers. Striated muscle cells also contain high concentrations (up to 20 mM) of histidyl dipeptides (HDPs) such as carnosine (211, 212), and, subject to the concerns about Ca^2+^ binding at physiological pH (sect. 2.4.1), these may also contribute to Ca^2+^ diffusion.

**Table 3.** Predicted effects of various mobile Ca^2+^ buffers on diffusion of Ca^2+^

	Mol Weight, g	Relative Diffusion Constant	Conc, µM	K_app_, µM	Hill Slope	Binding Sites	Ca-Bound, µM	β(Ca)	Relative Flux
Free calcium	40	1.00	0.1					1	1
Calmodulin	16,700	0.14	20^a^	2.7/12.7^b^	1.8/1.9	4	0.05	0.49	0.09
ATP	525	0.43	7,000^c^	2,496^d^	1	1	0.28	2.80	1.20
Carnosine	226	0.56	10,000^e^	1,350^e^	1	1	0.74	7.41	4.18
CB-D9k	10,000	0.16	60^f^	0.21^g^	1.2	2	34.9	148.6	48.0
CB-D28k	28,000	0.12	60^f^	0.31^h^	1.1	4	53.7	114.6	52.8
*EGTA*	380	0.48	100	0.18^i^	1	1	35.7	230	109.2
*Fura-2*	636	0.40	100	0.3^j^	1	1	25.0	188	75.3
*GCAMP*	50,000	0.10	10	0.32^k^	3	4	11.8	86.2	32.8

From *left* to *right*: molecular (atomic) weight (MW); diffusion constant relative to Ca^2+^, calculated as (40/MW)^0.33^; concentration of buffer; *K*_app_; Hill slope; no. of Ca^2+^ binding sites per molecule; concentration of bound Ca^2+^; buffer power (from *Eq. 8* or *16*); diffusion flux relative to that of Ca^2+^, calculated at free Ca^2+^ of 0.1 µM (from *Eq. 27* or *28*). N.B. The concentration for carnosine applies to striated muscle and that for calbindinD9K (CB-D9k) and CalbindinD28K (CB-D28k) to Ca^2+^-transporting epithelia, the concentrations for EGTA, fura-2, and GCAMP represent typical values under experimental conditions. The CaM data represent both the COOH- and NH_2_-terminal sites. All compounds bind 1 Ca^2+^ per molecule except: CaM, 4; CB-D9K, 2; CB-D28K, and 4; GCAMP, 4. The values come from Refs. 208 (a), 187 (b), 34 (c), 33 (d), 49 (e), 209 (f), 186 (g), 30 (h), 24 (i), 25 (j), and 210 (k). Note that the table shows a value of 3 for the Hill coefficient of GCAMP but a range of *K*_d_s is found for different variants from 1 to 3 (210). These values were used to calculate *K*_app_. For noncooperative buffers, this was the apparent *K*_d_ at pH 7.0 and 1 mM Mg^2+^; for cooperative buffers, it was calculated as in TABLE 2. Buffers listed in *italics* are synthetic molecules used in research.

The low intracellular concentration and large molecular weight of Ca^2+^-signaling molecules such as annexin and CaM mean that their contributions to diffusive flux are small (TABLE 3). For CaM, the inferred diffusion coefficient (20–30 µm^2^/s) (203, 204) is approximately what would be anticipated from its molecular weight (∼17,000). The calbindins (CB-D9k and CB-D28k) at nominal cytoplasmic concentrations of 300 µM make a significant contribution to diffusive flux. Interestingly, the lower diffusion coefficient of the larger 28k form is offset by being able to bind four Ca^2+^ compared to the two Ca^2+^ binding sites on the 9k form (see TABLE 3). It is useful to compare the calbindins with CaM. The molecular weights and therefore diffusion constants are of the same order. The much greater contribution to Ca^2+^ diffusion of the calbindins arises from two factors: *1*) The concentration of the calbindins in epithelia is three times greater than that of CaM. *2*) More importantly, the higher affinity of the calbindins means that at lower values of [Ca^2+^]_i_ the buffer power of calbindin, and therefore its contribution to diffusion, is much greater. Finally, the effects of Ca^2+^ indicators to promote intracellular Ca^2+^ diffusion are illustrated by the inclusion of fura-2 and the protein indicator GCaMP in TABLE 3 (note the very high relative fluxes of EGTA and fura-2, which at the assumed concentrations dominate diffusion; see also sect. 4.6).

A mechanism through which a relatively immobile Ca^2+^ buffer can enhance net Ca^2+^ diffusion has been suggested to apply in the case of the intra-SR buffer CSQ (213). CSQ polymers within the SR may mediate enhanced Ca^2+^ diffusion from a distal region of the polymer to a region next to the SR Ca^2+^ channel via the “jumping” of Ca^2+^ ions from binding site to site without going through the aqueous phase, which in a linear polymer would form a “Ca^2+^ wire.” This theory is based on the well-known physical process of diffusion on an adsorptive surface (214) and may enhance net diffusion by 10- to 100-fold. There is, however, no direct evidence that this process features in intra-SR Ca^2+^ diffusion.

#### 4.5.1. Linkage between diffusion of calcium and protons.

Buffer-mediated diffusion of Ca^2+^ may also be linked to that of protons, since many buffers can bind both ions. This was originally proposed for calbindin-mediated transport, where it was suggested that protons might facilitate Ca^2+^ binding resulting in a coupled cotransport of Ca^2+^ and protons (207). However, there is no evidence to suggest that protons increase Ca^2+^ binding, and, indeed, the opposite is more likely, as ∼1 H^+^ is released when Ca^2+^ binds to an EF-hand domain (see sect. 2.6.1.2). It is therefore likely that calbindin-mediated Ca^2+^-facilitated diffusion results in countertransport of protons. More recently, competition of H^+^ and Ca^2+^ for binding to ATP and carnosine has been suggested to produce a countertransport of Ca^2+^ and protons in cardiac cells. Acidifying a region of a cell resulted in an increase of [Ca^2+^]_i_ in that region. This was explained by the buffers shuttling protons away from this region and returning with Ca^2+^ bound (49).

### 4.6. Effects of Exogenous Buffers on Ca^2+^ Signaling

The use of small-molecule intracellular Ca^2+^ indicators will increase Ca^2+^ buffering and thus decrease the amplitude of [Ca^2+^]_i_ transients and alter their kinetics (174, 215) (sect. 4.1). The magnitude of the effect will depend on both the concentration and *K*_d_ of the indicator. An important requirement for [Ca^2+^]_i_ fluorimetry, therefore, is to keep the concentration of indicators low, preferably much lower than that of endogenous buffers. The low molecular weight of fura-2 and its high buffer power at low [Ca^2+^]_i_ (as a consequence of its high affinity) require extremely low concentrations of this indicator for unperturbed measurement of [Ca^2+^]_i_ signals (204). The low *K*_d_ means that this problem lessens at higher [Ca^2+^]_i_ as buffer power falls. Equally, however, the accuracy of measuring [Ca^2+^]_i_ falls at higher [Ca^2+^]_i_ as the indicator saturates. If the indicator is loaded via a patch pipette, then its concentration is known. Most studies, however, use the membrane-permeant acetoxymethyl (AM) ester form, and the cytosolic concentration is therefore unknown. TABLE 3 shows the effects of a typical 100 µM dye concentration. At 0.1 µM [Ca^2+^]_i_, there is a significant contribution to buffer power. Lower-affinity indicators will produce less buffering. However, as [Ca^2+^]_i_ increases, the contribution to buffering of low-affinity indicators will be greater than that of high-affinity indicators (FIGURE 3).

Exogenously applied slow buffers have been used for a variety of purposes in physiological experiments. In muscle, Ca^2+^ release from the SR has been studied as Ca^2+^ “spikes” by using a fast fluorescent Ca^2+^ indicator in combination with a higher concentration of the slow Ca^2+^ buffer EGTA (198). The slow buffer keeps [Ca^2+^]_i_ low so the fast indicator will be mainly in the low-fluorescence state. When Ca^2+^ is released, [Ca^2+^]_i_ will briefly and locally reach high levels and thereby be detected by the fluorescent indicator before Ca^2+^ is bound to EGTA. The amplitude of the spike of [Ca^2+^]_i_ then gives a measure of the release flux (198, 216, 217). Similarly, so-called “puffs” of local elevated [Ca^2+^]_i_, released from the endoplasmic reticulum by IP_3_, have been studied in various cell types (218–220). Local domains of elevated [Ca^2+^] in the vicinity of Ca^2+^ channel clusters were analyzed at ribbon synapses in auditory hair cells by both Ca^2+^ imaging and computational approaches (221–223). Ca^2+^ signals due to the opening of single voltage-gated Ca^2+^ channels could be resolved by total internal reflection fluorescence (TIRF) microscopy (224). A careful balance of fast low-affinity indicator dyes and slow high-affinity buffers (usually EGTA) is required for optimum resolution (see sect. 4.7.2 for a detailed discussion of local [Ca^2+^]_i_ domains).

The buffering effect of Ca^2+^ indicators can also be used to advantage. In the extreme case of a large excess of indicator, changes in [Ca^2+^]_i_ will be very small, but since the indicator binds essentially all the Ca^2+^ changes in fluorescence at the Ca^2+^-dependent wavelength of an indicator dye will be proportional to fluxes and can be used for quantitative determination of Ca^2+^ fluxes through ion channels (173, 225–227).

Exogenous buffers will also affect Ca^2+^ diffusion. As mentioned above, mobile endogenous buffers contribute only ∼20% of the total cytoplasmic buffer power, so added Ca^2+^ indicators will increase the relative concentration of mobile buffers within the cytosol and therefore alter physiological Ca^2+^ gradients and fluxes (174, 203, 204). Although generally present at concentrations (∼50 µM) lower than those of endogenous buffers, these indicators, mostly derivatives of BAPTA, have relatively high affinity (*K*_d_ 0.3–1 µM) and therefore their contribution to buffer power and thence Ca^2+^ diffusion may increase to levels that match or exceed that of endogenous mobile buffers, thereby increasing the diffusion of Ca^2+^ and attenuating intracellular Ca^2+^ gradients. This is analogous to the very large influence of low-molecular weight metabolites such as ATP (sect. 2.4.2) on *D*_app_ of Ca^2+^ (*Eq. 29*) in cells that contain only low levels of endogenous mobile CBPs. Therefore, and as indicated in TABLE 3, only by ensuring low cytoplasmic indicator concentrations (<10 µM) or using indicators with a low diffusion coefficient can experimenters hope to preserve physiological intracellular Ca^2+^ gradients while measuring intracellular Ca^2+^ signals with high-affinity Ca^2+^ dyes. Genetically encoded Ca^2+^ indicators of the GCaMP family (228), which are derivatives of GFP, have a MW of ∼ 50,000 and therefore diffuse considerably more slowly (TABLE 3). Nevertheless, at least at resting [Ca^2+^]_i_, they are predicted to make a significant contribution to Ca^2+^ buffering and to interfere with physiological processes (215, 229). In practice, there is a compromise between avoiding excessive additional mobile buffer, while having adequate signal to noise. Efforts are being made to develop more sensitive and brighter protein-based probes (230, 231), which, because of their high MW, may preserve the endogenous diffusion properties better than conventional Ca^2+^ indicators. An alternative approach is to use dextran-coupled dyes (220, 232). Furthermore, Ca^2+^ indicators can be targeted to structures within the cell (233, 234), thereby becoming immobile. However, as immobile buffers they may still retard Ca^2+^ diffusion according to studies of diffusion in cytoplasm (235) because of their contribution in the denominator of *Eq. 29*.

Studies on the influence of Ca^2+^-indicator dyes and other small Ca^2+^-binding molecules on *D*_app_ of Ca^2+^ have shown that this very much depends on the presence of endogenous buffers. With a MW of ∼400, the diffusion coefficient of indicators would be ∼50% of that of free Ca^2+^ (200–220 µm^2^/s). Thus, one would expect *D*_app_ to be near 100 µm^2^/s if the indicator dye were the dominating buffer. If, on the other hand, an immobile low-affinity buffer with a buffering power of ∼20 (236) were present as the only buffer, *D*_app_ would be expected to be around 200/20 = 10 µm^2^/s. Measured or inferred values are often in between these values. In rod photoreceptor cells, for instance, *D*_app_ was found to be only ∼15 µm^2^/s (236), pointing toward a small contribution of the mobile indicator or else a lower buffering power of the fixed buffer. A detailed Ca^2+^ imaging and modeling study on atrial myocytes (203) concluded that because of the presence of immobile buffers the apparent diffusion coefficient would be as low as 4.25 μm^2^/s if diffusion were not accelerated by the presence of indicator dyes and ATP. An imaging study of Ca^2+^ diffusion within nerve axons (204) measured *D*_app_ for a range of different indicator concentrations and extrapolated to zero indicator. This yielded an upper bound for D_app_ of <16 µm^2^/s (see also sect. 4.7.3). Estimates of *D*_app_ in skeletal muscle (237) as well as cardiac muscle (238) were in a similar range of 15–30 µm^2^/s. Such low values are only obtained, however, if care is taken to use as low indicator concentrations as possible, unless endogenous buffers dominate. Otherwise, the relatively mobile indicators speed up Ca^2+^ diffusion. The study of Gabso et al. (204), for instance, concluded that *D*_app_ is increased by more than a factor of 3 by 100 µM fura-2. In contrast, the low-affinity indicator CaGreen 5N increases *D*_app_ by only twofold when used at even twice the concentration (200 µM). This was calculated for the case of nerve axons of cultured *Aplysia* neurons, which have a low abundance of endogenous buffers. Higher concentrations of endogenous mobile and immobile buffers reduce the influence of Ca^2+^-indicator dyes on *D*_app_. Furthermore, immobile buffers may be heterogeneously distributed in subregions of the cell cytoplasm in some of which large Ca^2+^ gradients are generated. A good example is in muscle, where the major Ca^2+^ buffer, TnC, is only found in the myofilament compartment.

### 4.7. Modeling of Spatiotemporal Gradients

Quantitative solutions to the combined problem of binding of Ca^2+^ to several buffers and diffusion in a realistic geometry can only be obtained by numerical computation. Early work by Roberts (239) on the [Ca^2+^]_i_ patterns generated by Ca^2+^ influx through arrays of Ca^2+^ channels in saccular hair cells provided important insights into the interaction between Ca^2+^ channels and Ca^2+^-activated K^+^ channels. This sparked major efforts for the development of software platforms to handle such calculations. Both stochastic tools such as MCell (https://mcell.org/) (240) and deterministic approaches, e.g., CalC (https://web.njit.edu/~matveev/calc.html) (241) have become available. These tools and custom-made software have been used to simulate signals generated by arrays of voltage-dependent Ca^2+^ channels (222, 242–244) and so-called Ca^2+^ sparks or Ca^2+^ puffs (218–220, 245–247). Such calculations provide a complete picture of the complex features, which arise by the combination of buffering and diffusion in a specific geometric environment. They may include the kinetics of Ca^2+^ binding to and dissociation from several ligands. However, images and graphs produced by these software tools are often snapshots that convey limited intuitive understanding. Therefore, it is worthwhile considering approximate solutions to the problem, which are valid for certain limiting scenarios (178). One of these, the so-called “single compartment model” (SCM) considers the case that the structure of interest is small enough that diffusional equilibration in a given compartment is fast on the timescale of interest. Thus, the SCM considers homogeneous concentrations throughout, and mobility of buffers is not an issue. This assumption was made for many of the equations and features discussed so far.

Another limiting case is the so-called “rapid buffer approximation” (RBA), which considers timescales that are long enough that all binding/dissociation reactions are at equilibrium, while there may still be diffusional gradients within the structure of interest. Equation *29*, which allows one to calculate an apparent diffusion coefficient, *D*_app_, is an example for the rapid buffer approximation. With the additional assumption of linearity of the buffer, the so-called “linear buffer approximation” (LBA), predictions such as those by *Eq. 29* become very simple but are restricted to [Ca^2+^]_i_ smaller than the *K*_d_ of the ligand with the highest affinity.

#### 4.7.1. Approximations for special cases.

Here we describe the assumptions and the use of approximate calculations for the special cases listed above.

##### 4.7.1.1. the single compartment model.

For small structures on the order of a few micrometers, such as small cell bodies and longitudinally homogeneous dendrites, [Ca^2+^] gradients equilibrate on the timescale of 20 ms according to the Stokes–Einstein equation, assuming *D*_app_ = 200 µm^2^/s. The single compartment model (SCM) is typically applied to describe time courses of small global [Ca^2+^]_i_ transients elicited by action potentials (APs) in presynaptic boutons, dendritic spines, small cell bodies of neurons, and endocrine cells, such as adrenal chromaffin cells. Although the latter have diameters up to 15 µm, a detailed quantitative analysis of [Ca^2+^]_i_ gradients found that such gradients elicited by short episodes of Ca^2+^ influx largely dissipated within 25 ms (163). The SCM is also applicable to striated muscle cells made up of many identical sarcomeres, each of which can be modeled as a single compartment. Together with the assumption of linearity and rapid equilibration (see the LBA and RBA discussed below), the SCM predicts that [Ca^2+^]_i_ transients have a rapid rise with an amplitude proportional to the total amount of Ca^2+^ influx and an exponential decay according to

(*30*) $${\left[Ca^{2+}\right]}_{i}\left(t\right)=\text{Δ}{\left[Ca^{2+}\right]}_{i}\mathrm{\cdot exp}\left(-t/\tau_{\mathrm{Ca}}\right)$$

with

(*31*) $$\text{Δ}{\left[Ca^{2+}\right]}_{i}=\frac{q_{\mathrm{Ca}}}{2F\cdot v\cdot (1+\sum \kappa_{i})}$$

where *q*_Ca_ is the charge carried by the Ca^2+^ influx, *F* is the Faraday constant, and v is the accessible volume of the compartment (174). The sum of the denominator in *Eq. 31* extends over all Ca^2+^ buffers. The time constant τ_Ca_ is given by

(*32*) $$\tau_{\mathrm{Ca}}=\frac{1+\sum \kappa_{i}}{\gamma }$$

with γ representing the relationship between the Ca^2+^ extrusion (pump) mechanism and [Ca^2+^]_i_ (Refs. 174, 248; see also a graphical representation of these equations in FIGURE 3). It is readily seen that the time integral of [Ca^2+^]_i_(*t*), the product of amplitude Δ[Ca^2+^]_i_ and time constant τ_Ca_, is independent of buffering (*q*_Ca_/(2*F*·v·γ).

##### 4.7.1.2. the rapid buffer approximation.

Most Ca^2+^ buffers of interest have relatively fast binding kinetics (sect. 2.1), with the exception of PV, a CBP with high expression in skeletal muscle and certain neuron types (sects. 7.2 and 9.2), and the exogenous buffers EGTA and EDTA. For studying cellular properties on the millisecond to second timescale, it is, therefore, often convenient to assume that [Ca^2+^]_i_ is at equilibrium with all buffers (201, 249–254). This approximation misses, of course, some very interesting kinetic features caused by slow buffers, but it simplifies computations enormously (178). In particular in combination with the LBA, discussed below, some important features of buffering and diffusion can be understood intuitively on the basis of very simple equations, such as *Eq. 30–32*. But the rapid buffer approximation (RBA) can also be extended by including buffer saturation, which expands its range of applications (202). Furthermore, extensions for the case of buffers with two binding sites have been described (188, 241).

##### 4.7.1.3. the linear buffer approximation.

As stated by *Eq. 8*, the buffer power β of a given ligand with dissociation constant *K*_d_ is nearly constant for [Ca^2+^]_i_ ≪ *K*_d_. For small [Ca^2+^]_i_ transients around resting values and in the range of a few hundred nanomolar, neglecting buffer saturation may be acceptable and proves helpful in understanding the changes in [Ca^2+^]_i_ dynamics induced by the presence of buffers. This implies that changes in [Ca^2+^]_i_ are proportional to changes in total [Ca^2+^] (178, 255–257). Furthermore, Ca^2+^ signals caused by various processes add linearly—as long as the resulting summed response stays well below the *K*_d_ of the buffer with the highest affinity. Exploiting these properties, the aggregate signal and expected neurotransmitter release has been calculated for various arrays of Ca^2+^ channels, which becomes particularly simple in the presence of millimolar concentrations of EGTA (Refs. 255, 256, 258, 259; see also sect. 4.7.2 on local domains). As noted above, the linear buffer approximation (LBA) is reasonable for the small changes of [Ca^2+^]_i_ that typically occur in neurons. For example, from *Eq. 8*, assuming a buffer *K*_d_ of 0.75 µM, the buffer power at a peak [Ca^2+^] of 200 nM will be 71% of that at 50 nM resting [Ca^2+^]_i_. In contrast, with the same *K*_d_, the buffer power at the very much higher peak of a cardiac Ca^2+^ transient (1 µM) will be only 24% of that at a resting [Ca^2+^]_i_ of 100 nM, and the approximation would be problematic (see also FIGURE 3).

The LBA, together with the RBA, leads to simple equations like *Eq. 29* for the apparent diffusion coefficient. It was used to describe the diffusional spread in cylindrical (178, 204) and spherical (163) structures. Together with RBA and applied to a single compartment, it leads to a simple description (*Eqs. 30–32*) of exponentially decaying small [Ca^2+^]_i_ transients, as frequently observed in neurons.

#### 4.7.2. Local domains.

Many Ca^2+^-dependent processes are triggered very locally by single-molecule events. Prominent examples are the release of neurotransmitters upon the opening of one or only few voltage-dependent Ca^2+^ channels and the generation of “sparks” of [Ca^2+^]_i_ in muscle due to opening of RyR-operated channels in the SR. Numerical simulations have shown that such local domains, often called “microdomains” or “nanodomains,” of elevated [Ca^2+^]_i_ rise within microseconds, stay elevated as long as Ca^2+^ channels are open, and decay equally rapidly after channel closure (178, 239, 246, 256, 257, 260–266). Mobile buffers are particularly efficient in shaping such local domains, since diffusion across the small dimensions of these domains is very rapid, such that when buffer binds Ca^2+^ it is rapidly replaced by free buffer. A particularly simple scenario emerges in the presence of slow buffers, such as EGTA (256, 262, 263) or PV (267). Because of their slow Ca^2+^ binding and rapid diffusional replacement they can “penetrate” the local domain without significant changes in their free concentration, [B]_0_. Where [B]_0_ is high enough, such that its local value does not change appreciably with respect to the bulk [B]_0_, the buffer constitutes a spatially and temporally uniform sink, leading to a differential equation with a particularly simple solution for Δ[Ca^2+^](*r*), the steady-state increment in [Ca^2+^]_i_ at distance *r* from the channel mouth (262):

(*33*) $$\text{Δ}\left[Ca^{2+}\right](r)=\frac{i_{\mathrm{Ca}}}{4\pi FrD_{\mathrm{Ca}}}\cdot \exp\left(-r/\lambda \right)$$

where *i*_Ca_ is the single-channel calcium current, *F* is the Faraday constant, and λ is the length constant of the domain, which is given by

(*34*) $$\lambda =\sqrt{D_{\mathrm{Ca}}/\left(k_{\mathrm{on}}\cdot {\left[B\right]}_{0}\right)}$$

Here, k_on_ is the apparent Ca^2+^ binding rate constant of the buffer (178, 262). The time constant, τ, of rise and decay of the local domain is given by

(*35*) $$\tau =1/\left(k_{\mathrm{on}}\cdot {\left[B\right]}_{0}\right)$$

These equations hold for the case when the slow buffer is the dominating one. They do not consider stationary buffers. In the presence of stationary, very fast-binding low-affinity buffers, for which the RBA and the LBA apply, a steady-state solution for free [Ca^2+^]_i_ very similar to that in the absence of stationary buffers is obtained. The characteristic time constant, however, has to be increased by a factor of 1 + κ_s_ (268), where κ_s_ is the sum of values for all such stationary buffers. It should be noted that in general diffusion is slowed down in the presence of stationary buffers. Nevertheless, the diffusion coefficient appearing in *Eqs. 33* and *34* is that of free Ca^2+^, since the equations describe the steady state for which fixed buffers are in equilibrium with free Ca^2+^. Therefore, the rates that represent binding and dissociation of Ca^2+^ to/from fixed buffers in the respective differential equations cancel each other. Values for λ and τ × (κ_s_ + 1) for various concentrations of EGTA and PV, assuming κ_s_ = 21, are given in TABLE 4 (248). These are meant to provide an “order-of-magnitude” idea about the extent and dynamics of local domains in the presence of a dominating slow buffer.

**Table 4.** The dependence of length constant and time constant of local [Ca^2+^] domains

Buffer Conc, mM	λ_EGTA,_ µm	τ_EGTA_, ms	λ_PV_, µm	τ_PV_, ms
1	0.22	5.02	0.27	7.10
2	0.16	2.51	0.19	3.55
5	0.10	1.00	0.12	1.42
10	0.07	0.50	0.084	0.71
20	0.05	0.25	0.060	0.35

Length constant (λ) and time constant (τ) for EGTA and parvalbumin (PV) were calculated, using *Eqs. 34* and *35* under the following assumptions: diffusion coefficient of free Ca^2+^ (*D*_Ca_) = 220 µm^2^/s; apparent association rate constants k_on,app_ = 4.38 µM^−1^·s^−1^ for EGTA (248) and k_on,app_ = 3.1 µM^−1^·s^−1^ for PV at a Mg^2+^ concentration of 1 mM. For the calculation of k_on,app_, we assumed 1 binding site per molecule to be relevant for the buildup and decay of the local intracellular calcium concentration ([Ca^2+^]_i_) domains with k_on_ = 103 µM^−1^·s^−1^ (27). Time constants were further multiplied by a factor of 22 to include the effects of a fast, immobile buffer with a buffering power of 21 (248).

Equation *33* provides intuitive insight: The term $\frac{i_{\mathrm{Ca}}}{4\pi FrD_{\mathrm{Ca}}}$ represents the solution in the absence of buffers and reflects diffusion of Ca^2+^ from a point source in homogeneous medium. The effect of the slow buffer at steady state is exclusively contained in the length constant, where it appears as the so-called “buffer product” *k*_on_ × [*B*]_0_. The diffusion coefficient of the slow buffer does not appear in these equations, since it is assumed that diffusion is fast enough to secure a constant concentration of free buffer within the domain. These approximations do not depend on the RBA and LBA but, importantly, on the assumption regarding nondepletion of free slow buffer. Clearly, the slow buffer is not at equilibrium with [Ca^2+^]_i_ within that domain. Nevertheless, local domains in the presence of sufficiently high concentrations of free slow buffer superimpose linearly as long as their combined Ca^2+^ load does not deplete free buffer (see also sect. 9.4).

The validity of these conclusions about local domains has been confirmed by solving the underlying equations analytically (269) and by numerical computation (270). In these calculations, several buffer species were included and it was shown that very close to the site of entry Ca^2+^ ions are first captured by the buffer with the highest buffer product (*k*_on_ × [*B*]_0_). This was ATP, which was assumed to be present at a free concentration of 0.17 mM. However, as a low-affinity buffer, ATP rapidly releases Ca^2+^ again. During cycles of binding and unbinding from ATP, buffers with lower buffer product can capture Ca^2+^. If they are of higher affinity, they will retain the Ca^2+^ ions for longer times, while moving away from the Ca^2+^ source. In the end, the buffer species with the highest affinity will carry the bulk of Ca^2+^, according to equilibrium conditions. The resulting spatial profile of the local domain decays multiexponentially, unless the buffer with the highest affinity also has the highest buffer product. Numbers given in TABLE 4 assume that this is the case for EGTA and PV, respectively.

The influence of small organic molecules with low affinity but higher buffer product on the properties of local domains has largely been neglected in the literature so far, despite their important role in shaping these domains and likely consequences for physiological processes (see sect. 9.4 and Ref. 60 on the effect of gluconate on neurotransmitter release).

#### 4.7.3. Diffusion in a cylinder.

Elongated cells or subcellular compartments such as axons, dendrites, cardiac myocytes, and muscle fibers often have narrow diameters such that diffusional gradients along their cross section are small because of rapid equilibration. However, gradients may be very pronounced longitudinally. In this case, the spread of Ca^2+^ along the longitudinal axis can be conveniently described by simple analytical expressions, Additionally, if both rapid buffer approximation (RBA) and linearity of buffers (LBA) are assumed, the transport of Ca^2+^ along the cylinder will follow the classical one-dimensional diffusion equation with *D*_app_ according to *Eq. 29* (178, 202, 204). If a leak of Ca^2+^ across the cylinder wall is considered, the same simplifying assumptions (RBA and LBA) lead to an equation analogous to that for propagation of voltage in a leaky cable (268). Using this simplified formalism, Gabso et al. (204) attempted to measure *D*_app_ in largely intact axons of the snail *Aplysia*. They locally injected, via a microelectrode, boluses of Ca^2+^ into uniform lengths of axons, which had previously been loaded with defined concentrations of indicator dyes. Analyzing images of Ca^2+^ spread as a function of position and time after injection, they found that the local [Ca^2+^]_i_ elevation had a Gaussian shape, which spread and decayed rapidly. According to theory, the square of the half-width of that Gaussian should be proportional to the time since the Ca^2+^ injection and the apparent diffusion coefficient, *D*_app_. This allowed evaluation of buffering power and *D*_app_ for various concentrations of the indicator dye. To obtain estimates for the case of an unperturbed cell, the results were extrapolated to zero indicator dye. This yielded values for *D*_app_ (<16 µm^2^/s) and for κ_s_ (<60). Unfortunately, only upper bounds could be provided in this analysis, since the extrapolation depended very much on the measurements with the lowest indicator concentrations, which were very noisy. The finding that *D*_app_ is much smaller than *D*_Ca_ points toward the presence of fixed buffers that retard diffusion. On the other hand, the upper bound (16 µm^2^/s) is higher than the lowest possible value, *D*_Ca_/(κ_s_ + 1) = 223 µm^2^/s/61 ≈ 3.7 µm^2^/s, which would be expected if all endogenous buffers were immobile. Thus, it can be concluded that mobile buffers must also be present.

In the light of the above considerations, we now turn to the role of buffers in specific instances of physiology and pathology. Most of the quantitative work has been carried out in nerve and muscle, but, although there are tissue-specific differences in buffering, the general principles will apply throughout.

## 5. THE RED BLOOD CELL

Red blood cells differ from all other cell types in that their total Ca^2+^ content is unmeasurably low. In human red blood cells the Ca^2+^ content extractable by ionophores and chelator treatments amounts to <1 μmol/(L packed cells) (271). Most mammalian red blood cells contain no organelles, making it simpler to measure their cytoplasmic buffering than is the case in many other cell types. An early study measured Ca^2+^ binding at relatively high levels of Ca^2+^ (20 µM to 1.4 mM) in suspensions of freeze-thawed red blood cells. A *K*_d_ of ∼0.3 mM was reported (272). Subsequent work measured Ca^2+^ buffering in lysed red blood cells (thereby preserving any contribution from cellular contents) and found a linear dependence of bound on free Ca^2+^, over the range 0.1 to 3 mM, with bound and free Ca^2+^ being approximately equal. When the cell constituents were removed by dialysis, all the bound Ca^2+^ was lost, suggesting that cell membranes do not contribute appreciably to buffering (273). Because these studies were performed at Ca^2+^ levels much greater than normal cytoplasmic, higher-affinity buffers would have been saturated and their contribution would have been overlooked.

Buffering was measured in intact red blood cells by controlling intracellular free Ca^2+^ with an ionophore (A23187) while measuring total intracellular Ca^2+^ with ^45^Ca (274). Again, total Ca^2+^ was proportional to free with a buffer power of only 2–3. A subsequent study (275) measured buffering at [Ca^2+^]_i_ levels as low as 100 nM and again found a linear (nonsaturable) binding, attributed to binding to hemoglobin and other proteins, with bound Ca^2+^ equal to ∼35% of free, a very low buffer power. In addition, there was evidence of a saturable buffer with *K*_d_ of ∼8 µM and maximum capacity of ∼100 µM, thereby contributing a buffer power of ∼12 at 100 nM [Ca^2+^]_i_. A tentative suggestion for the identity of this component was calpromotin, now known as Peroxiredoxin 2, a major antioxidant (276). CaM was also considered, but its concentration is too low to account for more than a small fraction. It should, however, be noted that the saturable buffer was identified by subtracting the nonsaturable component from the total, and, as pointed out by the authors, this limited the precision of the estimation. Importantly, no evidence was found for high-affinity buffers with *K*_d_ values below 1 µM. This contrasts with the tissues reviewed in subsequent sections, where the major buffer power is provided by buffers with such low *K*_d_s.

The low Ca^2+^ buffering of the red blood cell has been suggested to be physiologically important as it means that small Ca^2+^ fluxes can produce large changes of [Ca^2+^]_i_. Given the very limited metabolic reserves of this cell, minimizing these fluxes, and the consequent demand for ATP to pump Ca^2+^ out of the cell, is important for the economy of the cell. Human red cells have a programmed circulatory life span of ∼120 days (277). As the cells deform when traversing capillaries, the mechanosensitive PIEZO1 channels of the cell membrane (278, 279) become transiently activated, allowing brief episodes of Ca^2+^ influx. The low calcium buffering power of the cells allows this minimal Ca^2+^ influx to elevate [Ca^2+^]_i_ sufficiently to activate Ca^2+^-sensitive K^+^ channels, leading to progressive KCl and water loss. This explains the physiological mechanism behind the progressive increase in the density of aging red cells in the circulation and highlights the important role of low Ca^2+^ buffering by minimizing the magnitude and maximizing the speed of the leak-restorative Ca^2+^ fluxes by the Ca^2+^ pump during each capillary transit (280–282).

The low buffer power of the red blood cell contrasts with the much higher values (by a factor of 10–100 fold) in the cells and tissues considered in subsequent sections. A major aim of these sections is to identify the substances responsible for this difference of buffering and the physiological roles of the greater buffering.

## 6. CARDIAC MUSCLE

Each heartbeat is activated by a systolic increase of [Ca^2+^]_i_, derived from Ca^2+^ entry via the L-type Ca^2+^ channel and by Ca^2+^ release from the sarcoplasmic reticulum through the ryanodine receptor (RyR). The amplitude of this free Ca^2+^ transient is of the order of 1 µM and results from an increase of total cytoplasmic [Ca^2+^] of ∼100 µM. Changes of the size of the Ca^2+^ transient are the major factor regulating contraction strength (see Ref. 81 for review). For the heart to work as a pump it must also relax during diastole; Ca^2+^ is lowered back to resting levels via a combination of uptake back into the SR via SERCA and pumping out of the cell (largely via Na/Ca exchange, NCX). Ca^2+^ buffers will therefore affect both the amplitude and rate of decay of the Ca^2+^ transient.

### 6.1. Measurements of Buffer Power in Cardiac Muscle

There are many direct measurements of Ca^2+^ buffering in intact myocytes that can be compared with the measured concentrations of potential buffers. Initial estimates were obtained from Ca^2+^ titration of isolated myofilaments indicating that these alone could contribute a buffering power of at least 10 (283). Similar studies on cardiac homogenates gave a value of 70 (284), and work on permeabilized myocytes showed that cytoplasmic Ca^2+^ buffering could be represented by a buffer with a *K*_d_ of 0.42 µM at a concentration of 78 µM as well as a lower-affinity component (285). The higher-affinity component would contribute a buffer power of ∼120 at 0.1 µM [Ca^2+^]_i_. Subsequent studies have measured cytoplasmic buffering in intact cells by comparing changes of [Ca^2+^]_i_ measured with a fluorescent indicator to those of total Ca^2+^ calculated from the integral of a membrane current. One approach uses the L-type Ca^2+^ current activated by depolarizing pulses (286, 287). A related technique applies caffeine to release Ca^2+^ from the SR. The Ca^2+^ is then pumped out of the cell largely by the electrogenic Na/Ca exchange (NCX), and integrating the NCX current gives a measure of the change of total Ca^2+^ (254). This method requires correction for that fraction of Ca^2+^ that is transported by the electroneutral PMCA. In rat ventricular myocytes, the L-type Ca^2+^ current method provided a *K*_d_ of 0.96 µM and a B_max_ of 123 µM (287) whereas the NCX method gave 0.49 µM and 149 µM, respectively. Various sources of errors need to be considered when comparing these values. First, the cytoplasmic volume must be measured to convert the measured total change of Ca^2+^ to a concentration. Second, the accuracy depends on that of the measurement of [Ca^2+^]_i_. There are at least two potential problems: *1*) Calibrating the measured fluorescence signals in terms of absolute [Ca^2+^]_i_ requires knowledge of the minimum and maximum fluorescence as well as the *K*_d_ of the indicator dye (36), and these values can be affected by the cytoplasmic environment (288–292). Although this is a particular problem for single-wavelength indicators such as the Fluo family, it is not trivial even for ratiometric indicators. Indeed, the Fluo indicators have the advantage that fluorescence is essentially zero in the absence of Ca^2+^, meaning that calibration only requires knowledge of the maximum fluorescence and *K*_d_. The literature shows a range of values assumed for even the most used indicators (288–292). *2*) Accurate calculation of the *K*_d_ of the buffer requires measurements over a wide range of [Ca^2+^]_i_, certainly much greater than the *K*_d_ value. That this is not always possible complicates, for example, establishing whether buffering is cooperative. Indeed, many cardiac buffer curves can be fit reasonably well by a linear regression. It would also be useful to make measurements at values of [Ca^2+^]_i_ below the normal resting level. These issues should not, however, affect measurement of the buffer power over the range of [Ca^2+^]_i_ studied, which implies that such measurements are probably most useful for comparing buffering between two conditions. Absolute values need to be treated with caution, a problem when trying to compare with the measured concentrations of cellular CBPs.

[Ca^2+^]_i_ in the bulk cytoplasm in cardiac muscle is generally in the range 0.1 to 1 µM. The major buffers in cardiac muscle are listed in TABLE 5. This follows from previous compilations (81, 294). For simplicity, we ignore the modest contributions from the sarcolemmal binding sites. The top five rows of TABLE 5 show the faster buffers and the lower two rows the slower (see sect. 6.1.3). At 0.1 µM [Ca^2+^]_i_, the total buffer power provided by the faster buffers is predicted to be ∼140, in reasonable agreement with the experimental values reviewed above. Importantly, the bulk of the buffering is provided by immobile buffers, with the mobile buffers (calmodulin, carnosine, and ATP) contributing <10%.

**Table 5.** Contributions of various compounds to calcium buffering in cardiac muscle

Buffer	Conc, µM	K_app_, µM	Hill Slope	Binding Sites	β*_i_*(Ca) 0.1 μM (ss)	β*_i_*(Ca) 0.2 μM (ss)	β*_i_*(Ca) 1.0 μM (ss)	β*_i_*(Ca) 10 μM (ss)	β*_i_*(ΔCa) 0.1–1.0 μM (in)	β*_i_*(ΔCa) 0.1–1.0 μM (ss)	β*_i_*(ΔCa) 0.1–10 μM (in)	β*_i_*(ΔCa) 0.1–10 μM (ss)	Ref.
Calmodulin	6	2.7/12.7	1.8/1.9	4	0.6	1.0	2.8	0.7	2.0	2.0	1.6	1.6	187
SERCA	48	0.60	1.9	2	56.8	89.5	36.3	0.1	73.9	73.9	9.3	9.3	98
TnC (reg)	70	0.60	1	1	85.7	65.6	16.4	0.4	37.5	37.5	5.7	5.7	78
Carnosine	10,000	1,350.00	1	1	7.4	7.4	7.4	7.3	7.4	7.4	7.3	7.4	49
ATP	5,000	2,496	1	1	2.0	2.0	2.0	2.0	2.0	2.0	1.9	2.0	33
Myosin	70	9.10	1	2	15.1	14.7	12.5	3.5	0.5	13.7	0.1	7.3	78
TnC (nonspec)	70	0.10	1	2	349.9	157.2	11.9	0.1	2.3	64.5	0.2	7.0	78
Total					517	338	89	14	126	201	26	40	

From *left* to *right*: cytoplasmic concentration; *K*_app_; Hill slope (*n*); no. of binding sites; steady-state (ss) buffer power (β*_i_*) at 0.1, 0.2, 1.0, and 10 µM Ca^2+^, calculated with *Eq. 8* or *16*; and the buffer power for a change in calcium concentration ([Ca^2+^]) [β(ΔCa)] for 0.1–1 µM and 0.1–10 μM Ca^2+^, calculated from the change in Ca^2+^ binding (*Eq. 5* or *15*). To indicate the relative contributions of the fast and slow buffers, we show both instantaneous buffer power (in, assuming equilibration of Ca^2+^ binding but no change in the Mg^2+^ binding state) and steady-state buffer power (ss, assuming equilibrium at both Ca^2+^ and Mg^2+^ sites). The value of the Hill slope for the regulatory Troponin-C (TnC) site is taken from isolated TnC (78) and ignores the influence of possible cross-bridge interactions (sect. 3.3).SERCA, sarco(endo)plasmic reticulum Ca^2+^-ATPase.

**FIGURE 12.**
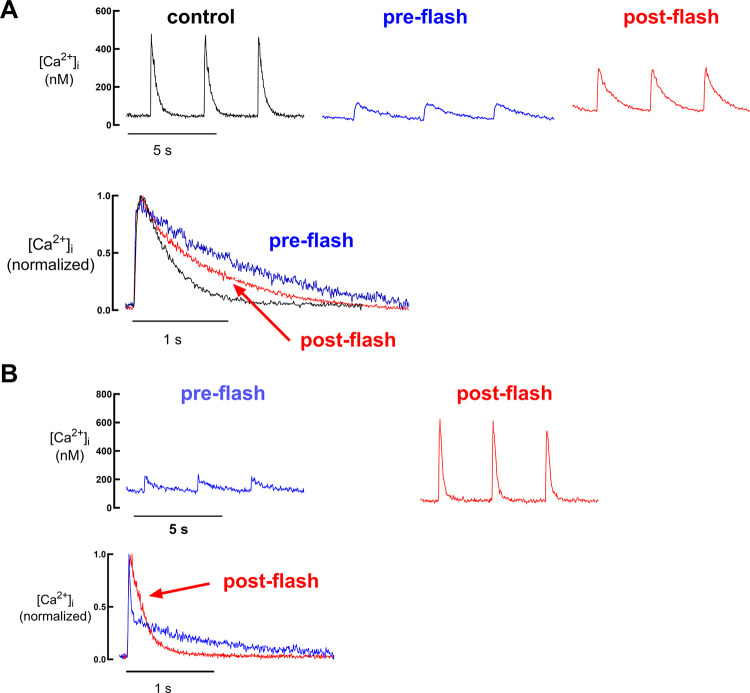
Effects of exogenous buffers on cardiac myocytes. *A*: fast buffer. *Top*: measurements of intracellular calcium concentration [Ca^2+^]_i_; from *left* to *right*: control cell; after loading with the photolabile Ca^2+^ chelator nitr-5; after flash photolysis of nitr-5. *Bottom*: averaged, normalized traces. *B*: slow buffer. *Top*: recordings from a cell loaded with NP-EGTA before (*left*) and after (*right*) flash photolysis. *Bottom*: normalized traces. Figures redrawn from Ref. 199, with permission from *Biophysical Journal*.

The effects of experimentally altering Ca^2+^ buffering in cardiac muscle are shown in FIGURE 12 (199). Here, buffering was first increased by adding a photolabile Ca^2+^ chelator. Buffering was then decreased by photolysis. As expected from FIGURE 7, the fast buffer nitr-5 decreases the amplitude and slows the kinetics of the Ca transient (FIGURE 12*A*). In contrast (FIGURE 12*B*), the slow buffer NP-EGTA results in a biphasic decay (cf. FIGURE 8 and FIGURE 9).

#### 6.1.1. Contribution of TnC and SERCA.

As shown in TABLE 5, TnC is expected to be the major buffer at diastolic levels of [Ca^2+^]_i_. As discussed in sect. 2.5.1.1, TnC has two classes of Ca^2+^ binding sites: *1*) the regulatory Ca^2+^ site, which has a *K*_d_ of ∼0.6 µM, and *2*) a pair of nonspecific “Ca^2+^/Mg^2+^” sites with a much lower *K*_d_ for both Ca^2+^ (0.003 µM) and Mg^2+^ (3.3 µM) (discussed in sect. 6.1.3). At diastolic Ca^2+^ levels, these sites bind significant amounts of Ca^2+^ and Mg^2+^. Experimental studies have investigated the effects of increasing the binding affinity of the regulatory sites of TnC with a single amino acid substitution (L48Q). With an adenoviral technique, ∼20% of the TnC in a rat heart was replaced. As expected, this increased myocyte contraction but did not alter the rate of decay of the [Ca^2+^]_i_ transient (295), suggesting little effect on buffering. There are two possible explanations. *1*) If TnC accounts for slightly less than 50% of total buffering (TABLE 5), then 20% replacement will decrease total buffering by only 10%, resulting in only a small effect on the decay of the [Ca^2+^]_i_ transient. *2*) A decrease of *K*_d_ will increase buffering at low and decrease it at high [Ca^2+^]_i_ (*Eq. 14*), leading to a mixed effect on relaxation. Although both factors will presumably contribute, the importance of the former is supported by a subsequent study expressing a higher concentration of the same mutation in mice myocytes (50% replacement) resulting in slowing of the decay of the [Ca^2+^]_i_ transient (296). A recent study has emphasized the importance of TnC in buffering by comparing buffering and Ca^2+^ handling in myocytes from the right and left ventricle of rat hearts. This showed a lower buffer power in the right ventricle that was associated with, and suggested to result from, lower expression of TnC (297). The other major contribution to buffering is expected to come from binding to SERCA (94). Direct evidence for a role of SERCA in buffering comes from the observation that inhibiting it with thapsigargin decreases buffer power by 16% in mouse ventricular myocytes (298).

##### 6.1.1.1. cooperative buffering.

The cooperative nature of Ca^2+^ binding to some buffers has not generally been considered in cardiac Ca^2+^ buffering. SERCA binds Ca^2+^ cooperatively, and this would be expected to produce a bimodal dependence on [Ca^2+^]_i_ of the buffer power contributed by fast buffers, with buffer power being low at both low and high [Ca^2+^]_i_ (TABLE 5). This contrasts with the simple hyperbolic buffer curves shown in the literature, where buffer power decreases with increasing [Ca^2+^]_i_ (180, 254, 287, 297, 299, 300). It is therefore possible that TABLE 5 overestimates the contribution of cooperative buffers. Equally, the experimental studies may have insufficient accuracy to resolve sigmoidal binding curves. A limitation of these studies is the lack of data below the normal resting [Ca^2+^]_i_ of ∼100 nM, and it will be important to obtain this. As pointed out in sect. 3.3, despite having only one regulatory site TnC may also show cooperativity due to interactions along the thin filament. Such effects would be expected to be different between isotonic and isometric contractions (sect. 6.2.3) and, given the lack of detailed information, are ignored in TABLE 5.

#### 6.1.2. The role of other fast buffers.

TABLE 5 shows that the buffer power provided by both SERCA and TnC falls as [Ca^2+^]_i_ increases to micromolar levels. During Ca^2+^ release, the Ca^2+^ concentration in the dyads may reach levels of up to 100 µM (55). Even at 10 µM [Ca^2+^]_i_, the combined contribution of TnC and SERCA is predicted to produce a buffer power of only 0.5. This raises the question as to what, if anything, provides buffering at this high [Ca^2+^]_i_. TABLE 5 suggests that total fast buffer power is ∼10, contributed largely by CaM, ATP, and carnosine. In other words, these buffers, which make only a modest fractional contribution to buffering at diastolic and systolic levels, may be important during Ca^2+^ release. Not only will they buffer Ca^2+^, but ATP and carnosine will accelerate its diffusion away from the release sites (TABLE 3). However, as mentioned in sect. 2.4.1, there is controversy as to whether carnosine buffers Ca^2+^ appreciably under physiological conditions. More work is required to investigate carnosine’s buffering role and the extent to which this may be altered, for example, in heart failure and other disease.

#### 6.1.3. The role of “slow” buffers.

**FIGURE 13.**
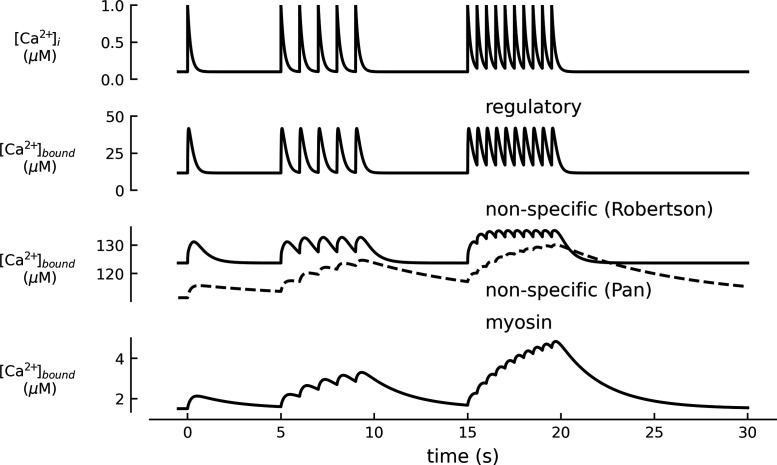
Simulation of Ca^2+^ binding to slow sites in cardiac muscle. The model simulates the effects of cytoplasmic Ca^2+^ transients. Traces show (from *top* to *bottom*) intracellular calcium concentration ([Ca^2+^]_i_); Ca^2+^ bound to the regulatory site of Troponin-C (TnC); Ca^2+^ bound to the nonspecific sites on TnC, comparing rate constants taken from Refs. 78, 301 (solid) and Ref. 79 (dashed); Ca^2+^ binding to myosin. Note the different vertical scales for TnC and myosin binding. The model shows the effect of (from *left* to *right*) a single stimulus; 1-Hz stimulation; and 2-Hz stimulation. The free Ca^2+^ transient was modeled with an instantaneous increase from 0.1 to 0.8 µM followed by an exponential decay (time constant 0.2 s).

Above, we have ignored the slower buffers, including the nonspecific or Ca^2+^/Mg^2+^ sites on TnC as well as Ca^2+^ binding to myosin. The nonspecific sites on TnC are present at twice the concentration of the regulatory sites and have a high affinity for Ca^2+^ with an apparent *K*_d_ for Ca^2+^ (in the presence of 1 mM Mg^2+^) of ∼100 nM (78, 79, 301). This means that they are ∼50% occupied by Ca^2+^ and contribute a buffer power of ∼350 at a diastolic level of Ca^2+^ of 100 nM. However, the relevance of this buffer power will depend on the kinetics and, specifically, on whether appreciable binding occurs during a [Ca^2+^]_i_ transient. This is illustrated by the difference between the instantaneous and steady-state values of buffer power in TABLE 5. Two experimental studies have provided very different measurements as exemplified by values for k_off_ of 0.33 s^−1^ (78) and 0.032 s^−1^ (79). This 10-fold difference in Ca^2+^ unbinding kinetics results in different predictions for the time course of Ca^2+^ occupancy of the sites as shown in FIGURE 13 (78, 79, 301). The faster kinetics predict some beat-to-beat change of bound Ca^2+^ and therefore buffering during the Ca^2+^ transient. In contrast, the slower kinetics result in essentially no beat-to-beat changes but a gradual increase of bound Ca^2+^ at higher frequencies. Either kinetic may lead to significant problems in the interpretation of experimental studies. For example, maneuvers that increase the time-averaged level of [Ca^2+^]_i_ will result in a slow loading of these sites (82). This will occur when stimulation is resumed after a quiescent period (FIGURE 13) or during the application of many agents that increase systolic [Ca^2+^]_i_. On cessation of stimulation, the Ca^2+^ gradually dissociating from these slow buffers is expected to keep both cytoplasmic and SR Ca^2+^ concentrations elevated for a period. The subsequent gradual decrease of [Ca^2+^]_i_ might erroneously be ascribed to movements of Ca^2+^ across SR or surface membranes. We are unaware of work investigating this possibility. The other major Ca^2+^ binding site on the myofilaments, which can also bind Mg^2+^, is on myosin, with a high apparent *K*_d_ for Ca^2+^ of ∼9 µM. As shown in FIGURE 13, Ca^2+^ binding to myosin is also slow, and the high *K*_d_ predicts that the amount of Ca^2+^ bound, even during repetitive stimulation, is modest and is unlikely to make a major contribution to total buffer power.

#### 6.1.4. SR buffers.

The above section has concentrated on the cytoplasmic buffers. The major Ca^2+^ buffer in the SR is calsequestrin, CSQ2. As well as acting as a buffer, it also interacts with triadin and junctin in regulating the RyR (302). The Ca^2+^ buffering properties of the SR have been measured in rabbit ventricular myocytes by measuring free SR [Ca^2+^] with Fluo-5N and estimating changes of total SR [Ca^2+^] from the corresponding changes of total cytoplasmic Ca^2+^ (as assessed from changes of [Ca^2+^]_i_ and cytoplasmic buffering). The buffering could be represented by a single buffer present at a concentration of 2.5 mM with a *K*_d_ of ∼0.6 mM (Ref. 303; see also Ref. 293). Even at very low [Ca^2+^], this would give a buffer power of only ∼4. With a loaded SR (typical free [Ca^2+^] of ∼1 mM) there will be 1.6 mM bound Ca^2+^. Two points are worth emphasizing: *1*) The buffer power of the SR is much less than that of the cytoplasm. *2*) The low buffer power means that complete removal of the major buffer (CSQ2) only decreases total Ca^2+^ by ∼60% and significant Ca^2+^ release from the SR can occur in the absence of CSQ2. Indeed, the CSQ2 knockout (KO) mouse shows normal cardiac contractility, and the loss of CSQ2 is compensated for by an increase of SR volume (304). As discussed in sects. 3.3.1 and 7.5, in skeletal muscle the binding of Ca^2+^ is cooperative and promoted by polymerization of CSQ2. In contrast, there is no evidence for cooperativity in the cardiac measurements (303). Although this may simply reflect a lack of sufficiently precise data, it is also possible that the low concentration of CSQ2 decreases the probability of polymerization. Further work is required to look for such cooperativity. Increasing the amount of CSQ2 prolongs Ca^2+^ release from the SR, an effect attributed to maintaining free SR content (305). Some mutations have been shown to decrease Ca^2+^ binding capacity (306). A decrease in CSQ2 concentration or mutations results in abnormal Ca^2+^ release from the SR, and this is one of the causes of the inherited arrhythmia syndrome CPVT (catecholaminergic polymorphic ventricular tachycardia) (307). At least in transgenic mouse models, removal of CSQ2 leads to a compensatory increase of another ER/SR Ca^2+^-binding protein, calreticulin (308). Calsequestrin is discussed more extensively in the context of skeletal muscle (sect. 7.5).

### 6.2. Factors Affecting Cardiac Calcium Buffering

Cardiac contraction is controlled by the amplitude of the systolic Ca^2+^ transient, which is generally assumed to be regulated by the size of the Ca^2+^ fluxes into and out of the cytoplasm. An important, and unresolved, issue is the extent to which changes of the amplitude of the [Ca^2+^]_i_ transient seen in both physiology and disease may also result from changes of Ca^2+^ buffering as opposed to those of Ca^2+^ fluxes. In other words, is Ca^2+^ buffering constant over time? We now consider some of the factors that may alter buffering. These include changes of [Ca^2+^]_i_ as well as of the properties of the buffers.

#### 6.2.1. Calcium concentration.

As discussed above (sect. 3.1) and also previously (82, 309, 310), for noncooperative buffers the available buffer power is affected by the level of [Ca^2+^]_i_, tending to decrease as [Ca^2+^]_i_ increases (see TABLE 5). This will have two consequences: *1*) An increase of diastolic [Ca^2+^]_i_ will decrease buffer power. This raises the possibility that the positive inotropic effects of maneuvers that increase diastolic [Ca^2+^]_i_ such as increased stimulation rate (311–313) and the application of cardiac glycosides (314) include a contribution from decreased Ca^2+^ buffering. Direct experimental work is required to assess this. The decreased buffer power at elevated [Ca^2+^]_i_ also complicates comparing buffer power between different animals, for example in the context of heart failure versus control. It is important to exclude the possibility that changes of buffer power are simply a consequence of differences of diastolic [Ca^2+^]_i_ as opposed to alterations of the buffer properties. This is not helped by the relative paucity of absolute measurements of diastolic [Ca^2+^]_i_ in different species (for review see Ref. 315). *2*) More generally, Ca^2+^ concentration-dependent changes in Ca^2+^ buffer power are predicted to contribute to shaping the time course of [Ca^2+^]_i_ transients as suggested in sect. 4.3 and FIGURE 10 and FIGURE 11. For example, the fact that the rate constant of decay of the Ca^2+^ transient increases with the amplitude of the transient is at least in part due to decreased buffering at increased [Ca^2+^]_i_ (316). Another example is the initial rapid phase of the decay of the [Ca^2+^]_i_ transient, which can be explained by the decreased buffer power at elevated [Ca^2+^]_i_ since a given rate of total Ca^2+^ removal by SERCA produces a larger fall of [Ca^2+^]_i_ (197).

#### 6.2.2. Phosphorylation of buffers.

A further question is whether the properties of cardiac cytoplasmic Ca^2+^ buffers can vary at fixed [Ca^2+^]_i_. The apparent *K*_d_s for Ca^2+^ of the two major buffers (SERCA and TnC) are regulated by phosphorylation [of phospholamban and troponin I (TnI), respectively], raising the possibility that this will also affect buffering. An experimental study, however, found no effect of beta adrenergic stimulation on Ca^2+^ buffering (298), and it was suggested that this resulted from the fact that, while phosphorylation of phospholamban increases the Ca^2+^ affinity of SERCA phosphorylation of troponin decreases its affinity, with the net result being no change. It should, however, be noted that previous work has not explicitly considered that the effects of changes of buffer *K*_d_ are expected to depend on the level of [Ca^2+^]_i_ (sect. 3.2). It is possible that the lack of change of buffer power could reflect a smearing of opposite effects above and below the value of [Ca^2+^]_i_ where changing *K*_d_ has no effect. Precise measurements of buffer power over a broader range of [Ca^2+^]_i_ are required.

#### 6.2.3. Effects of muscle length.

One special feature of TnC is that its location on the myofilaments makes it potentially sensitive to force development, with an increase of force increasing the affinity of Ca^2+^ binding. Direct measurements of [Ca^2+^]_i_ in cat papillary muscles showed that rapid stretches and releases resulted in transient decreases and increases of [Ca^2+^]_i_ consistent with Ca^2+^ being taken up by or released from TnC, respectively (317, 318). A maintained increase of muscle length was found to accelerate the initial rate of decay of the [Ca^2+^]_i_ transient and slow down the final phase, with both effects attributed to increased affinity of Ca^2+^ binding to troponin (319, 320). This is consistent with the analysis in sect. 3.2 and the simulations of FIGURE 10 illustrating that increased buffer affinity decreases buffering at higher [Ca^2+^]_i_ but increases it at lower [Ca^2+^]_i_. The former effect will accelerate the initial decay and the latter prolong the final phase (see also Ref. 321). Stretch has previously been reported to increase Ca^2+^ release from the SR (322). A recent study showed that the rise of [Ca^2+^]_i_ was increased by the actomyosin inhibitor blebbistatin (sect. 6.2.5), suggesting that, in the absence of blebbistatin, stretch increases the binding affinity of TnC, thereby attenuating the rise of [Ca^2+^]_i_ (323).

#### 6.2.4. Effects of pH on cardiac Ca^2+^ buffering.

As mentioned in sect. 2.5.1.2, changes of pH may significantly affect Ca^2+^ binding to buffers, either because of direct competition between protons and Ca^2+^ for binding sites or via effects on the tertiary structure of the buffers. An example is the decrease of Ca^2+^ binding to troponin produced by acidification (324). This interaction provides a potential mechanism linking changes of intracellular pH to those of [Ca^2+^]_i_. It is important to consider the relative magnitude of the effects on intracellular pH and [Ca^2+^]_i_. For example, a displacement of 20 µM total Ca^2+^ from TnC and its one-to-one replacement by protons will elevate [Ca^2+^]_i_ by 200 nM (given a Ca^2+^ buffer power of 100). In contrast, the typical intracellular pH buffer power is 30 mM per pH unit (325), so the absorption of 20 µM protons would be expected to change intracellular pH by 0.02/30 ≈ 0.0007 pH units. Although the change of [Ca^2+^]_i_ would be easily measurable, that of pH would not. An acid-induced increase of [Ca^2+^]_i_ has been measured in cardiac muscle (93, 194). However, as pointed out previously (82, 326) and in sect. 4.1, buffers cannot change the steady-state level of [Ca^2+^]_i_ and one would expect that the Ca^2+^ ions released would be pumped out of the cell, thereby restoring [Ca^2+^]_i_. In this context, it is worth noting that effects of protons on various membrane channels including acid-sensing ion channels (ASICs) and TRPV channels have been shown to contribute to changes of [Ca^2+^]_i_ in rat ventricular myocytes (327) and that protons also affect NCX activity (328). A further complication arises from the finding that changes of intracellular pH of the order of 0.4 pH units do not produce measurable changes of buffer power (329). Again, this may be a consequence of the fact that the direction of the effect of a given change of *K*_d_ on buffer power is opposite at high and low [Ca^2+^]_i_ (see sect. 3.2). A resolution of this issue will require further studies measuring buffer power as a function of both [Ca^2+^]_i_ and pH.

#### 6.2.5. Effects of modulators of actin-myosin interactions.

Given the importance of TnC to buffering, it would not be surprising if modulating Ca^2+^ binding directly or via interaction with other sarcomeric proteins affected buffering. Compounds such as EMD57033 are contractile sensitizers that increase TnC’s apparent affinity for Ca^2+^. In mouse ventricular myocytes, EMD57033 had no effect on B_max_ but decreased the buffer *K*_d_ (180). A recent study investigated its effects on Ca^2+^ buffering in cardiac myocytes derived from human induced pluripotent stem cells (iPSCs). Again, there was no effect on B_max_, and the *K*_d_ decreased from ∼0.47 to 0.25 µM (300). This was accompanied by a slowing of the decay of the [Ca^2+^]_i_ transient. The authors also calculated the rate of decrease of total Ca^2+^ from the measured buffering, which was unaffected by the drug, consistent with the conclusion that the slowing of decay of free Ca^2+^ is due to buffering. Strictly speaking (see sect. 3.2), one would expect the decrease of *K*_d_ to only increase buffer power at [Ca^2+^]_i_ below the geometric mean of the *K*_d_s (here 0.34 µM), with decreased buffering and therefore a faster decay of [Ca^2+^]_i_ above this level. It would therefore be interesting to study the effect of EMD57033 over a wider range of [Ca^2+^]_i_. The effects of such contractile sensitizers are of more than academic interest, as members of this group such as levosimendan (330) and omecamtiv mecarbil (331) have been developed for use in heart failure.

In contrast, other drugs decrease actin-myosin interactions, and one example is the myosin ATPase activity inhibitor blebbistatin (332). This can decrease the apparent affinity for Ca^2+^ to activate force (333, 334). One might therefore expect that it would lower the affinity for Ca^2+^ buffering by TnC, but we are unaware of any direct measurements. Whereas blebbistatin is simply an experimental tool, another inhibitor, mavacamten (see Ref. 335 for review), has been developed as a treatment for hypertrophic cardiomyopathy (336). As mentioned in sect. 6.4, mavacamtem decreases the affinity for Ca^2+^ activating contraction (337), and it is therefore also important to characterize its effects on Ca^2+^ buffering.

### 6.3. Ca^2+^ Buffers and Ca^2+^ Diffusion in Cardiac Muscle

As discussed in sect. 4.5, mobile Ca^2+^ buffers accelerate the diffusion of Ca^2+^. The major cardiac Ca^2+^ buffers, SERCA and TnC, are immobile, and the best-characterized mobile buffers are CaM and ATP. As we have seen above, the contribution of CaM to diffusion of Ca^2+^ is negligible, particularly since the majority of CaM is bound. With the values of TABLE 3, the flux of Ca^2+^ carried by Ca-ATP will be ∼1.4 times that of free Ca^2+^ over a wide range of [Ca^2+^]_i_, thereby making a significant contribution. It is also worth noting that there is up to 20 mM carnosine and other histidyl dipeptides in cardiac muscle (211). As mentioned in sect. 2.4.1, there is controversy as to their degree of Ca^2+^ binding, but, if significant, these will also contribute greatly to Ca^2+^ diffusion.

What effect will such Ca^2+^ diffusion mediated by mobile buffers have? As discussed below for skeletal muscle (sect. 7.6), diffusion is required for Ca^2+^ ions to move from the sarcoplasmic reticulum release sites to the TnC on the myofilaments (34, 338), and therefore the mobile buffers, in particular ATP, will accelerate activation of the myofilaments. We are unaware of any experimental evidence in cardiac muscle, and it will be important to obtain this. This effect of mobile buffers will promote cardiac contractility. Set against this, however, is the fact that excitation-contraction coupling depends on local Ca^2+^ release, observed as Ca^2+^ sparks. Larger [Ca^2+^]_i_ transients result from the summation of closely spaced individual Ca^2+^ sparks (339). These sparks are spatially independent, and this is important for controlling cardiac contractility in a stable way (340). Increasing Ca^2+^ diffusion will potentially increase the spatial extent of Ca^2+^ sparks and remove this independence. One consequence of loss of spatial independence of sparks is the generation of propagating Ca^2+^ waves. These are not seen normally but occur under conditions where Ca^2+^ spark frequency (341) and leak from the SR are increased, because of either an increase of SR Ca^2+^ content or alterations in the properties of the RyR (for review see Ref. 342). Modeling has suggested that such waves can propagate by a “fire-diffuse-fire” mechanism. Importantly, the speed of propagation of the waves is proportional to the apparent diffusion constant for Ca^2+^ (343) and would therefore be expected to be increased by mobile buffers. In particular, at least for IP_3_-induced Ca^2+^ waves, for a given buffer concentration calculations suggest that the more mobile the buffer, the faster the wave propagation velocity (344). Another modeling study predicted that Ca^2+^ waves are more likely to appear at low cytoplasmic buffer concentrations and that, at a given buffer concentration, the higher the *K*_d_ for the buffer the more likely are waves (345). At least at low [Ca^2+^]_i_, high *K*_d_ and low buffer concentration will both decrease buffer power, suggesting that increased buffering decreases wave occurrence. It would be useful to extend this modeling to consider mobile buffers.

### 6.4. Ca^2+^ Buffering in Cardiac Disease

Several studies have examined whether Ca^2+^ buffering changes in cardiac disease. One example is atrial fibrillation, the most common cardiac arrhythmia (346). When this was simulated with rapid electrical pacing in rabbits, a large (2- to 3-fold) increase of buffer power was observed that was attributed to decreased phosphorylation of TnI decreasing the *K*_d_ for Ca^2+^ binding to TnC (299). This was accompanied by (and suggested to cause) a failure of the Ca^2+^ release to propagate from the periphery to the interior of the cell. A threefold decrease of *K*_d_ would increase buffer power by the same factor at low [Ca^2+^]_i_ (sect. 3.1). At higher [Ca^2+^]_i_, however, buffer power would increase by less and would be expected to decrease above a [Ca^2+^]_i_ given by the geometric mean of the *K*_d_s in control and rapid pacing. This effect on propagation could be mimicked by the addition of BAPTA to increase buffering. Addition of EGTA similarly stops propagation in normal feline atrial myocytes (347). It should be noted that the waves occur because atrial myocytes from small animal species contain relatively few transverse tubules and therefore Ca^2+^ release initially occurs at the periphery where the SR and surface membrane are in contact (348). However, atrial myocytes from larger species, including human, have a more complete network of t tubules, and Ca^2+^ release also occurs in the center of the cell, making excitation-contraction coupling less dependent on Ca^2+^ waves (349–351). This may impact on the broader relevance of this decreased wave propagation mechanism in atrial fibrillation. A decrease of buffer power was seen in a sheep model of atrial fibrillation, where it was suggested to facilitate the spread of Ca^2+^ release and thereby maintain fibrillation (352). Also in sheep atrium, rapid pacing to induce heart failure decreased buffer power (353). In contrast, a recent study found no difference in Ca^2+^ buffering between myocytes taken from human atrium with or without postoperative atrial fibrillation (354). The origin of these disparate findings is unclear and may be in part due to the different species and models studied. Further work is required to resolve this and also the possibility that changes of diastolic [Ca^2+^]_i_ may have contributed.

Many studies have shown that heart failure is associated with increased Ca^2+^ sensitivity of the contractile machinery, possibly as a result of decreased phosphorylation of troponin I (334, 355–357) or myosin binding protein C (358, 359). It is therefore surprising that direct measurements have found that ventricular Ca^2+^ buffering is unaffected by heart failure (360, 361). Two factors need to be considered: *1*) Increased Ca^2+^ sensitivity does not necessarily imply increased affinity for Ca^2+^, as it is possible that events downstream from changes in Ca^2+^ binding are augmented, giving an apparent increase of Ca^2+^ affinity. *2*) An increase of Ca^2+^ affinity of buffers will increase buffering at low [Ca^2+^]_i_ but decrease it at higher (sect. 3.2). Measuring buffer power over the whole range of [Ca^2+^]_i_ will average out these changes and result in no apparent change of buffer power. More detailed measurements are therefore required.

In humans, hypertrophic cardiomyopathy (HCM) frequently results from mutations in thin filament proteins including troponin and tropomyosin (362). Mutations in these and other proteins are also associated with dilated cardiomyopathy (DCM) (363). Many of the HCM mutations have been shown to increase the affinity of Ca^2+^ binding, whereas those producing DCM decrease it (364). Troponin mutations associated with HCM slow the decay and decrease the amplitude of the [Ca^2+^]_i_ transient (180, 365). These effects could result from an increase of buffer power due to the increased affinity, although, as pointed out previously (180), buffer power would only be expected to increase at low [Ca^2+^]_i_ (in the range of [Ca^2+^]_i_ below the geometric mean of the *K*_d_s). The myosin inhibitor mavacamtem decreased the apparent Ca^2+^ affinity of such HCM mutations and reversed the slowing of the decay of the [Ca^2+^]_i_ transient (337), and it is therefore possible that these effects also result from normalization of buffering. However, buffering was not measured. The increase of buffering and therefore of total Ca^2+^ in HCM has been suggested to increase Ca^2+^ loading of the SR and thence increased arrhythmogenic Ca^2+^ release during diastole, partly accounting for the increased arrhythmia burden (180). The link between increased Ca^2+^ binding and arrhythmias is supported by the fact that those mutations that have the greatest effect on Ca^2+^ binding are also the most arrhythmogenic (333). A modeling study has, however, questioned whether increasing Ca^2+^ sensitivity does promote arrhythmias in human HCM (366). Increased buffering also leads to shortening and triangulation of the AP (333), findings that were reproduced in both work on cardiac myocytes derived from human iPSCs (367) and computer simulations (368). It was suggested that the AP shortening results from the increased Ca^2+^ buffering decreasing the amplitude of the Ca^2+^ transient and thence the inward NCX current, which contributes to maintaining the normal AP plateau (367). Such electrophysiological alterations can also be arrhythmogenic. A significant factor that predisposes to arrhythmias is that of electrical alternans (a condition in which the AP duration alternates on a beat-to-beat basis). This is often accompanied by alternation in the amplitude of contraction and the underlying Ca^2+^ transient (for recent review see Ref. 369) and is a particular issue when the alternation is inhomogeneous throughout the ventricle (370). It is therefore interesting to note that HCM mutants can increase the probability of such electrical alternans (367). Modeling has also indicated that changes of Ca^2+^ affinity would be expected to affect the likelihood of alternans (371), with an increase of Ca^2+^ sensitivity increasing alternans in the atrium (368).

The study referred to in sect. 6.2.5 on iPSC-derived cardiomyocytes compared control cells with those derived from patients with a troponin T mutation leading to DCM. The mutation was associated with increased affinity of Ca^2+^ buffering (*K*_d_ = 0.32 µM in mutant vs. 0.40 µM in control), with no effect on maximum buffer power (300). It is worth noting (see sect. 3.2) that with these values of *K*_d_ buffer power will only be increased in a range of [Ca^2+^]_i_ below ∼0.35 µM. At higher [Ca^2+^]_i_, the mutant troponin will have a lower buffer power. The decreased buffer *K*_d_ was accompanied by increased occurrence of alternans, and this link was reinforced by showing that the myofilament Ca^2+^ sensitizer EMD57033 increased both buffering and alternans. This contrasts with the suggestion (above) that DCM mutations have lower affinity. It may be accounted for by the decreased phosphorylation of troponin I leading to increased affinity of Ca binding found in iPSC-derived cardiomyocytes (372). Nevertheless, this change of buffering was associated with increased occurrence of alternans, suggesting a link between altered Ca^2+^ handling and the arrhythmias seen in DCM.

## 7. SKELETAL MUSCLE

**FIGURE 14.**
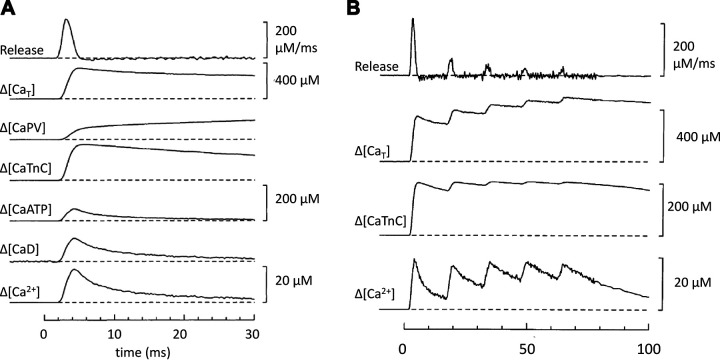
The role of buffers in skeletal muscle. *A*: changes of free and bound Ca^2+^ during a twitch in a mouse fast-twitch fiber. Records show (from *top* to *bottom*) rate of release of Ca^2+^ from sarcoplasmic reticulum (SR); changes of total calcium concentration (Δ[Ca_T_]); [Ca-parvalbumin (PV)]; [Ca-Troponin-C (TnC)]; [Ca-ATP]; Ca^2+^ bound to the indicator (furaptra) (CaD); and intracellular [Ca^2+^] ([Ca^2+^]_i_). Note that [Ca^2+^]_i_ decays more quickly than total; [Ca-TnC] falls while [Ca-PV] is still increasing. *B*: changes during tetanic stimulation. Traces show (from *top* to *bottom*) rate of release of Ca^2+^; total [Ca^2+^]; [Ca-TnC]; and [Ca^2+^]_i_. Note that the release flux and increase of total [Ca^2+^] on the second stimulus is much less than on the first, but the change of [Ca^2+^]_i_ is almost comparable. Figure taken from Ref. 377, with permission from the *Journal of Physiology*.

The intracellular Ca^2+^ signals in skeletal muscle control body movements lasting from <1 s to many minutes. Excitation-contraction (EC) coupling is the process whereby an action potential (AP) lasting only a few milliseconds generates a twitch with a duration of hundreds of milliseconds. Most movements are initiated by a short volley of APs (20–50 Hz) in a motor neuron innervating a group of skeletal muscle fibers, resulting in a train of skeletal muscle APs and tetanic contraction (373). There are two main subtypes of skeletal muscle fibers, fast and slow twitch. This distinction is based on the kinetics of contraction, and different muscles have predominantly one or the other fiber type. Muscles involved with posture are generally slow twitch and are more energetically efficient than the predominantly fast twitch involved in rapid limb movements (374, 375). A skeletal muscle AP triggers a pulse of Ca^2+^ release from the SR of ∼1-ms duration at rates of 200 µM/ms (34, 376) (for review see Ref. 84). SERCA-mediated Ca^2+^ uptake then returns the released Ca^2+^ back to the SR over ∼100 ms. The timescale of the release event is comparable to the half-time of Ca^2+^ binding to TnC, ∼1.5 ms (84), and therefore the Ca^2+^-TnC interaction does not reach equilibrium during a twitch (see also FIGURE 14 and Ref. 377). As demonstrated by [Ca^2+^]_i_ measurements and computational models, the kinetics and relative affinities of the cytoplasmic buffers determine the rapid and sustained contractile responses to the [Ca^2+^]_i_ signal (83, 84). It is important to note that the levels of [Ca^2+^]_i_ reached in skeletal muscle [up to ∼20 µM (84)] are considerably greater than those in cardiac muscle.

### 7.1. Skeletal Muscle Ca^2+^ Buffers

Several methods have been used to obtain direct measurements of Ca^2+^ buffering in skeletal muscle. An early approach used frog skeletal muscle fibers with the ends cut to allow control of the intracellular environment. Buffer power was increased by raising the concentration of the Ca^2+^ indicator Antipyrylazo III (APIII) (378). As expected, the higher the concentration of indicator, the slower the decay of [Ca^2+^]_i_. Extrapolating this relationship to zero indicator suggested that ∼25% of the cytoplasmic Ca^2+^ was free, with the remainder bound to endogenous buffers, giving a rather low value of ∼4 for buffer power. The authors pointed out that the accuracy of this method was limited by the accuracy of the estimate of the Ca^2+^ affinity of the indicator, although it is not clear that this could reconcile this low buffer power with the higher ones discussed below. In later work using the same technique, a higher buffer power of 16 was obtained (379), and this was suggested to have a significant contribution from TnC. Another study (on intact fibers) used the Ca^2+^ indicator Arsenazo III at high concentrations such that the bulk of the Ca^2+^ release from the SR would be bound to the indicator (225). This allowed a minimum estimate of the Ca^2+^ release from the SR of ∼100 µM. With a lower indicator concentration, the rise of free [Ca^2+^]_i_ was found to be ∼4 µM. This would suggest a buffer power of ∼25, but, as mentioned, the estimate of SR content is a minimum estimate so that the actual buffer power could have been up to ∼37.

Most research into the effects of Ca^2+^ buffers on Ca^2+^ signaling and contraction in skeletal muscle has used concentrations and affinities of the buffers found in biochemical studies (e.g., Ref. 78). The main intracellular Ca^2+^ buffers for fast- and slow-twitch fibers are listed in TABLE 6. Such lists have been used in studies designed to investigate the interrelationship between Ca^2+^ fluxes from the sarcoplasmic reticulum and the resulting contraction (83, 84, 225, 376). As well as giving information about the steady-state contributions to buffer power of the various buffers, TABLE 6 also gives values for the average buffer power on increasing [Ca^2+^]_i_ from a resting value of 50 nM to either 2.0 or 20 µM. Two values are given, “instantaneous” and “steady state.” The former assumes that the fast buffers such as TnC and SERCA will have reached equilibrium with [Ca^2+^]_I_, whereas the latter (higher) value assumes that all buffers are equilibrated. Note that the contribution of the slower buffers will be much greater during a tetanus compared to a single twitch.

**Table 6.** Contributions of various compounds to calcium buffering in skeletal muscle, fast-twitch fibers, and slow-twitch fibers in the mouse

Buffer	Conc, µM	*K*_app_, µM	Hill Slope	Binding Sites	β*_i_*(Ca) 50 nM (ss)	β*_i_*(Ca) 2.0 μM (ss)	β*_i_*(Ca) 20 μM (ss)	β*_i_*(ΔCa) 50 nM-2.0 μM (in)	β*_i_*(ΔCa) 50 nM-2.0 μM (ss)	β*_i_*(ΔCa) 50 nM-20 μM (in)	β*_i_*(ΔCa) 50 nM-20 μM (ss)	Ref.
*Fast-twitch fibers*												
SERCA1a	120	0.30	1.9	2	283.8	5.9	0.0	115.9	115.9	11.6	11.6	98
TnC (reg)	120	1.30	1	2	171	28.7	0.7	70.0	70.0	10.8	10.9	84
Carnosine	10,000	1,350	1	1	7.4	7.4	7.2	7.4	7.4	7.2	7.3	49
Parvalbumin	75	0.14	1	2	574	4.7	0.1	4.6	51.9	0.5	5.5	83
ATP	8,000	2,496	1	1	3.2	3.2	3.2	3.2	3.2	2.9	3.2	33
Myosin	120	9.10	1	2	26.1	17.7	2.6	0.4	21.5	0.0	8.2	78
TnC (nonspec)	120	0.10	1	2	1,060	5.5	0.1	1.6	76.6	0.2	8.0	78
Total					2,125	73	14	203	347	33	55	
*Slow-twitch fibers*												
SERCA2a	48	0.60	1.9	2	31.9	7.6	0.0	44.3	44.3	4.8	4.8	98
TnC (reg)	120	1.30	1	1	86	14.3	0.3	35.0	35.0	5.4	5.4	84
Carnosine	10,000	1,350	1	1	7.4	7.4	7.2	7.4	7.4	7.2	7.3	49
Parvalbumin	7.5	0.14	1	2	57	0.5	0.0	0.5	5.2	0.0	0.6	83
ATP	5,000	2,496	1	1	2.0	2.0	2.0	2.0	2.0	1.8	2.0	33
Myosin	120	9.10	1	2	26.1	17.7	2.6	0.4	21.5	0.0	8.2	78
TnC (nonspec)	120	0.10	1	2	1,060	5.5	0.1	1.6	76.6	0.2	8.0	78
Total					1,270	55	12	91	192	19	36	

From *left* to *right*: cytoplasmic concentration; *K*_app_; Hill slope (*n*); no. of binding sites; steady-state (ss) buffer power [β*_i_*(Ca)] at 0.05, 2, and 20 µM Ca^2+^ (calculated with *Eq. 8* or *16*); and the buffer power for a change in [Ca^2+^] [β*_i_*(ΔCa)] for 0.05–2 µM and 0.05–20 μM Ca^2+^, calculated from the change in Ca^2+^ binding (*Eq. 5* or *15*). steady-state binding (ss) was calculated based on equilibrium at both Ca^2+^ and Mg^2+^ sites. Instantaneous buffer power (in) was calculated assuming equilibration of Ca^2+^ binding but no change in the Mg^2+^ binding state in order to indicate the relative extent of the fast and slow buffers. SERCA, sarco(endo)plasmic reticulum Ca^2+^-ATPase; TnC, Troponin-C.

The concentrations of the Ca^2+^ binding sites on the major fast buffers (TnC and SERCA) are greater than in cardiac muscle (TABLE 5). In contrast, the buffer powers reviewed above in skeletal muscle are lower than in cardiac muscle. This is partly a consequence of the much higher levels of [Ca^2+^]_i_ in skeletal muscle and the consequent buffer saturation. For example, TABLE 6 shows that, in fast-twitch fibers, the buffer contribution produced by the sum of SERCA and the regulatory sites on TnC is 455 at 50 nM [Ca^2+^]_i_ but only 0.7 at 20 µM [Ca^2+^]_i_. Another study, on frog skeletal muscle, used depolarizing pulses to activate the L-type Ca^2+^ current. Ca^2+^ release from the SR was inhibited with EGTA so that the observed increase of [Ca^2+^]_i_ could be assumed to originate from the Ca^2+^ current. The change of total Ca^2+^ (calculated from the integral of the current) was then compared with the measured free [Ca^2+^]_i_. With this approach, a high buffer power of ∼150–190 was calculated (380). This study was performed over a lower range of [Ca^2+^]_i_ (<1 µM) than occurs during twitches and tetani, and this (TABLE 6) would contribute to a larger buffer power. Two complications should be noted. *1*) There will be a contribution of ∼14 from the Ca^2+^ indicator (again APIII), and *2*) in these experiments the muscle also contained 1 mM EGTA, which will make a major contribution to buffering. As mentioned above, there is still controversy about the buffer role of carnosine and other HDPs under physiological conditions. As previously reviewed (381), carnosine, the major member of the HDP family, has many biological roles including acting as an antioxidant and intracellular pH buffer. In skeletal muscle, it sensitizes contraction to activation by Ca^2+^ (382) but may also have a role as a mobile calcium buffer analogous to that of ATP (338).

### 7.2. The Role of Parvalbumin

The cytoplasmic CBP parvalbumin is an example of a pure buffer: a protein with apparently no role in skeletal muscle other than to shape the intracellular Ca^2+^ signal and thereby influence function (61). It also plays an important role as a buffer in many neurons (see sect. 9.2). The PVs are a group of small acidic proteins (MW 11,000–12,000) with three EF-hand motifs, two of which can bind Ca^2+^ at cytoplasmic Ca^2+^ concentrations There are two major forms, α-PV and β-PV, arising from separate genes; the PVβ group includes the protein oncomodulin (see Ref. 383 for review). In the α form of PV (present in mammalian skeletal muscle), the two EF-hand motifs have similar binding affinities, are Ca^2+^/Mg^2+^ sites, and do not show significant cooperativity (29, 83, 384). PV is present at higher concentrations in smaller compared with larger mammals and, in a given species, in fast- compared with slow-twitch fibers (385). The conversion by high-intensity exercise of fast- to slow-twitch fibers is accompanied by a decrease of PV concentration (386). In humans, only very low levels of PV are expressed in both fiber types, and it is only found in the intrafusal fibers (muscle spindles); the reason for this difference is not known (385). Increase of PV concentration by direct gene transfer accelerates mechanical relaxation (387).

The EF hands of PV bind Mg^2+^ ions in competition with Ca^2+^. The dissociation constant of Mg^2+^ is ∼10^4^ times greater than that for Ca^2+^, but since cytoplasmic free Mg^2+^ levels are ∼10^4^ times higher than Ca^2+^, PV binds comparable amounts of the two ions, leaving only a small amount of PV unbound even in a relaxed muscle. Using the values of TABLE 6 for fast-twitch fibers, at rest ([Ca^2+^]_i_ = 0.05 µM) 68% of PV will have Mg^2+^ bound, 26% is Ca^2+^ bound, and 6% remains unbound. This unbound fraction will contribute to rapid buffering; the time course of the larger subsequent buffering by Ca^2+^ binding to the Mg^2+^ sites will depend on the rate constant of dissociation of Mg^2+^ [k_off,Mg_ = 3 s^−1^ (34)] as the subsequent Ca^2+^ association rate constant is rapid [k_on,Ca_ = 42 µM^−1^·s^−1^, equivalent to a pseudo-first-order rate constant of 42 s^−1^ at 1 µM Ca^2+^ (83)]. Despite being a slow buffer, the buffer capacity of PV is significant, particularly in the case of some fast-twitch muscles. This is illustrated in TABLE 6, in the form of the calculation of an “instantaneous” (in) versus “steady-state” (ss) buffer power for mouse skeletal muscle fibers.

The role of PV in mechanical relaxation depends on the fact that it binds Ca^2+^ slowly via the Mg^2+^ exchange mechanism such that binding continues when [Ca^2+^]_i_ decreases and Ca^2+^ dissociates from TnC (87). The relaxation of [Ca^2+^]_i_ and contraction therefore reflects a sequential mechanism: Ca^2+^ is initially bound to TnC and is then taken up by PV before being removed by SERCA into the SR (see Ref. 388 for review). Binding of Ca^2+^ to PV allows [Ca^2+^]_i_ to decrease quickly at the end of the SR release phase as the slow displacement of Mg^2+^ from PV provides a buffer in addition to SR Ca^2+^ uptake and therefore accelerates the decrease in cytoplasmic Ca^2+^ (83, 376). As shown in FIGURE 14*A*, as Ca^2+^ dissociates from TnC, it binds to PV (377). Under experimental conditions, PV can promote full mechanical relaxation, albeit at a slow rate, even when SERCA is virtually completely inhibited (389). This can also be seen in the simulation of FIGURE 9*D*. This additional boost to Ca^2+^ removal will only occur at the end of a twitch or brief tetanus; longer tetani will cause increased Ca^2+^ binding to PV as it displaces bound Mg^2+^, leading to saturation of PV (83, 390, 391, 564). This explains why PV is expressed at higher concentrations in fast-twitch muscle in smaller mammals (see sect. 7.3). For example, in rat skeletal muscle the faster relaxation of a twitch compared to that after a long tetanus is more prominent in fast- compared with slow-twitch fibers (390), consistent with the presence of PV in the former but not the latter (385).

Adding EGTA, a slow buffer, can also accelerate the rate of [Ca^2+^]_i_ decay (392). A subsequent study characterized [Ca^2+^]_i_ transients accompanying tetani in mouse fast- and slow-twitch muscle fibers. Slow-twitch muscle fibers show a stimulus-to-stimulus increase of [Ca^2+^]_i_ during the initial phase of the tetanus. Such a staircase is absent in fast-twitch fibers. Addition of the exogenous buffer EGTA to a slow-twitch fiber virtually abolished this staircase and made the [Ca^2+^]_i_ time course resemble that of a fast-twitch fiber (393). Although alternative mechanisms exist to explain this data, these findings are consistent with the role of PV in shaping the fast-twitch Ca^2+^ transient, and measurements and simulations confirm this mechanism (377).

The high concentration of PV in fast-twitch fibers of small mammals enhances relaxation in an energetically efficient manner. A decrease of [Ca^2+^]_i_ produced by binding to PV does not consume ATP (see sect. 4.2). ATP is required subsequently as SERCA eventually lowers [Ca^2+^]_i_, leading to Ca^2+^ dissociation from PV. However, the peak SERCA rate is much lower than would be the case in the absence of PV (FIGURE 9 and Ref. 394). This role of PV comes at the price of a higher buffer power at resting [Ca^2+^]_i_ (0.05 µM; TABLE 6), which would in part explain the higher Ca^2+^-storage capacity of the SR in fast-twitch fibers as larger Ca^2+^ release is required to achieve comparable loading of the contractile proteins (225). This distinction between rapid and slow effects of PV is also relevant to neurons (see sects. 9.4–9.6). Related to this, the lower temperature dependence of PV kinetics compared to those of SERCA increases the relative contribution of PV to relaxation at lower temperatures (388, 395). As pointed out in sect. 7.7, modification of PV to increase the affinity for Mg^2+^ would decrease the concentration of free PV and therefore the buffering during Ca^2+^ release. It would, however, also decrease the ability to buffer Ca^2+^ during relaxation, and it is possible that the existing values represent an optimum balance between effects during Ca^2+^ release and relaxation. This is supported by recent data from an inducible mouse PV knockout that showed increased peak twitch and tetanic Ca^2+^ acutely in fast-twitch fibers (396) such that short-term fatigue was reduced, despite the slower rate of the early phase of decay of the intracellular Ca^2+^ signal at the end of a tetanus while longer-term fatigue was unaffected. This work contrasts with earlier work that showed greater long-term fatigue in a constitutive PV knockout model and parallel increases in mitochondrial volume and elevated resting [Ca^2+^]_i_ (397).

There are several significant cytoplasmic Ca^2+^/Mg^2+^ buffers in skeletal muscle, but PV shows the greatest difference between the instantaneous and steady-state buffer power at Ca^2+^ concentrations normally achieved during tetani. As shown in TABLE 6, in fast-twitch fibers the steady-state contributions of the TnC nonspecific (Ca^2+^/Mg^2+^) sites and of myosin are much less than provided by PV. In slow-twitch fibers (TABLE 6) these sites may play a larger role, but we can find no experimental data testing this.

Finally, an interesting example of the importance of PV is provided by the woodpecker, which drums its beak against a tree at a rate of almost 20 s^−1^. The neck muscles responsible for this drumming have much higher PV concentrations than both other skeletal muscle in the woodpecker and in the neck muscles of nondrumming species (398).

### 7.3. Differences in Buffering between Fast-Twitch and Slow-Twitch Fibers

**FIGURE 15.**
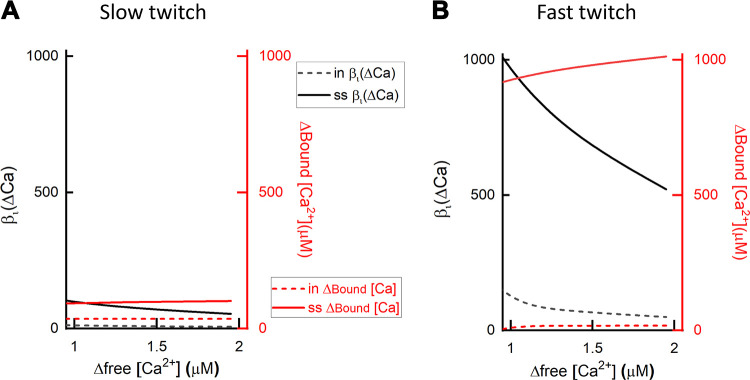
Theoretical parvalbumin (PV) buffer and Ca^2+^ binding curves in mouse skeletal muscle. *A* and *B*: buffer power [β(ΔCa)] and the change in total Ca^2+^ binding to PV assuming a resting (initial) Ca^2+^ of 50 nM. Black lines (dashed and solid) show the buffer power for a range of changes in free Ca^2+^ (Δfree[Ca^2+^]) that is normally experienced during a tetanus. The dashed black lines represent the initial buffer power (before dissociation of Mg^2+^ from PV), corresponding to the initial 20–30 ms [“instantaneous” values, in β*_i_*(ΔCa)]. The solid black lines show steady-state values calculated assuming the equilibrium of Ca^2+^ and Mg^2+^ with PV that would be approached during tetani lasting >200 ms [“steady-state” values, ss β_i_(ΔCa)]. Red lines and *right* axis represent the concentration of Ca^2+^ bound to PV at the instantaneous time point (in ΔBound [Ca^2+^]) and that during the steady state (ss ΔBound [Ca^2+^]).

The calculated buffer power differs considerably between fiber types because of differences in expression of proteins associated with Ca^2+^ signaling and contraction. (399). TABLE 6 lists the levels of the major cellular buffers found in small mammals such as mice (83, 84) and shows a much higher buffer power in fast- compared to slow-twitch fibers at both 0.05 and 2.0 µM [Ca^2+^]_i_. The greater concentration of SERCA in fast-twitch fibers corresponds to a larger SR Ca^2+^ store in this fiber type and the higher number of SR Ca^2+^ release sites compared with slow-twitch fibers (84). This arrangement generates a larger Ca^2+^ release during E-C coupling and along with the cooperative binding of Ca^2+^ at two sites on TnC (compared to only a single site in slow twitch), generating a faster rate of contraction in fast fibers. As discussed in sect. 7.2, the major contributor to intracellular buffering at [Ca^2+^]_i_ values normally achieved during tetani (up to 20 µM) is PV, which is expressed at ∼10-fold higher levels in fast-twitch compared with slow-twitch fibers (385). The Ca^2+^ binding and buffer powers due to PV for the two fiber types are shown in FIGURE 15 and illustrate the minimal additional Ca^2+^ binding and Ca^2+^ buffering attributable to PV both immediately after SR Ca^2+^ release (instantaneous) and during sustained (>200 ms) tetani (steady state) in slow-twitch muscle fibers. This contrasts with the very large additional binding and the associated buffer power that operates in mouse fast-twitch muscle with 10-fold higher concentrations of PV (see TABLE 6).

### 7.4. The Role of Buffer Saturation in Tetanic Contraction

Another example of the physiological consequences of buffer kinetics for muscle function involves the Ca^2+^ binding events at the start of a tetanic contraction. As shown in FIGURE 14*B*, the initial SR Ca^2+^ release from the first in a train of action potentials (APs) raises total cytoplasmic Ca^2+^ by ∼350 µM, resulting in an increase of free [Ca^2+^]_i_ of ∼20 µM. Free and total [Ca^2+^] as well as Ca^2+^ bound to TnC then begin to decay as a result of SR reaccumulation by SERCA; however, [Ca^2+^]_i_ is still elevated at the time of the next stimulus (∼20 ms later). Since the SR Ca^2+^ content is decreased (not shown), this AP results in a smaller increase of total cytoplasmic Ca^2+^. However, this smaller SR Ca^2+^ release will be added on top of an already elevated [Ca^2+^]_i_ with the majority of high-affinity Ca^2+^ binding sites being occupied. This occupancy reduces the cytoplasmic Ca^2+^ buffer power considerably, and although the second SR Ca^2+^ release is much smaller than the first, it is still sufficient to raise [Ca^2+^]_i_ to a peak value almost as high as that produced by the first AP, maintaining an almost maximal Ca^2+^ binding to TnC (84, 179, 400). It should be noted that the slow kinetics of PV means that it does not substantially influence the time course of [Ca^2+^]_i_ during such high-frequency stimulation. The interval between consecutive APs is critical. Too short intervals will attenuate APs and SR Ca^2+^ release. Too long intervals will allow the free Ca^2+^ concentration to decay sufficiently to decrease Ca^2+^ binding to TnC, and therefore contraction will not be maintained (179). The net effect is that a train of APs in skeletal muscle generates an immediate (<10 ms) increase in contractile activity that is sustained at close to maximal levels for the duration of the tetanic train. This effect of buffer saturation is analogous to that seen for buffer saturation-induced synaptic facilitation (sect. 9.5).

This principle also explains the benefit of doublet or triplet firing patterns recorded commonly in motor neurons of fast mammalian skeletal muscle fibers (373, 401). The first two or three APs are separated by shorter intervals (e.g., 5 ms) compared with the intervals in the rest of the train (e.g., 30 ms) (402). The doublet/triplet pattern of motor neuron firing ensures a rapid step change in contractile activity and sustained force response during a brief tetanic response (179) by sustaining [Ca^2+^]_i_ for the initial 10–15 ms.

It has been suggested that cytoplasmic Ca^2+^ buffering may have significant effects on the level of [Ca^2+^]_i_ reached during a tetanus (see Ref. 400 for review). For example, acidosis decreases force while increasing [Ca^2+^]_i_, and both effects can be attributed, at least in part, to decreased binding of Ca^2+^ to troponin (403). In this context, it is worth emphasizing an important difference between these effects and what would be expected in cardiac muscle. Ca^2+^ fluxes across the sarcolemma are small in skeletal muscle, so a decrease of Ca^2+^ binding to buffers would be expected to produce an increase of [Ca^2+^]_i_ that will be maintained for longer than would be the case in the heart. Another mechanism by which altered buffering may be involved in fatigue has been proposed. The increase of cytoplasmic phosphate concentration, as a result of breakdown of phosphocreatine, has been suggested to increase the SR phosphate concentration, leading to precipitation of Ca phosphate in the SR and a reduction of Ca^2+^ release (404, 405). It should, however, be noted that changes of metabolites have a variety of other effects on Ca^2+^ handling (see Ref. 406 for review).

### 7.5. The Role of Calsequestrin

The concentration of free Ca^2+^ in the SR ([Ca^2+^]_SR_) of skeletal muscle is ∼300–600 µM (112, 407, with considerable Ca^2+^ also bound to CSQ (for review see Ref. 213). The major isoform in skeletal muscle is CSQ1 (408). The properties of SR Ca^2+^ buffering have been studied in intact frog skeletal muscle fibers in which the ends were cut to allow incorporation of indicators into the cytoplasm and SR. A high concentration of EGTA was added for two reasons: *1*) to suppress changes of cytoplasmic [Ca^2+^]_i_, allowing the added Ca^2+^ indicator (tetramethylmurexide) to measure changes of [Ca^2+^]_SR_ uncontaminated by those of cytoplasmic [Ca^2+^]_i_ (409), and *2*) Ca^2+^ released from the SR will bind to cytoplasmic EGTA releasing protons. The resulting change of intracellular pH gives a measure of the change of total SR Ca^2+^ concentration (256). This method gave a value (in frog skeletal muscle) of 670 µM for free and 17.1 mM for total SR Ca^2+^ with ∼30 mM CSQ. It was concluded that ∼95% of the Ca^2+^ released from the SR came from that bound to CSQ as opposed to free Ca^2+^. The SR buffering could be fit with a Hill coefficient of 3.0, indicating pronounced cooperativity of Ca^2+^ binding (cf. sect. 3.3). A subsequent study measured SR Ca^2+^ buffering in mouse skeletal muscle by comparing changes of free SR Ca^2+^ with those of calculated total Ca^2+^ assessed from changes of [Ca^2+^]_i_ measured with a cytoplasmic indicator (410). This provided an average SR buffer power of 157. Measurements in myocytes of CSQ knockout mice showed that buffer power was reduced to 25%, indicating that CSQ is the major SR Ca^2+^ buffer. The SR Ca^2+^ buffer curve could be fit with a Hill coefficient of 3.52, again indicating cooperative binding. As discussed in sect. 3.3, the cooperativity of Ca^2+^ binding has been associated with polymerization of CSQ. This was investigated in intact skeletal muscle by using fluorescence recovery after bleaching (FRAP) to measure the diffusion of CSQ in the SR. Depleting the SR of Ca^2+^ increased the diffusion coefficient, indicating depolymerization (411). The cooperative nature of Ca^2+^ binding to CSQ has been suggested to have important physiological consequences (411, 412).

It is not simple to relate the biophysical properties of SR Ca^2+^ buffering to the known biochemical properties of CSQ (410). During Ca^2+^ release from the SR, the decrease of free [Ca^2+^] from ∼1 mM to 50 µM will cause depolymerization of the CSQ filament and a reduction in the available binding sites and therefore potentially buffer power (20, 132). Whereas the on and off rate constants of Ca^2+^ binding to the individual chelation sites are relatively rapid, the kinetics of the polymerization-depolymerization processes, which would determine the overall buffer kinetics, are unknown. Estimates of dimerization kinetics have been derived from the analysis of records of [Ca^2+^]_SR_ signals from smooth muscle (189). More direct measurements are required, particularly since it is uncertain what degree of CSQ polymerization occurs within the SR lumen of different muscle types. EM images of the terminal cisternae of skeletal muscle show extensive branching networks of CSQ suggesting a high degree of polymerization (213, 413). However, the estimate of the maximum [Ca^2+^]_SR_ in skeletal and cardiac muscle is ∼1 mM. Based on biochemical measurements, this concentration would only result in CSQ dimers and not higher-order polymers (132, 133). It is, of course, possible that the Ca^2+^ sensitivity of polymerization is affected by cellular constituents absent in biochemical studies. Furthermore, the law of mass action would suggest that the higher CSQ concentration found in fast-twitch SR compared to that used in the biochemical studies would favor polymer formation. The degree of polymerization may also be expected to be affected by the CSQ concentration, something that has yet to be studied in detail.

If CSQ did not buffer Ca^2+^ cooperatively, it would have its lowest buffer power at high [Ca^2+^]_SR_ (FIGURE 3). Release of a given amount of Ca^2+^ would result in a large decrease of [Ca^2+^]_SR_, thereby increasing buffer power and decreasing the release flux. In contrast, the high buffer power of the cooperative CSQ buffer maintains [Ca^2+^]_SR_ and thence Ca^2+^ release (213). Direct measurements of [Ca^2+^]_SR_ have shown the occurrence of abrupt rises of [Ca^2+^]_SR_ attributed to depolymerization of CSQ and therefore release of bound Ca^2+^ (411, 414). These papers also advanced an explanation for muscle fatigue with the idea that a decrease of [Ca^2+^]_SR_ would (because of the cooperative nature of Ca^2+^ buffering) decrease the buffer power and thereby the Ca^2+^ release. In this context it is noteworthy that mutations in CSQ that alter polymerization and reduce Ca^2+^ binding have been associated with disease, including malignant hyperthermia and vacuole aggregate myopathy (415–417).

### 7.6. Diffusion of Ca^2+^ in Skeletal Muscle

The release of Ca^2+^ from the terminal cisternae of the SR results in a high local, cytoplasmic Ca^2+^ signal (376). To enable contraction, this Ca^2+^ must diffuse from the terminal cisternae of the SR, located near the Z lines (418), to bind to TnC molecules, located along most of the sarcomere, at a distance of up to 1 µm away. Modeling studies suggested that Ca^2+^ diffusion is accelerated by binding to ATP (34). An experimental study using frog skeletal muscle fibers compared the rise time of [Ca^2+^]_i_ at the release sites (Z line) with that in the middle of the sarcomere (M line). The latter lagged by 2–3 ms, and these relative kinetics could be explained by a model including diffusion of CaATP (338). The situation may be different in mammalian skeletal muscle, where the Ca^2+^ release triads reside at the A-I border as opposed to the Z line (419), placing them closer to the TnC and thereby decreasing the diffusion distance and the need for acceleration of Ca^2+^ diffusion by mobile buffers. Note that skeletal muscle contains significant amounts of the mobile buffer carnosine (212) (cf. sect. 2.4.1). Subject to current uncertainties about its Ca^2+^ binding, this may also aid Ca^2+^ diffusion.

### 7.7. The Role of Buffers in Skeletal vs. Cardiac Muscle

The above sections emphasize an important difference between the role of buffers in these two types of striated muscle. There are four important roles of Ca^2+^ buffers in skeletal muscle: *1*) SR Ca^2+^ buffering by CSQ limits the fall of Ca^2+^ that occurs during Ca^2+^ release from the SR. *2*) Mobile buffers help transfer Ca^2+^ ions from the SR release sites to the myofibrils. *3*) Buffering by PV accelerates relaxation. *4*) Buffer saturation increases the rise of [Ca^2+^]_i_ during a tetanus. In contrast, in cardiac muscle only the first two of these occur. Furthermore, the degree of SR Ca^2+^ buffering by CSQ is considerably greater in skeletal compared with cardiac muscle, perhaps reflecting a higher concentration of CSQ (409). Functionally, the difference may reflect the fact that depletion of the cardiac SR on a single beat does not matter as the SR is refilled before the next beat. Indeed, the decrease of free Ca^2+^ may contribute to closing of RyRs and thence termination of Ca^2+^ release (420). In contrast, the SR will gradually deplete during a tetanus in skeletal muscle, and the higher CSQ content will minimize this.

Other differences between buffering in cardiac versus skeletal muscle do not result from the differences in the buffers expressed but rather from the time course and concentration range of [Ca^2+^]_i_. As discussed above, the higher peak [Ca^2+^]_i_ in skeletal muscle will result in decreased buffer power due to saturation, and the prolonged rise of [Ca^2+^]_i_ during a tetanus will increase contributions from slow buffers.

The role of PV to accelerate relaxation in skeletal muscle contrasts with the fact that Ca^2+^ buffers slow relaxation of cardiac muscle. The explanation of this paradox is the slow kinetics of PV, which works in parallel with SERCA to reduce [Ca^2+^]_i_ in fast-twitch skeletal fibers. In particular, the kinetics of PV are sufficiently slow that it continues to take up Ca^2+^ even when [Ca^2+^]_i_ is declining. There is no equivalently slow buffer in cardiac muscle or slow-twitch skeletal fibers. In this context, it has been suggested that expression of a PV-like buffer in cardiomyocytes might be a useful therapeutic approach in heart failure as it could accelerate relaxation without decreasing contraction. PV binds Ca^2+^ too slowly to interfere appreciably with peak [Ca^2+^]_i_ in systole but would bind significant amounts of Ca^2+^ during diastolic relaxation (421, 422). It has further been shown that β-PV performs better in this respect because it binds Mg^2+^ better than α-PV (423). This will increase the concentration of the Mg^2+^-bound and decrease that of the free form of the buffer, thereby decreasing the rapid component of buffering and allowing it to accelerate relaxation with less effect on the amplitude of the systolic [Ca^2+^]_i_ transient and thence contractility. A similar improvement has been reported for single amino acid mutations in the α-PV EF-hand domain (424, 425).

## 8. SMOOTH MUSCLE

Several studies have measured the Ca^2+^ buffer power in smooth muscle by using the method described in sect. 6.1 in which cells are depolarized to activate L-type Ca^2+^ currents. The measured Ca^2+^ current and inferred total Ca^2+^ can then be compared with the rise of [Ca^2+^]_i_ measured with an indicator dye. The resulting value of buffer power includes contributions from both the endogenous buffers and the indicator used to measure [Ca^2+^]_i_. A value of 82 was reported for toad stomach (426). A similar approach in rat portal vein myocytes gave a buffering power of 170, which, corrected for the contribution of the Ca^2+^ indicator, provided an endogenous buffering power of 114 (427). A smaller buffer power of 30–40 was reported for guinea pig urinary bladder smooth muscle cells (428). In guinea pig colonic smooth muscle cells, a much higher buffer power (∼400) was measured. This was reduced to 250 by the addition of ryanodine, suggesting that some of this apparent buffer power represented Ca^2+^ uptake into the SR (429), suggesting the lower value as the better estimate. In guinea pig coronary vascular smooth muscle cells, a buffer power of 300 was obtained, which corresponded to ∼150 when corrected for the contribution by the fluorescent indicator (430). A different study using the added buffer method (173) (sect. 9.1) provided a buffer power of 46 (431). Further research is required to clarify whether the wide range of reported buffer power values reflects real differences between the various types of smooth muscle as opposed to being a consequence of methodological differences. It is, however, worth noting that the lowest buffer power mentioned above was obtained with changes of [Ca^2+^]_i_ of ∼4 µM (428) whereas the other studies typically involved maximum [Ca^2+^]_i_ of 250–800 nM. It is therefore possible (cf. sect. 3.1) that the lowest buffer power values might result from buffer saturation. Given this, it is challenging to model buffering. A computer model of smooth muscle Ca^2+^ movements used a lumped buffer with a total concentration of 230 µM and *K*_d_ of 1 µM, equivalent to a rather high buffer power at low [Ca^2+^]_i_ of 230 µM/1 µM = 230 (432).

What is the origin of the measured Ca^2+^ buffering? One potential contributor is CaM, which is present at concentrations of 34 and 40 µM in guinea pig taenia coli (433) and cultured bovine tracheal cells (434), respectively. Taking 40 µM and the *K*_app_s listed in TABLE 5 (187), one calculates a buffer power of 4 at 100 nM [Ca^2+^]_i_. This value is lower than most of the buffer powers reported above. The affinity for Ca^2+^ binding increases when CaM is bound to proteins (435), and this would be expected to increase buffer power. A study using electron probe microanalysis found that the increase of total cytoplasmic Ca^2+^ accompanying a contracture produced by the combination of KCl-induced depolarization and norepinephrine was ∼235 µM (436). The authors pointed out that this was considerably greater than what might be expected to bind to CaM. They therefore suggested that Ca^2+^ must also bind to myosin and possibly other proteins. It has, however, been questioned whether myosin binds significant amounts of Ca^2+^ in the presence of cytoplasmic Mg^2+^ concentrations (see Ref. 437 for review). The reader will note that the studies referred to in this paragraph are mainly >30 years old. In their review published in 1986 (437), Sommerville and Hartshorne note, “A contemporary problem is to identify the various components of the Ca^2+^ buffer system.” It is our opinion that this problem is still “contemporary.”

A recent paper reported expression of calreticulin in endothelial cells forming myoendothelial junctions, which are important in coordinating calcium signaling between endothelium and smooth muscle. Much of the calreticulin expression was not associated with other ER markers, suggesting that it is located somewhere other than in the ER (438). Further work is required to establish whether this is present in the cytoplasm.

## 9. NEURONS AND NEUROENDOCRINE CELLS

As in muscle cells, Ca^2+^ ions function as the principal second messengers coupling action potential (AP) firing to diverse intracellular events, from activation of cytosolic signaling pathways to gene transcription. In addition, Ca^2+^ serves a dual neuron-specific function at chemical synapses: Ca^2+^ triggers synaptic vesicle fusion at presynaptic active zones, and Ca^2+^ is a pivotal signaling molecule controlling synaptic plasticity. The spatiotemporal characteristics of neuronal [Ca^2+^]_i_ transients are determined by Ca^2+^ influx, extrusion, and buffering. Ca^2+^ influx into somata, dendrites, and axon terminals occurs primarily in response to membrane depolarizations opening voltage-gated Ca^2+^ channels (VGCCs). In some neurons, Ca^2+^ influx through Ca^2+^-permeable ligand-gated channels and/or Ca^2+^ release from internal stores represent two additional routes to increase [Ca^2+^]_i_. AP-induced Ca^2+^ influx is generally brief, lasting at most a few milliseconds. At glutamatergic synapses equipped with postsynaptic NMDA channels, Ca^2+^ influx can last longer owing to the slow deactivation kinetics of these channels (227, 439). Nevertheless, global cytosolic [Ca^2+^]_i_ transients that remain after the collapse of local Ca^2+^ domains following channel closure can outlast the duration of Ca^2+^ influx by one or two orders of magnitude. These global cytosolic [Ca^2+^]_i_ transients are shaped by Ca^2+^ extrusion and buffering, which differ between different neuron types and different subcellular compartments and which temporally and spatially confine Ca^2+^ signals. The manner in which neuronal [Ca^2+^]_i_ transients are affected by Ca^2+^ buffers is determined by three parameters: *1*) their concentration in the cytosol, *2*) their affinity and binding kinetics for Ca^2+^ and Mg^2+^ ions, and *3*) their mobility in the cytosol.

### 9.1. Measuring Cytosolic Ca^2+^ Buffering in Neurons and Neuroendocrine Cells

**FIGURE 16.**
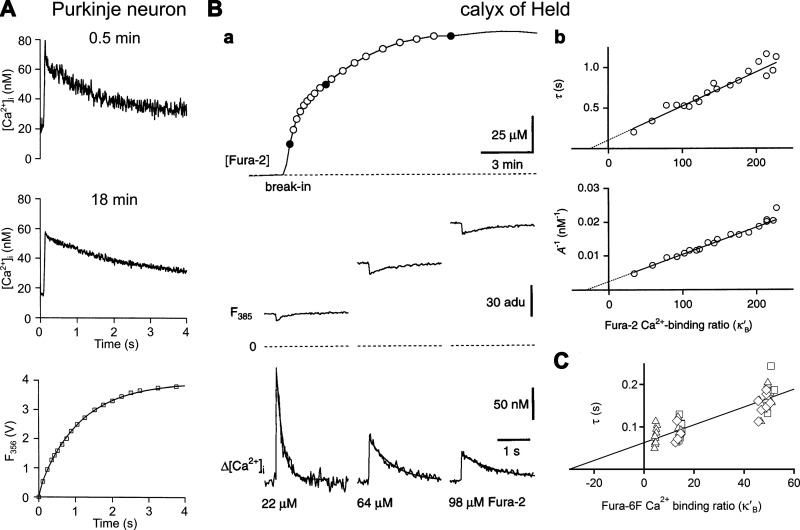
Estimating cytosolic Ca^2+^ buffering power by competition of endogenous with added exogenous buffers. *A*: somatic intracellular calcium concentration ([Ca^2+^]_i_) transients recorded in a cerebellar Purkinje neuron either shortly (*top*) or 18 min (*middle*) after establishing whole cell configuration with 500 µM fura-2 in the patch pipette. *Bottom*: the progressive loading of the cell with the dye. Amplitudes and decay kinetics of [Ca^2+^]_i_ transients are resistant to fura-2 loading via a patch pipette. Modified from Ref. 443, with permission from *Journal of Physiology*. *B*: similar experiment as in *A* performed in a calyx of Held nerve terminal. *a*: Loading of the terminal with 100 µM fura-2 monitored with the fluorescence at the isosbestic excitation wavelength (*top*). Fluorescence signals at 385-nm excitation (F_385_; *middle*) and corresponding AP-evoked [Ca^2+^]_i_ transients (*bottom*) are shown for 3 time points after establishing whole cell configuration (*top*; filled circles). With increasing dye concentration, [Ca^2+^]_i_ transients decrease in amplitude and decay more slowly (cf. FIGURE 7). *b*: Decay time constant τ (*top*) and inverse of the peak amplitude A^−1^ (*bottom*) plotted as a function of the fura-2 Ca^2+^-binding ratio $\kappa_{B}^{\prime }$ (exogenous buffer power) for various time points during dye loading. Regression lines through the τ and A^−1^ plots yield extrapolated *y*-axis intercepts for τ and A^−1^ at $\kappa_{B}^{\prime }$ = 0 (no added buffer). Negative *x*-axis intercepts represent estimates for 1 + $\kappa_{S}^{\prime }$ (endogenous buffer power). Modified from Ref. 440, with permission from *Biophysical Journal*. *C*: similar as in *B* except that Ca^2+^ influx was triggered by short trains of action potential (AP)-like depolarizations, a low-affinity dye was used, and plotted decay constants represent pooled data obtained from several terminals loaded with 3 different dye concentrations. Each data cluster corresponds to 1 of the 3 fura-6F concentrations. Modified from Ref. 248, with permission from *Journal of Physiology*.

Much work characterizing neuronal buffers has used the “added buffer approach” as originally applied to adrenal chromaffin cells (27, 174) and later extended to nerve terminals (248, 440) and dendrites of hippocampal neurons (59, 201, 441, 442). In these experiments, a whole cell recording is established on the compartment of interest, with a patch pipette containing a ratiometric Ca^2+^ indicator, usually fura-2 or one of its low-affinity analogs at a known concentration and therefore $\kappa_{B}^{\text{′}}$ value. Either single APs are elicited or short depolarizing pulses are applied under voltage clamp at various time points while the cell is loaded with the indicator dye (FIGURE 16) (248, 440, 443). Both the amplitudes (Δ[Ca^2+^]_i_) as well as the decay time constants τ_Ca,i_ of the resulting [Ca^2+^]_i_ transients are evaluated. According to the simple theory presented in Eqs. 31 and *32* (27, 174), plots of 1/A or τ against the amount of “added buffer power” allow the calculation of values for A and τ in the absence of exogenous buffers by extrapolation to zero added buffer (*y*-axis intercept, FIGURE 16*Bb*). The negative *x*-axis intercept of such plots is an estimate for the endogenous buffer power (1 + $\kappa_{S}^{\text{′}}$; FIGURE 16*Bb*). This method is therefore a refinement of the approach using different concentrations of Antipyrylazo III in skeletal muscle (sect. 7.1 and Ref. 378). Unlike the techniques presented above for cardiac and smooth muscle (sects. 6.1 and 8), where buffer power is estimated by comparing the movement of Ca^2+^ into the cytoplasm with the change of [Ca^2+^]_i_, the added buffer approach does not require knowledge of the cytoplasmic volume. However, it does not easily provide information about buffer type, its concentration, or affinity. In fact, several mobile and immobile buffer species may often contribute to the obtained buffer power estimates. The added buffer approach is typically used with small and compact cells such as adrenal chromaffin cells or with subcellular compartments of neurons such as somata or nerve terminals, for which it can be assumed that spatial [Ca^2+^]_i_ gradients collapse over a time span much shorter than typical values for the decay constants of [Ca^2+^]_i_ transients (>10 ms). The method is also suitable for cylindrical structures such as axons and dendrites under careful selection of uniform diameter, such that longitudinal gradients are minor. FIGURE 16 illustrates its use to estimate buffer power in neuronal cell bodies and nerve terminals. When it is applied to cerebellar Purkinje cells, their high endogenous buffer power results in dialysis with fura-2 having only small effects on measured [Ca^2+^]_i_ transients (FIGURE 16*A*). In contrast, much larger effects are observed (FIGURE 16, *B* AND *C*) in calyx of Held terminals because of their much lower buffer power.

**FIGURE 17.**
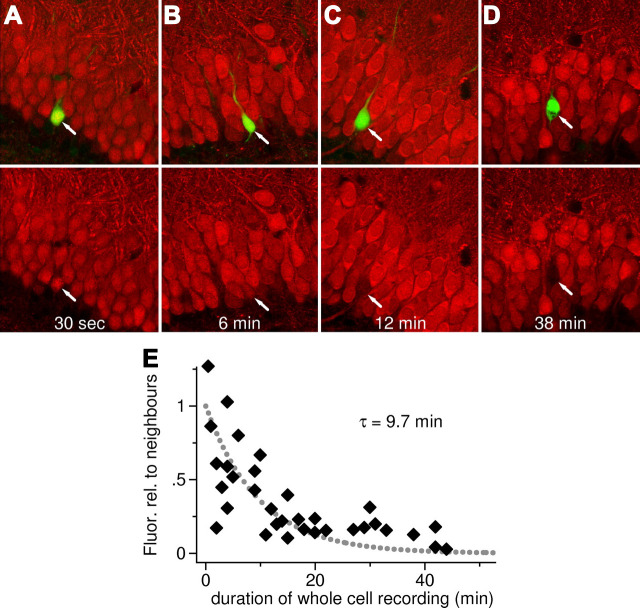
Loss of the endogenous mobile Ca^2+^ buffer CB-D28k during whole cell recordings in hippocampal dentate gyrus granule neurons (GCs). *A–D*: several GCs were recorded and filled with the fluorescent dye Lucifer yellow (LY) during whole cell recording episodes lasting for 30 s (*A*), 6 min (*B*), 12 min (*C*), or 38 min (*D*). Subsequently, hippocampal slices were fixed and stained for CB-D28k. Arrows indicate recorded neurons identified by their LY fluorescence. *E*: time course of CB-D28k loss during whole cell recordings. The CB-D28k immunofluorescence of each recorded GC was normalized to that of neighboring GCs. Modified from Ref. 444, with permission from *Journal of Neuroscience*.

As originally described (174), the results of the added buffer approach are somewhat ambiguous since it is not clear which types of endogenous buffer are being assayed. All fixed buffers are expected to be included, but mobile buffers may be washed out while the indicator dye is infused (FIGURE 17) (444). Typical buffers, with diffusion coefficients about four times smaller than that of fura-2 (TABLE 3), should be retained at the early time points of the whole cell recording and are therefore probably included. Small metabolites, however (sect. 2.4), may be washed out quickly. During extended recording periods, diffusional equilibration between pipette and cytoplasm is achieved also for larger buffer molecules such as calbindin-D28k, resulting in nearly complete washout of that mobile buffer with a time constant of ∼10 min (FIGURE 17*E*). At the same time gluconate^−^, often chosen as the main anion of the pipette solutions in these types of experiments, is infused together with the indicator. The gluconate anion binds Ca^2+^ weakly (60) and may well compensate for the loss of endogenous counterparts. Therefore, estimates of buffering power contributed by small low-affinity Ca^2+^ binders and determined by the original added buffer approach cannot be considered reliable. Although their contribution to the absolute value of buffering power is expected to be small, their influence on Ca^2+^ diffusion and on local domains may be substantial (see sects. 4.5. and 4.7.2). To remove this uncertainty and to focus on low-mobility and stationary buffers, Matthews, Schoch, and Dietrich (59) proposed a modification of the procedure: Instead of measuring changes in [Ca^2+^]_i_ during a single dye-loading experiment, several whole cell recordings are performed with different concentrations of indicator dyes in the pipette. [Ca^2+^]_i_ transients are elicited after dye loading is complete and mobile Ca^2+^ buffers are expected to have been washed out. Therefore, the plots of A^−1^ and τ plots versus $\kappa_{B}^{\text{′}}$ of the indicator, as described above, should report the buffering power of immobile and slowly mobile buffers, depending on the geometry of the cell under study and the waiting period before measurement. When applied to the calyx of Held nerve terminal, this method yielded an endogenous Ca^2+^ buffer power of 21 (248), whereas somewhat higher estimates (40–46) were obtained with the original procedure (440, 445).

The same problem was addressed in studies on bovine chromaffin cells (446) by “balanced dye loading.” In this approach, individual cells are preloaded with a certain concentration of fura-2, either by AM-ester loading or by dye preloading, during a brief whole cell recording episode. Subsequently, a perforated-patch recording is established, this time with a concentration of fura-2 in the pipette, which is expected to match the concentration inside the cell under study. Total Ca^2+^ buffer power, including mobile and immobile buffers, is estimated from total Ca^2+^ influx and changes in free [Ca^2+^]_i_ during short depolarizing voltage-clamp pulses. Once a stationary situation has been established, the perforated patch is ruptured, resulting in a whole cell configuration. Subsequent changes in buffering power are followed by repetitive application of depolarizing pulses. Experiments are considered successful if dye loading is “balanced,” meaning that total fura-2 fluorescence did not change rapidly after rupture. In that case, later changes in Ca^2+^ buffer power are interpreted as changes in endogenous buffers. No such changes were observed in some cells after “balanced loading,” indicative of little or no contribution to Ca^2+^ buffering from mobile buffers, which would have washed out. However, on a longer timescale about half of the cells displayed a partial drop in buffering power compatible with the loss of proteins of ∼7,000–20,000 molecular weight, which before their washout contributed ∼25% of the total buffering power of 40.

All versions of the added buffer approach discussed so far depend on the approximations discussed in sect. 4.7.1, employing *Eqs. 30–32* for the analysis of exponentially decaying [Ca^2+^]_i_ transients. When studying slow buffers, such as PV, which cause biphasic decays of [Ca^2+^]_i_ transients, more complex stimulus patterns and analysis procedures need to be invoked (27). Various other methods for measuring Ca^2+^ buffer power, and also for titrating endogenous buffers, have been described (251, 447, 448), similar to methods typically used when studying muscle cells (sects. 6.1 and 8). With photolysis of caged Ca^2+^ compounds, *K*_d_, Ca^2+^ binding rate constant, and Ca^2+^ binding ratio of the endogenous fixed buffer in adrenal chromaffin cells were estimated as ∼100 µM, 5.17 × 10^8^ M^−1^·s^−1^, and 40, respectively (449). Altogether, values of buffer power are typically in the range of 15–300; maximum values of ∼2,000 for Purkinje cell somata and dendrites were found. For a compilation of Ca^2+^ buffer powers and affinities of endogenous Ca^2+^ ligands see Refs. 173, 201. Thus, the fraction of free Ca^2+^ in the cytosol varies typically between ∼0.3% and ∼7% of total [Ca^2+^]_i_ at equilibrium but can be as low as 0.05%. The buffering power of fixed buffers is small but nevertheless the dominating one in many types of neurons. Higher buffering powers are primarily achieved by a higher abundance of endogenous mobile Ca^2+^ binding proteins and confer special characteristics of short-term synaptic plasticity and electrical excitability (see below).

### 9.2. Neuronal Expression of EF-Hand Domain Ca^2+^-Binding Proteins

In the mammalian brain, the predominant mobile Ca^2+^ buffers found in neurons are parvalbumin (PV/α-PV), calbindin-D_28K_ (CB-D28k), and calretinin (CR), which are members of the superfamily of EF-hand domain CBPs (sect. 2.5.1). Neurons typically express only one of these three proteins, but coexpression of combinations of two of the three EF-hand domain CBPs is also observed. Two other relevant molecules that bind Ca^2+^ rapidly and are present at high cytoplasmic concentrations in neurons, and therefore shape the spatiotemporal characteristics of local and/or global Ca^2+^ transients, are ATP (450) and CaM (451). CaM binds Ca^2+^ with faster kinetics than any of the three CBPs CR, CB-D28k, or PV (187).

The commonly applied criterion (sect. 2.5.1) that pure Ca^2+^ buffer proteins do not undergo marked conformational changes upon Ca^2+^ binding and do not interact with other proteins in a Ca^2+^-dependent way holds for PV among the family of EF-hand domain CBPs (565). In contrast, a substantial conformational change is observed upon Ca^2+^ binding to CB-D28k (31). The apparent diffusion coefficient for CB-D28k in the cytosol depends on the subcellular compartment, and an interaction of CB-D28k with *myo*-inositol monophosphatase has been identified in spines and dendrites of cerebellar Purkinje neurons, indicating that CB-D28k is both a Ca^2+^ buffer and a Ca^2+^-signaling molecule (452). A similar picture emerges with respect to CR: CR and P/Q-type (Ca_V_2.1) VGCCs coimmunoprecipitate from mouse cerebellum homogenate. In HEK293T cells, coexpression of CR attenuates Ca^2+^-dependent inactivation (CDI) and augments Ca^2+^-dependent facilitation (CDF) of heterologously expressed P/Q-type VGCCs via a direct interaction with the α_1_2.1 subunit (453). It remains to be established whether a modulation of VGCCs by CR as observed in HEK293T cells also occurs in neurons.

The expression of EF-hand domain CBPs in different brain regions has been studied extensively at the cellular and subcellular levels and in various species, once specific antibodies became available. It is the topic of several excellent reviews (454–460), and here we only summarize some key findings. PV is predominantly, but not exclusively, expressed in inhibitory interneurons of the brain. Many of these are fast-spiking GABAergic interneurons, including fast-spiking basket and chandelier cells of the neocortex and the hippocampus, fast-spiking striatal interneurons, two classes of GABAergic interneurons of the molecular layer of the cerebellum, stellate and basket cells, and many interneurons of the thalamus. PV is absent from pyramidal neurons of the neocortex and hippocampus, but it is expressed in large amounts in the output neurons of the cerebellum, the Purkinje cells, which additionally coexpress CB-D28k. PV is heavily present in somata and neuropil of the rodent auditory system, for example in auditory nerve fibers, and neurons of the spiral ganglia, the cochlear nucleus, and the inferior colliculus (461).

Prominent PV immunoreactivity is found in three types of large calyciform synaptic terminals: GABAergic terminals of interneurons in the thalamus (462) and glutamatergic endbulb of Held and calyx of Held terminals in the auditory brain stem (461). In subsets of the latter two types of auditory terminals coexpression of presynaptic CR but not CB-D28k was found (463, 464). Interestingly, the onset of PV expression in numerous classes of neurons of the auditory brain stem of mice and rats occurs relatively late and coincides approximately with the onset of hearing (461, 463).

CB-D28k is expressed in a variety of neurons, mostly interneurons, and the adult pattern of expression is in general established at birth. In the rat neocortex, CB-D28k-immunoreactive cells are predominantly found in the upper layers II and III (457). In the hippocampus, CB-D28k-immunoreactive interneurons are found in all subdivisions (465). CB-D28k is also a major Ca^2+^ buffer of hippocampal dentate gyrus granule cells and CA1 pyramidal neurons, whereas it is absent from CA3 pyramidal cells (444, 466). In the cerebellum, Purkinje cells are the only neurons expressing CB-D28k (455).

Similar to CB-D28k, CR is expressed in various interneurons of the neocortex, most abundantly in the upper layers II and III, and in specific interneurons of the hippocampus (467, 468). However, coexpression of CB-D28k and CR in individual neurons is rare (469). In the cerebellum, CR is expressed in the glutamatergic granule cells, which relay mossy fiber inputs to Purkinje cell dendrites via the parallel fiber pathway (455).

Expression of CBPs within the vertebrate retina varies among species. In the rodent, photoreceptors and bipolar cells lack expression of CBPs whereas some classes of horizontal, amacrine, and ganglion cells express one or more of CB-D28K, CR, and PV (470–472). In the rat cochlea, in situ hybridization shows exclusive expression of β-PV (oncomodulin) in outer hair cells (OHCs) whereas inner hair cells (IHCs) express both α-PV and β-PV (473). The developmental profile of PV isoform, CB-D28k, and CR expression revealed a transient β-PV expression during IHC development while the expression of CR and α-PV slightly increases. The sum of the CBP concentrations decreases in IHCs but increases in OHCs during cochlear maturation (474).

The selective expression of CBPs in distinct neuron populations of different brain areas suggests that specific functional properties of CBPs confer specific physiological properties to those neurons. This finding has been exploited experimentally to identify and/or selectively manipulate the respective neurons in situ. The availability of Cre-driver mouse lines for both PV and CR allows targeting PV- and CR-expressing neurons for genetic manipulations, including the expression of fluorescent markers, fluorophores for Ca^2+^-imaging, and channelrhodopsin and its derivatives enabling stimulation or silencing of specific neuron populations (475, 476).

### 9.3. Cytosolic Concentration of Ca^2+^-Binding Proteins

For some neurons and subcellular compartments, cytosolic concentrations of CBPs have been estimated either by using calibrated immunohistochemistry or via functional assays during which neurons lacking a certain CBP were loaded with recombinant protein to restore normal function.

Hippocampal dentate gyrus granule cells (hDG GCs) contain ∼40 µM CB-D28k, corresponding to ∼160 µM Ca^2+^ binding sites, as estimated by performing postrecording immunohistochemistry following whole cell dialysis with known concentrations of recombinant CB-D28k and comparing immunofluorescence intensities to that in neighboring, unperturbed neurons (444). Cytosolic CB-D28k concentrations for hippocampal CA3 stratum radiatum interneurons and CA1 pyramidal neurons were similar, with ∼47 µM and ∼45 µM, respectively (444). With a similar approach, a cytosolic PV concentration of ∼12 µM was estimated for hippocampal dentate gyrus basket cells (hDG BCs) (267). The PV concentration was variable among individual hDG BCs but similar in somata and boutons of a given cell. For cerebellar basket cell (cBC) somata, a substantially higher mean PV concentration of ∼565 µM was reported, which was also less variable among individual cBCs (267).

In other studies, the concentrations of CBPs were estimated from densities of gold particles with electron microscopic postembedding immunogold procedures. Quantification of particle densities allows for a statistical comparison of the relative levels of CBPs in somata, dendrites, dendritic spines, axons, and axon terminals. Using this approach, Kosaka et al. (477) found significantly higher levels of PV immunoreactivity in axons and axon terminals of Purkinje cells and basket cells than in their respective somata and dendrites. In contrast, CB-D28k immunoreactivity was more similar in somata, dendrites, and spines of Purkinje cells. Estimates for absolute PV concentrations were obtained by comparison to calibration curves deduced from quantitative immunogold analyses of standard PV samples. Estimated PV concentrations were 50–100 µM for Purkinje cell somata and dendrites as well as for interneuron somata and 1 mM or more in axons and axon terminals of Purkinje cells and cBCs (477), corresponding to 100–200 µM and 2 mM Ca^2+^ binding sites, respectively. Estimates for PV and CB-D28k concentrations in Purkinje cell somata were obtained more recently from calibrated immunogold tissue counts, with 116 µM and 208 µM for PV and CB-D28k, respectively (474), corresponding to 232 µM and 832 µM Ca^2+^ binding sites. In postnatal day 26 rats with fully developed hearing, cochlear inner hair cells contained one-tenth of the amount of CBPs of outer hair cells. In these latter cells, the cell body contained β-PV and CB-D28k at high levels equivalent to 5 mM Ca^2+^ binding sites. In contrast, the concentration of Ca^2+^ binding sites in inner hair cells was ∼0.5 mM and was dominated by α-PV (474).

**FIGURE 18.**
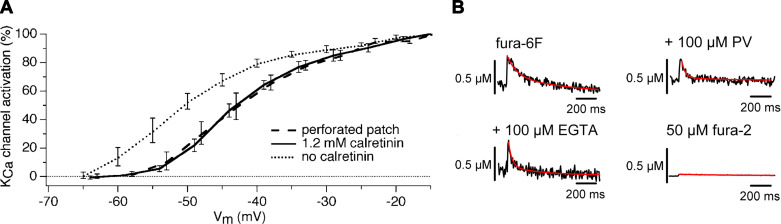
Estimating the concentration of endogenous buffers by functional rescue. *A*: exogenous calretinin (CR) in the pipette solution mimics the native Ca^2+^ buffer in suppressing the Ca^2+^-activated potassium channel (K_Ca_) current in frog saccular hair cells. K_Ca_ currents normalized to the amplitude at –15 mV are plotted against membrane potential (*V*_m_). The voltage dependence of K_Ca_ is similar to that seen in perforated-patch recordings if the pipette solution for whole cell recordings contains 1.2 mM CR. Modified from Ref. 478, with permission from *Nature Neuroscience*. *B*: exogenous parvalbumin (PV) in the pipette solution mimics the native Ca^2+^ buffer in accelerating the decay of action potential (AP)-triggered intracellular calcium concentration ([Ca^2+^]_i_) transients. Average waveforms of AP-evoked [Ca^2+^]_i_ transients during presynaptic whole cell recordings in calyx of Held terminals obtained with pipette solutions supplemented with either 100 µM fura-6F, 100 µM fura-6F + 100 µM recombinant PV, 100 µM fura-6F + 100 µM EGTA, or 50 µM fura-2. The slow Ca^2+^ buffer EGTA and recombinant PV restore the fast decay of AP-evoked [Ca^2+^]_i_ transients. Modified from Ref. 479, with permission from *Journal of Neuroscience*.

An alternative approach for estimating CBP concentrations is based on functional assays during which normal neuronal function in neurons lacking a certain CBP, either due to genetic ablation or because of washout during whole cell dialysis, is restored by supplying exogenous buffer via the patch pipette. With such a “rescue approach,” a concentration of 1.2 mM CR was estimated for frog saccular hair cells, corresponding to ∼6 mM Ca^2+^ binding sites. The criterion for rescue was the concentration of exogenous CR required to restore the voltage dependence of activation of Ca^2+^-sensitive potassium channels to the level of that measured in perforated-patch recordings (478) (FIGURE 18*A*). In voltage-clamped cochlear inner hair cells (IHCs) of young (postnatal day 14–23) constitutive triple KO (α-PV^−/−^CB-D28k^−/−^CR^−/−^) mice, a relationship between Ca^2+^ influx duration and exocytosis similar, but not identical, to that of wild type could be restored by buffer infusion via the patch pipette (223). The required mobile buffer concentrations were equivalent to ∼1 mM synthetic Ca^2+^ binding sites, half of them with kinetics as fast as BAPTA, the remainder with properties like EGTA.

Presynaptic AP-induced [Ca^2+^]_i_ transients in calyx of Held terminals decay with a biphasic time course when the intracellular medium is only minimally perturbed by brief dye preloading. When such transients are measured during standard whole cell recordings without added Ca^2+^ buffers, a slower monoexponential decay is observed. Ca^2+^ transient amplitudes differ only marginally between the two conditions. This finding is consistent with a washout of a mobile Ca^2+^ buffer with slow binding kinetics such as PV during whole cell recording. In agreement with this notion, AP-induced presynaptic [Ca^2+^]_i_ transients decayed slowly in calyx terminals of PV-deficient (PV^−/−^) mice. A fast decay of AP-evoked [Ca^2+^]_i_ transients was restored when whole cell recordings were performed with 50–100 µM of the slow buffers EGTA or PV added to the presynaptic pipette solution, indicating that unperturbed terminals probably contain an equivalent amount of PV (479) (FIGURE 18*B*).

### 9.4. Local [Ca^2+^]_i_ Domains Triggering Synaptic Vesicle Fusion

Local Ca^2+^ domains that are established when VGCCs open and rapidly collapse after channel closure dominate fast Ca^2+^ signaling. They may trigger synaptic vesicle fusion and also activate Ca^2+^-sensitive potassium channels, which contribute to AP repolarization.

Immobile endogenous Ca^2+^ buffers retard diffusion and thereby prolong the time until local domains of elevated [Ca^2+^]_i_ reach steady state. Mobile endogenous buffers, on the other hand, accelerate the redistribution of Ca^2+^ ions and thereby reduce both the amplitude of changes of local [Ca^2+^]_i_ and their spatial extent. The importance of the binding rate constant k_on_ as a major factor determining the effect of exogenous buffers on local Ca^2+^ domains was first considered in a study on Ca^2+^-sensitive BK potassium channels in adrenal chromaffin cells (480). It was observed that EGTA is much less effective than BAPTA in blocking the activation of these channels, even though the buffers have similar equilibrium dissociation constants *K*_d_ (TABLE 1) and consequently similar steady-state buffering power (see *Eqs. 33–35* and TABLE 4 for order-of-magnitude estimates of the spatial extent and kinetics of local Ca^2+^ domains). The size of the local domain depends mainly on the product of the binding rate constant k_on_ and the concentration of free, mobile chelator, [B]_0_.

Thus, anions that are present at high concentration in the cytosol may have a strong effect on local domains, even if their equilibrium buffering power is very small, e.g., because of a very fast off-rate constant. For example, gluconate^−^, which is often infused as the main anion in patch-clamp experiments together with ATP^2−^, was found to increase the Ca^2+^ influx necessary for eliciting a given amount of transmitter release by a factor of 2.7, when comparing experiments performed with different intracellular solutions containing either gluconate^−^ and ATP^2−^ or methanesulfonate^−^, lacking Ca^2+^ binding and no ATP^2−^ (60). Other small anions, present in unperturbed cells, as discussed in sect. 2.4.4, may have similar effects on local domains.

From the perspective of intracellular Ca^2+^ sensors, the terms “nanodomain” and “microdomain” are frequently used to describe the tightness of coupling between sites of Ca^2+^ entry and the sensor. However, these terms are not precisely defined with respect to spatial distances, and the distinction is primarily based on the experimentally determined effectiveness of the slow and fast buffers EGTA and BAPTA, respectively, in uncoupling the Ca^2+^-triggered process under study from Ca^2+^ influx. Synapses in which transmitter release is sensitive to EGTA (indicative of microdomain coupling) are the calyx of Held (481) and synapses between layer 5 cortical pyramidal neurons (482) in young rats. Synapses in which release is largely insensitive to EGTA (nanodomain coupling) are the mature calyx of Held (483), inner hair cells of the cochlea (484), GABAergic hippocampal dentate gyrus BC→GC synapses (485), glutamatergic cerebellar GC→PC synapses (486), and rod bipolar cell→amacrine cell synapses of the retina (487). Numerical simulations suggest mean coupling distance of ∼100 nm and <30 nm for microdomain and nanodomain coupling, respectively (243, 445, 450, 485, 486, 488).

To base the determination of coupling distance between VGCCs and docked synaptic vesicles exclusively on the differential effectiveness of EGTA and BAPTA may be an oversimplification because processes upstream of synaptic vesicle fusion can also be Ca^2+^ dependent, for example the resupply of fusion-competent synaptic vesicles (489–492) or the balance between distinct priming states (492). Therefore, reduced synaptic strength after prolonged presynaptic whole cell dialysis with EGTA-containing pipette solution may result not only from uncoupling of the Ca^2+^ sensor for vesicle fusion but also from reduced availability of fusion-competent vesicles unless resting [Ca^2+^]_i_ is guaranteed to remain unchanged while changing the concentration of free chelator. This can be achieved by maintaining a fixed ratio of Ca^2+^-bound to free buffer in the recording pipettes while increasing total amount of buffer.

Relative contributions of the fast CBPs CB-D28k and CaM to buffering of AP-evoked [Ca^2+^]_i_ transients at presynaptic active zones were studied by numerical simulations (451). These predicted that each buffer contributes to the reduction of AP-evoked local [Ca^2+^]_i_ transients and resulting decrease of synaptic vesicle fusion probability. At an assumed VGCC-to-synaptic vesicle distance of 40 nm, CB-D28k caused ∼50% reduction of the synaptic vesicle fusion probability relative to control simulations without CB-D28k and CaM. CaM had a stronger inhibitory effect of ∼80% reduction, and addition of CB-D28k on top of CaM caused only a minor further reduction (∼85%). The reduction of AP-evoked fusion at synapses that contain both CB-D28k and CaM is mainly caused by fast Ca^2+^ binding to the N- and C-lobes of CaM. CB-D28k plays only secondary roles (451). At a resting [Ca^2+^]_i_ of 50 nM, >99.8% of CaM C-lobes are in the Ca^2+^-free apo-state.

The slow Ca^2+^ buffer PV has Ca^2+^ binding kinetics similar to EGTA. Because of its relatively high affinity for Mg^2+^, the majority of PV is Mg^2+^ bound and little PV is free at physiological cytosolic [Mg^2+^]_i_ (sect. 4.2). Competition with bound Mg^2+^ slows the binding of Ca^2+^ to PV in response to a cytosolic [Ca^2+^]_i_ increase. However, if the total cytosolic PV concentration is high, sufficient amounts of PV are not Mg^2+^ bound but free and can therefore act as a fast Ca^2+^ buffer. For example, for cerebellar BC→PC synapses, which contain on average >0.5 mM PV, 5% of the PV is free at rest, 73% is bound to Mg^2+^, and 22% is bound to Ca^2+^, when assuming resting concentrations of 40 nM free Ca^2+^ and 400 µM free Mg^2+^ (267). Thus, there is ∼30 µM of free PV available that can rapidly bind Ca^2+^ and affect peak local domain [Ca^2+^]_i_. When free PV is depleted in the local domain, it is replenished both by diffusion of free PV from the periphery and from a large reservoir of Mg^2+^-bound PV, which, however, has to shed Mg^2+^ before being able to bind Ca^2+^. The Mg^2+^ binding of PV, therefore, represents a mechanism for generating new buffer (called “metabuffering”) (267), albeit on a slower timescale. This distinction between fast buffering by free PV and slower buffering following dissociation of Mg^2+^ is identical to that discussed above for skeletal muscle (sect. 7.2).

### 9.5. Modulation of Short-Term Plasticity by Ca^2+^ Buffers

During repetitive activation, the strength of synapses can transiently increase or decrease, resulting in synaptic facilitation or depression. Facilitating and depressing mechanisms are likely to operate simultaneously at many synapses, and the balance between the two defines magnitude and time course of changes in synaptic strength during stimulus trains. Local and global [Ca^2+^]_i_ signaling plays a key role in the presynaptic mechanisms of short-term plasticity (STP). Ca^2+^ buffers can affect presynaptic STP in two ways: *1*) buffers shape amplitude and spatial profile of local Ca^2+^ domains triggering transmitter release, and *2*) buffers determine the time course of global [Ca^2+^]_i_ changes, which occur in nerve terminals after diffusional equilibration after AP firing. These signals, also called “residual [Ca^2+^]_i_ changes,” are crucially involved in regulating STP.

**FIGURE 19.**
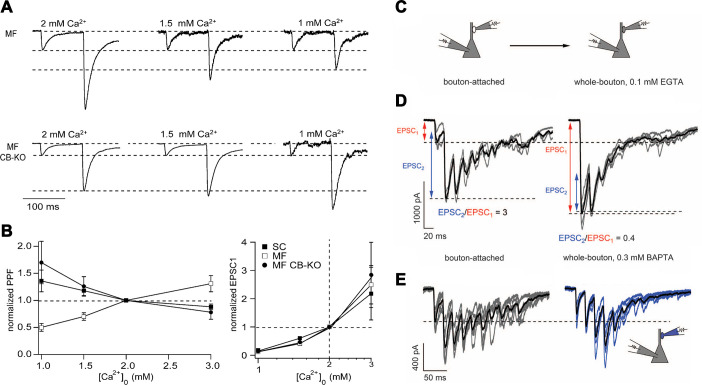
Ca^2+^ buffer saturation contributes to paired-pulse facilitation (PPF). *A* and *B*: opposite effects of changing Ca^2+^ influx on PPF in wild-type (MF, *top*) and CB-D28k-deficient (MF CB-KO, *bottom*) hippocampal MF→CA3 pyramidal cell synapses. *A*: excitatory postsynaptic currents (EPSCs) recorded in CA3 pyramidal cells during paired-pulse stimulation (interval 100 ms) of mossy fibers. *B*: paired-pulse ratios (EPSC_2_/EPSC_1_) normalized to those measured in 2 mM external calcium concentration ([Ca^2+^]_o_). In wild-type MF→CA3 synapses (□), increasing external [Ca^2+^] increases facilitation, whereas decreasing ([Ca^2+^]_o_ reduces facilitation. In CB-D28k-deficient MF→CA3 synapses (●), the opposite was observed. Normalized PPFs measured at Schaffer collateral (SC)→CA1 synapses (■) are shown for comparison. *Right* graph illustrates a similar dependence of EPSC_1_ on ([Ca^2+^]_o_ in all 3 synapses. Modified from Ref. 177, with permission from *Neuron*. *C*–*E*: a fast endogenous buffer attenuates transmitter release during the first action potential (AP). Buffer saturation during the first AP leads to higher local intracellular [Ca^2+^] ([Ca^2+^]_i_) during the second AP and thereby produces PPF at MF→CA3 synapses. Upon washout of endogenous buffer, substitution with the slow buffer EGTA alters PPF whereas the fast buffer BAPTA restores PPF. *C*: schematic illustration of recording configurations. Presynaptic APs were elicited via stimulation in bouton-attached (*left*) or whole bouton (*right*) configuration with either EGTA or BAPTA in the pipette. The whole bouton configuration leads to rapid washout of mobile endogenous buffers from the presynaptic MF terminal. *D*: EPSCs recorded in a CA3 neuron while the MF bouton was first stimulated in the bouton-attached configuration (*left*) and subsequently in the whole bouton configuration with 100 µM EGTA in the stimulation pipette (*right*). *E*: similar experiment as shown in *D* but with 300 µM BAPTA in the pipette. Modified from Ref. 494, with permission from *Science*.

The relationship between transmitter release rates and [Ca^2+^]_i_ is highly nonlinear, and the spatial profile of local [Ca^2+^]_i_ domains that build up in the vicinity of open presynaptic VGCCs after AP arrival is affected by Ca^2+^ buffers. Provided that these buffers bind Ca^2+^ fast enough, they are able to intercept incoming Ca^2+^ ions before binding to the Ca^2+^ sensor for transmitter release (see sect. 9.4). Thereby, Ca^2+^ buffers determine the [Ca^2+^]_i_ seen by the Ca^2+^ sensor and control synaptic vesicle fusion probability. During repetitive presynaptic AP firing, the concentration of free Ca^2+^ buffers may decrease if Ca^2+^ does not completely dissociate during interstimulus intervals or else if global [Ca^2+^]_i_ is transiently elevated. Such buffer saturation can lead to release facilitation because synaptic vesicles experience incrementally higher local [Ca^2+^]_i_ during repetitive stimulation (178, 493). Such a mechanism has been proposed to underlie facilitation at cortical synapses between multipolar bursting (MB) interneurons and pyramidal neurons (177) (FIGURE 19) and is analogous to the buffer saturation underpinning tetanic contraction in skeletal muscle (sect. 7.4). MB interneurons are CB-D28k positive, and MB→CA3 pyramidal cell synapses show pronounced paired-pulse facilitation (PPF). Washout of CB-D28k from MB interneurons during prolonged whole cell recordings increased the amplitude of the first responses and reduced PPF at MB→pyramidal cell synapses. Recordings in synapses of CB-D28k^−/−^ mice showed a similar pattern. CB-D28k loading into MB interneurons of CB-D28k^−/−^ mice via the recording pipette restored wild-type-like synaptic amplitudes and PPF (177). Taken together, these observations demonstrate that rapid Ca^2+^ binding to CB-D28k is able to reduce [Ca^2+^]_i_ within local domains that trigger vesicle fusion, and thus reduces synaptic strength.

Buffer saturation also contributes to PPF and frequency facilitation at hippocampal mossy fiber (hMF)→CA3 pyramidal cell synapses (177, 494). Synaptic facilitation counteracts a potential reduction in synaptic strength caused by the consumption of synaptic vesicles during repetitive AP firing. In contrast to many other types of synapses, which show increased synaptic facilitation when lowering external [Ca^2+^] to attenuate release probability and thereby prevent synaptic vesicle exhaustion, the magnitudes of both PPF and frequency facilitation are decreased in these synapses at lower external [Ca^2+^] (177). This finding is consistent with reduced buffer saturation due to reduced presynaptic Ca^2+^ influx (FIGURE 19, *A* AND *B*). Dialysis of hippocampal mossy fiber boutons (hMFBs) with pipette solution containing 0.1 mM EGTA strongly augmented initial excitatory postsynaptic currents (EPSCs) and decreased paired-pulse ratios, whereas dialyzing 0.3 mM BAPTA into hMFBs restored amplitudes and facilitation to a pattern similar to synaptic responses generated by stimulating unperturbed hMBs (494), consistent with the loss of a mobile fast binding endogenous Ca^2+^ buffer during whole cell dialysis of the hMFB terminal (FIGURE 19, *D* AND *E*).

**FIGURE 20.**
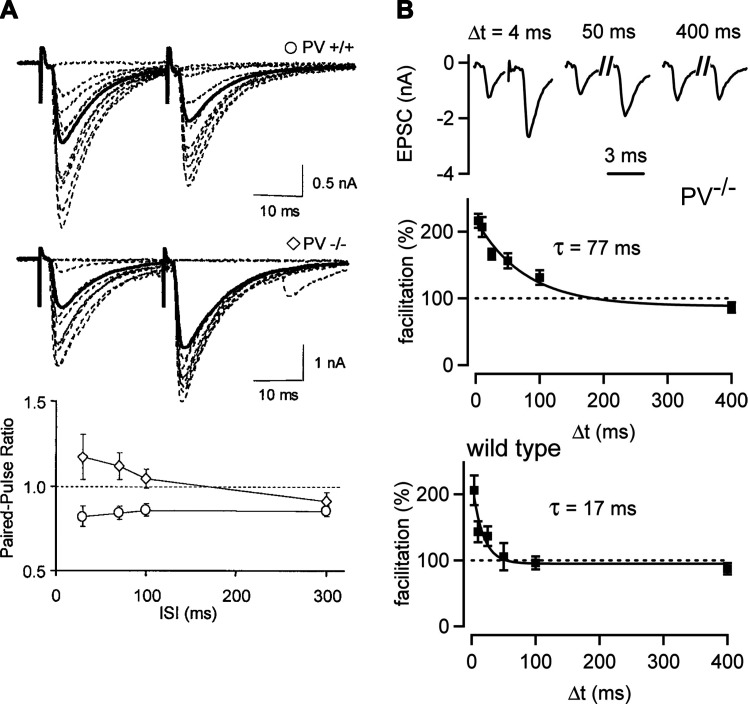
The slow endogenous buffer parvalbumin (PV) affects the time course of presynaptic global intracellular calcium concentration ([Ca^2+^]_i_) transients and thereby regulates synaptic short-term plasticity. *A*: absence of paired-pulse depression at GABAergic synapses between interneurons and Purkinje cells (PCs) of PV knockout (KO) mice. Inhibitory postsynaptic currents (IPSCs) in response to extracellular stimulations of GABAergic interneurons with paired stimuli (30-ms interval) were recorded in Purkinje cells of a PV^+/+^ mouse (*top*) and a PV^−/−^ mouse (*middle*). *Bottom*: average paired-pulse ratios [PPR = excitatory postsynaptic current (EPSC)_2_/EPSC_1_] are shown as a function of interstimulus interval for PV^+/+^ (circles) and PV^−/−^ (diamonds) mice. Modified from Ref. 495, with permission from *Proceedings of the National Academy of Sciences USA*. *B*: slower decay of PPF at glutamatergic calyx of Held synapses of PV^−/−^ mice. EPSCs were recorded in response to pulse pairs at 3 different interstimulus intervals (Δ*t*) in a PV^−/−^ calyx of Held synapse at reduced (0.6 mM) external [Ca^2+^] (*top*). PPRs plotted as a function of Δ*t* were fitted by an exponential function with a time constant of 77 ms (*middle*). Paired-pulse facilitation in a calyx synapse from a wild-type (PV^+/+^) mouse decayed notably faster, with a time constant of 17 ms (*bottom*). Modified from Ref. 479, with permission from *Journal of Neuroscience*.

GABAergic synapses between cerebellar interneurons and Purkinje cells (PCs) show paired-pulse depression (PPD) in response to paired stimuli delivered at intervals between 30 and 300 ms (495). In PV^−/−^ mice, in synapses as well as in whole cell recordings of connected interneuron-PC pairs, the same stimulus pattern induced PPF (267, 495). Wild-type-like short-term plasticity could be restored by loading recombinant PV into presynaptic interneurons (267). Likewise, dialysis of presynaptic interneurons with 1 mM of the slow Ca^2+^ buffer EGTA rescued PPD in synapses of PV^−/−^ mice. EGTA and PV do not reduce markedly the peak of AP-induced [Ca^2+^]_i_ transients, but both accelerate their initial rate of decay. Thus, the acceleration of the Ca^2+^ decay is likely to reduce the residual [Ca^2+^]_i_ and hence to attenuate facilitation (495) (FIGURE 20*A*).

Similar observations were made at calyx of Held synapses, which show pronounced PPF at low-release probability conditions (0.6 mM external [Ca^2+^]). PPF decays with a time constant of 17 ms in synapses of wild-type mice, whereas genetic ablation of PV expression increases the time constant of decay to 77 ms. PV^+/+^ and PV^−/−^ calyx of Held synapses show a similar magnitude of PPF when probed at an interstimulus interval of only 4 ms. However, at an interstimulus interval of 50 ms, PPF had nearly completely decayed in the wild type, whereas it was reduced to only half in PV^−/−^ calyx synapses (479) (FIGURE 20*B*). These observations are consistent with the notion that the slow Ca^2+^ buffer PV only marginally reduces the peak of residual [Ca^2+^]_i_ transients, because of its slow action. Nevertheless, PV binds Ca^2+^ fast enough to strongly accelerate their initial decay. In contrast to the case of calyx of Held synapses, genetic elimination of PV has only minimal effects on synaptic transmission at hippocampal BC→GC synapses, consistent with its low concentration in hBCs (267).

Cerebellar Purkinje cells (PCs), which express large amounts of CB-D28k and PV, are reciprocally connected via inhibitory synapses that show PPF during high-frequency activation. Surprisingly, PPF is not affected by the absence of either CB-D28k or PV at these GABAergic recurrent PC→PC synapses. Likewise, PPF measured in experiments on wild-type pairs of connected PCs that were dialyzed for 60–70 min with 10 mM EGTA remains unaltered compared with controls. Thus, PPF at PC→PC synapses is largely independent of the major endogenous Ca^2+^ buffers CB-D28k and PV and of the decay of residual [Ca^2+^]_i_. Instead, it was proposed that PPF results from long-lived Ca^2+^-bound states of the sensor for transmitter release (496).

### 9.6. Modulation of Delayed Asynchronous Transmitter Release by Ca^2+^ Buffers

In many synapses, two kinetically distinct components of AP-evoked transmitter release can be distinguished: *1*) synchronous release that is temporally tightly coupled to the arrival of the presynaptic AP and 2) delayed asynchronous release that can last up to hundreds of milliseconds (497, 498). The relative contributions of these components to the total release depend on synapse type and can change with repetitive synapse activation during which asynchronous release typically builds up with increasing frequency and duration of stimulus trains.

At synapses between interneurons of the cerebellar molecular layer (MLIs), the time course of global [Ca^2+^]_i_ determines the time course of delayed asynchronous release observed after stimulus trains (250). After 50-Hz stimulation, a barrage of asynchronous release events was observed that can last for up to ∼2.5 s after cessation of stimulation. This delayed release was strongly attenuated in PV^−/−^ mice. This is consistent with the postulated effect of the slow buffer PV on global [Ca^2+^]_i_ transients. In the presence of PV, the initial [Ca^2+^]_i_ decay is sped up because of Ca^2+^ binding. The subsequent unloading of Ca^2+^ from PV during the decline of global [Ca^2+^]_i_ generates a slowly decaying component of the [Ca^2+^]_i_ transient that supports delayed asynchronous release during a time window between 0.5 and 2.5 s after cessation of stimulation. In PV^−/−^ mice, the slow [Ca^2+^]_i_ transient component is absent, and delayed asynchronous release is strongly reduced.

A similar scenario was described for hippocampal mossy fiber to CA3 pyramidal cell (hMF→CA3) synapses, where brief 20-Hz EPSC trains are followed by delayed asynchronous release lasting for ∼1–2 s (499). [Ca^2+^]_i_ measurements in mossy fiber boutons revealed a slowly decaying time course of presynaptic global Ca^2+^. Treatment of the synapses with the membrane-permeable slow buffer EGTA-AM decreased the amplitude of delayed asynchronous release but prolonged its duration, reminiscent of what was observed in cerebellar MLI→MLI synapses of PV-expressing in comparison to PV-lacking mice (250).

Presynaptic terminals of glutamatergic endbulb→bushy cell synapses in the mammalian antero-ventral cochlear nucleus invariably express PV, but only a subset coexpress CR. Interestingly, postsynaptic neurons contacted by CR/PV-coexpressing terminals show lower rates of asynchronous release compared with those that are contacted by terminals void of presynaptic CR immunoreactivity (464). At glutamatergic calyx of Held→MNTB synapses, the magnitude of delayed asynchronous release observed after high-frequency stimulus trains strongly decreases during postnatal maturation. Developmental decrease in asynchronous release coincides with an upregulation of presynaptic CR expression, but virtually all calyces are PV immunoreactive already shortly after hearing onset (postnatal day 12) (463). Neither endbulb nor calyx terminals express CB-D28k. Whether this developmental downregulation of asynchronous release is related to differences in CR expression remains to be established. Nevertheless, these findings can be understood on the basis that CR is a fast buffer, which reduces the peak of the [Ca^2+^]_i_ transient. During the [Ca^2+^]_i_ decay, free and Ca^2+^-bound forms of CR are at equilibrium, such that *Eqs. 30–32* hold. Although these equations predict that the area under the [Ca^2+^]_i_ transient is unchanged in the presence of the buffer (FIGURE 7*D*), its amplitude is reduced (FIGURE 7, *A* AND C). For a cooperative process, such as Ca^2+^-triggered synaptic vesicle fusion, this implies a reduction of the overall effect of the [Ca^2+^]_i_ transient.

Among hippocampal dentate gyrus inhibitory interneuron→principal neuron synapses, PV-expressing interneurons provide temporally precise inhibition, which is tightly synchronized to the timing of presynaptic AP firing, whereas cholecystokinin (CCK)-expressing interneurons release GABA in a less synchronized manner and exhibit prominent asynchronous release for a few hundreds of milliseconds after stimulus trains. CCK-expressing interneurons seem to lack expression of PV, CB-D28k, and CR. It is therefore conceivable that the differential expression of PV contributes to the mechanisms responsible for the ∼15-fold difference in the ratio of synchronous versus asynchronous release at these two synapses by accelerating the initial decay of AP-induced [Ca^2+^]_i_ transients in PV-expressing interneurons and thereby reducing the number of asynchronously released quanta immediately following presynaptic APs (500).

### 9.7. Modulation of Excitability, AP Firing, and Network Activity

Neuronal membrane excitability and discharge properties are determined by an interplay of various voltage- and/or Ca^2+^-sensitive ion conductances. The amount of Ca^2+^ entering per AP is controlled by the AP duration, which needs to be precisely regulated to limit Ca^2+^ influx especially during repetitive AP firing. Many cortical neurons, including hippocampal granule cells (hGCs), express large-conductance Ca^2+^-sensitive potassium channels (BK channels), which contribute to AP termination. When recorded with pipettes containing 10 mM BAPTA, AP durations in hGCs increase similarly as observed after application of BK channel blockers, indicating that BAPTA uncouples BK channels from Ca^2+^ influx. Intracellular application of 10 mM EGTA does not affect AP kinetics, because BK channels are spatially tightly coupled to VGCCs. By quantitatively analyzing the extent of AP prolongation by different intracellular BAPTA concentrations, the mean diffusional distance for Ca^2+^ ions from VGCCs to BK channels was estimated to be ∼13 nm in hGCs (501). Such tight colocalization of the two types of channels together with low Ca^2+^ sensitivity of BK channels confines the activation of BK channels to short, well-timed episodes, which is required for fast AP repolarization. It also decouples channel activation from changes in global [Ca^2+^]_i_ levels.

CR is the main cytosolic Ca^2+^ buffer in cerebellar granule cells (cGCs), which provide the major excitatory input to Purkinje cells via their parallel fibers. Recordings in cGCs of CR^−/−^ mice showed that CR-deficient cGCs exhibit briefer APs compared with cGCs of wild-type mice. They also generate repetitive spike discharge with a steeper rise in firing frequency with increasing current injections. Addition of 0.15 mM BAPTA to the patch pipette solution restored normal excitability levels in CR-deficient cGCs, indicating that 0.15 mM BAPTA effectively mimics the contribution of CR to the cytosolic Ca^2+^-buffering power in cGCs of wild-type mice. A minimal single compartment-based model of cGC AP firing, which considers voltage- and Ca^2+^-sensitive conductances as well as Ca^2+^ buffering and extrusion, supports the conclusion that larger and faster-decaying AP-induced [Ca^2+^]_i_ transients in CR-deficient cGCs are a consequence of reduced Ca^2+^ buffering, which enhances activation of Ca^2+^-sensitive BK potassium channels, speeds up AP repolarization, and thus produces shorter APs that enable faster discharge rates (502).

A similar modeling approach was used to study the role of the slow buffer PV for regulating the discharge properties of striatal fast-spiking (FS) interneurons (503). A conductance-based model that also includes Ca^2+^-sensitive small-conductance (SK) potassium channels and the presence of a Ca^2+^ buffer reproduces average firing frequencies and spike adaptation as observed during whole cell recordings in striatal FS interneurons in response to current injection. Higher concentrations of PV lead to elevated [Ca^2+^]_i_ between consecutive AP-induced [Ca^2+^]_i_ transients when Ca^2+^ dissociates from PV and thereby facilitate activation of the SK current. This increases the duration of the afterhyperpolarization (AHP) following each AP and thereby delays the next AP and reduces the firing frequency. Thus, variable concentrations of PV in the cytoplasm of striatal FS interneurons can modulate their intrinsic excitability and may potentially alter striatal information processing (503).

Neurons of the reticular thalamic nucleus (RTN) express high levels of PV and are characterized by low-threshold voltage-activated (LVA) Ca^2+^ currents. Based on the firing patterns observed in extracellular in vivo recordings, four types of neurons can be distinguished in the RTN: irregularly firing, medium bursting, long bursting, and tonically firing. Neurons of the medium-bursting type are more frequently observed than those of the long-bursting type in PV^−/−^ mice. The generation of AP bursts involves Ca^2+^ influx thorough LVA VGCCs that subsequently activates Ca^2+^-dependent SK potassium channels. It is possible that a lack of PV in RTN neurons alters SK channel activation following Ca^2+^ influx and thereby affects their firing properties (504).

Cerebellar Purkinje cells generate two types of membrane discharges: simple and complex spikes. Simple spikes occur spontaneously or are triggered synaptically by parallel fiber input, whereas complex spikes are driven by climbing fiber input. Extracellular recordings in Purkinje cells from cerebella of adult calretinin-deficient (CR^−/−^) mice revealed a strongly enhanced spontaneous simple spike firing rate compared with wild-type mice, whereas mean spontaneous firing rates of complex spikes were unaltered. The duration of complex spikes was reduced in CR^−/−^ PCs, as was the duration of pauses in simple spike firing following spontaneous complex spikes (505). A reduced complex spike duration may decrease Ca^2+^ influx and thence activation of Ca^2+^-sensitive potassium channels, leading to shorter pauses in simple spike firing that normally follows the complex spike (505). Motor coordination is impaired in CR^−/−^ mice consistent with the role of the cerebellum in motor control and motor learning (506). Genetic ablation of both CR and CB-D28k (CR^−/−^CB-D28k ^−/−^) induces 160-Hz local field potential oscillations in the cerebellar cortex of alert mice, with PCs firing simple spikes phase-locked to the oscillations. Since the intrinsic excitability of PCs is unaltered in CR^−/−^CB-D28k ^−/−^ mice but oscillations reversibly disappear when gap junctions or either GABA_A_ or NMDA receptors are blocked, these 160-Hz oscillations are likely to emerge at the network level, demonstrating that changes in intracellular Ca^2+^ buffering in specific neuron types can alter network dynamics (507).

### 9.8. Modulation of Ca^2+^ Signaling in Dendritic Shafts and Spines

**FIGURE 21.**
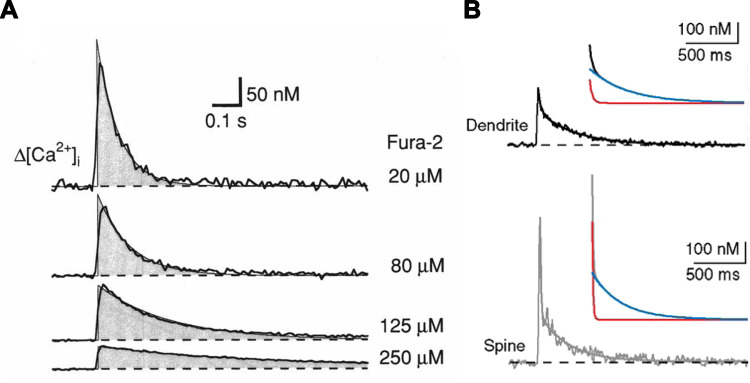
Endogenous Ca^2+^ buffers shape the time course of intracellular calcium concentration ([Ca^2+^]_i_) transients in dendrites and spines. *A*: [Ca^2+^]_i_ transients evoked by single action potentials (APs), measured in the proximal dendrites of cortical pyramidal neurons that lack expression of parvalbumin (PV), CB-D28k, and calretinin (CR) and therefore exhibit a low endogenous Ca^2+^-binding ratio, somatically loaded with either 20, 80, 125, or 250 µM fura-2. Gray areas represent the integral A × τ, the product of peak amplitude A and time constant τ fitted to the decay, which is similar under all recording conditions. Modified from Ref. 441, with permission from *Biophysical Journal*. *B*: slow and biphasic decay kinetics of [Ca^2+^]_i_ transients in dendritic shafts (*top*) and spines (*bottom*) of cerebellar Purkinje cells. The superimposed continuous lines represent double-exponential fits to the decay. The dashed lines represent an assumed resting [Ca^2+^]_i_ of 45 nM. *Insets* show the fits with their corresponding fast (red) and slow (blue) components. In the spine, the fit yielded a similar slow component but a much larger fast component, likely reflecting fast efflux of Ca^2+^ from the spine into the dendrite. Modified from Ref. 510, with permission from *Journal of Physiology*.

Dendritic Ca^2+^ signaling and its modulation by Ca^2+^ buffers has been studied experimentally by dye loading into dendrites of neocortical and hippocampal pyramidal neurons, cerebellar Purkinje cells and various interneurons, as well as by numerical simulations using deterministic or stochastic approaches. Dendritic [Ca^2+^]_i_ transients in pyramidal neurons evoked by backpropagating APs have amplitudes of several hundreds of nanomolar and decay rapidly with time constants generally <100 ms at physiological temperature (441, 508, 509) (FIGURE 21*A*). Neocortical and hippocampal CA3 pyramidal neurons lack expression of PV, CR, and CB-D28K. Hippocampal CA1 pyramidal neurons are also void of PV and CR but contain ∼45 µM CB-D28K (444). For these neurons, estimates for the endogenous Ca^2+^ buffer power in dendrites ranged between 170 and 200, indicating that <1% of the total Ca^2+^ entering per AP remains free. During AP trains, [Ca^2+^]_i_ increases to a steady-state level that depends linearly on the firing frequency. Thus, the dendritic [Ca^2+^]_i_ level linearly encodes the frequency of APs in pyramidal neurons (441). Dendritic Ca^2+^ transients elicited by backpropagating APs in bitufted interneurons in layer 2/3 of the somatosensory cortex have a mean amplitude of ∼140 nM and decay with a slow time constant of ∼200 ms, consistent with their higher endogenous Ca^2+^ buffer power (∼285) compared with that of pyramidal neurons (511). [Ca^2+^]_i_ transients measured in dendrites of hippocampal dentate gyrus basket cells (hDG BCs) show similar kinetics, with a mean decay time constant of ∼200 ms, but have smaller amplitudes, with a mean of only ∼40 nM. The endogenous Ca^2+^ buffer power in hippocampal dentate gyrus BC dendrites is ∼200. In these neurons, Ca^2+^ buffering power was estimated both during Ca^2+^ indicator loading and under steady-state conditions up to 20 min after establishing whole cell configuration, when mobile buffers are expected to be largely washed out. The similarity of the buffering power estimates obtained under these two conditions suggests that the Ca^2+^ buffering power in proximal apical dendrites of BCs is primarily determined by buffers that are resistant to washout (512). Similarly to pyramidal cells, dendritic [Ca^2+^]_i_ transients in these two classes of cortical and hippocampal interneurons summate linearly during short AP bursts (511, 512) whereas dendrites of midbrain dopamine neurons of the substantia nigra exhibit a supralinear summation of single AP-evoked Ca^2+^ transients (513).

AP-induced [Ca^2+^]_i_ transients in dendritic spines and shafts of Purkinje cells (PCs), which contain high concentrations of PV and CB-D28k, last considerably longer than those in pyramidal cell dendrites (510, 514) (FIGURE 21*B*). In both compartments, [Ca^2+^]_i_ transients decay double exponentially with fast and slow time constants of ∼20–30 and ∼300–400 ms, respectively (510). Whereas amplitudes of the slowly decaying component are comparable for [Ca^2+^]_i_ transients measured in dendritic shafts and spines, amplitudes of the fast-decaying components are considerably greater in spines, which largely accounts for the higher total amplitudes of their [Ca^2+^]_i_ transients. To reproduce the rapid initial decay of [Ca^2+^]_i_ transients in spines in numerical simulations, diffusional coupling had to be assumed, allowing free Ca^2+^ as well as all Ca^2+^-bound and free buffer species to diffuse between both compartments. The simulations further suggest that *1*) neither CB-D28k nor PV saturates in spines or shafts during climbing fiber-evoked Ca^2+^ transients, *2*) k_on_ of CB’s medium-affinity binding is fast enough to reduce [Ca^2+^]_i_ transient peaks, and *3*) slow Ca^2+^ binding to PV and CB-D28k leads to biphasic decay of [Ca^2+^]_i_ transients in dendritic shafts (510).

In cerebellar Purkinje cells, the functional consequences of genetic removal of one or both mobile Ca^2+^ buffers PV and CB-D28k were studied. Kinetic analysis of [Ca^2+^]_i_ transients in PC dendrites of CB-D28k^−/−^ mice (515, 516) and in spines and dendritic shafts of PCs of PV^−/−^ and PV^−/−^CB-D28k^−/−^ mice (510) shows that Ca^2+^ buffers contribute to sculpting amplitude and decay of [Ca^2+^]_i_ transients. In CB-D28k^−/−^ PCs, resting [Ca^2+^]_i_ is unaltered but peak amplitudes of synaptically evoked dendritic [Ca^2+^]_i_ transients associated with complex spikes are enhanced on average by >80% compared with wild-type PCs. This is mainly due to an increase of the fast, but not the slow, decay component of [Ca^2+^]_i_ transients (515, 516). In PV^−/−^ PCs, [Ca^2+^]_i_ transients reach the same peak amplitudes as in wild-type animals, but the biphasic nature of the decay is less pronounced. When both PV and CB-D28k are removed (PV^−/−^CB-D28k^−/−^), peak amplitudes of [Ca^2+^]_i_ transients are about two times higher than those in wild-type PCs, and their decay is nearly monophasic (510).

### 9.9. Ca^2+^ Buffers in Neurological and Neurodegenerative Diseases

It was originally considered that Ca^2+^ binding proteins may exert a neuroprotective function by protecting cells against damaging effects of excessively high [Ca^2+^]_i_ during periods of strong activity (517). Distinct populations of neurons are selectively affected by different neurodegenerative diseases, which could be facilitated by a weakening of their Ca^2+^ buffering power because of intrinsically low or diminished expression of CBPs. Differential expression of CBPs in neuronal subpopulation could thus contribute to defining their susceptibility to disease processes. For example, motoneuron populations lost early in amyotrophic lateral sclerosis (ALS) lack CB-D28K and PV expression, whereas those damaged late or infrequently express higher levels of these CBPs (518). On the other hand, a substantial loss of neostriatal neurons containing CB-D28K has been reported for postmortem brain specimens from patients diagnosed with Huntington’s disease, indicating a selective vulnerability of these neuron populations (519). In general, the concept of CBPs primarily serving neuroprotective functions has received only limited experimental support. Rather, CBPs are essential components in neuronal Ca^2+^ homeostasis and signaling that primarily regulate the timing and spatial extent of Ca^2+^ signals. Deficiencies in one or more CBPs lead to distinct alterations in neuronal excitability and/or synaptic physiology that possibly also penetrate to the behavioral level.

Mice with genetic ablation of PV, CB-D28k, or CR expression are generally healthy and fertile and show no gross abnormalities in brain morphology (520). For example, CB-D28k^−/−^ mice develop normally and have a normal anatomy and synaptic connectivity with no signs of cell death. However, CB-D28k-deficient mice show motor deficits. They develop a graded cerebellar ataxia, which is correlated with marked changes in the amplitude and kinetics of synaptically mediated [Ca^2+^]_i_ transients, and display aberrant network activity (515). In CR^−/−^ mice, gross development and many morphological, biochemical, and behavioral characteristics were found to be unaffected, but these animals show a motor discoordination that worsens dramatically with age (505, 521). In addition, long-term potentiation (LTP) but not basal synaptic transmission is impaired in CR^−/−^ mice at synapses between the perforant pathway and granule cells in the dentate gyrus connecting entorhinal cortex with the hippocampal formation. Normal LTP can be restored in these animals by application of the GABA_A_ receptor antagonist bicuculline, suggesting that in CR^−/−^ dentate gyrus an excess of GABA release interferes with LTP induction. Thus, expression of CR contributes to the control of synaptic plasticity in mouse dentate gyrus by indirectly regulating the activity of GABAergic interneurons (522). Despite such roles of Ca^2+^ buffers in neuronal signaling, a complete absence of α-PV, CB-D28k, and CR has little impact on cochlear function and hearing when comparing distortion-product otoacoustic emissions and auditory brain stem responses between constitutive triple KO and wild-type mice (223). PV^−/−^ and PV^+/−^ mice show social behavior deficits with an autism spectrum disorder (ADS)-like phenotype including impairments in communication and repetitive and stereotyped patterns of behavior (523). This seems to relate to an absent or reduced PV expression in PV^−/−^ and PV^+/−^ mice, respectively, rather than to a loss of fast-spiking GABAergic interneurons (523).

We are unaware of human monogenic disorders causally linked to mutations in the CB-D28k, PV, or CR genes. However, altered expression of the CBPs PV, CB-D28k, or CR has been reported in the context of neurological diseases including ADS, dementia, epilepsy, and ataxia (524, 525). For example, a decrease in the levels of CB-D28K has been found in the cortex of brains of Alzheimer disease (AD) patients (526, 527). Vulnerability to AD also extends to PV-containing interneurons. A decrease in PV immunostaining was reported for parts of the entorhinal cortex when AD neuropathological markers are present. As the density of pathological markers in the entorhinal cortex becomes greater and more widespread, the decrease of PV immunostaining encompasses additional layers, even though some changes that are observed in PV-expressing interneurons may be linked to the fate of the projection neurons on which they synapse (528). AD disease-sensitive PV-containing inhibitory interneurons were further identified in the perirhinal cortex of AD patients (529). In the temporal cortex of AD patients the number of PV-immunoreactive somata was unchanged, but in layer II a decreased density of terminals of PV-expressing chandelier cells was observed, suggesting that not the interneurons themselves but only their terminals are decreased (530).

These findings raise the possibility that dysregulation of Ca^2+^ buffering power in certain neuron populations may contribute to the etiopathology. It is, however, challenging to differentiate between selective loss of neurons expressing a certain CBP and decreased protein expression. For example, for caldendrin, a member of the neuronal calcium-binding protein (nCaBP) family with rapid Ca^2+^-binding kinetics and high abundance in postsynaptic density of spine synapses, it was reported that in postmortem brains of subjects with chronic schizophrenia the number of caldendrin-immunoreactive neurons is significantly reduced in the left dorsolateral prefrontal cortex (531). Despite the reduced number of immunoreactive neurons, absolute caldendrin protein levels were elevated (532). A reduction of mRNAs encoding PV was found in the prefrontal cortex of subjects with schizophrenia. This was primarily due to a reduction in neuronal PV mRNA expression rather than a decreased density of PV mRNA-positive neurons. In contrast, the same measures of CR mRNA expression were not altered in schizophrenia (533).

## 10. EPITHELIA

Transport of Ca^2+^ across epithelia is important for the body’s Ca^2+^ homeostasis (for review see Ref. 534). To summarize, Ca^2+^ is absorbed by the intestine and then excreted from the body via the kidneys. Although the kidney may be a site of net Ca^2+^ loss from the body, it is important to remember that there is a balance between glomerular filtration and subsequent reabsorption, so Ca^2+^ (re)absorption occurs in both kidney and intestine. There are two routes for transepithelial Ca^2+^ transport. Some can occur via a paracellular route, through tight junctions between cells (535). Another important route involves transcellular transport with Ca^2+^ present in the lumen, entering the cell, along its electrochemical gradient, before being actively transported (by NCX and/or PMCA) at the basolateral membrane into the extracellular fluid and thence the blood.

Early work on rat duodenum found that calcium absorption comprised two components as characterized by their response to elevating luminal [Ca^2+^] and vitamin D. One was a saturating function of luminal [Ca^2+^] and was stimulated by vitamin D, whereas the other was proportional to [Ca^2+^] and independent of vitamin D (536, 537). The transport through the former pathway was found to be proportional to the concentration of a vitamin D-induced CBP (536, 538, 539). This CBP was subsequently shown to be comprised of the two calbindins (CBs) CB-D9k and CB-D28k (for review see Ref. 540). CB-D9k has two Ca^2+^ binding sites, and CB-D28k has four (30, 186) (see TABLES 2 and 3). In general, tissues only express one of the two CBs, for example CB-D9k in the duodenum and CB-D28k in the kidney.

A major challenge for the transport mechanism described above is that, to move between the apical and basolateral membranes, Ca^2+^ must traverse the cytoplasm, where [Ca^2+^]_i_ is very low. It was calculated that the diffusion rate of free Ca^2+^ would be ∼70 times smaller than the experimentally measured transepithelial flux (541). Therefore the role of CBs is to allow rapid diffusion of Ca^2+^ across the cytoplasm while maintaining [Ca^2+^]_i_ low. As pointed out previously (174, 200, 209) (see also sect. 4.5), there are two opposing factors. *1*) Since CB has a larger MW than Ca^2+^, the diffusion rate of an individual Ca^2+^ bound to CB will be much slower, in proportion to the 0.33th power of the ratio of molecular weights, than that of a free Ca^2+^ ion. Calcium has an atomic weight of 40, so Ca^2+^ bound to CB-D9k will diffuse at a speed of about (40/9000)^0.33^ ≈ 16% of that of free Ca^2+^. For Ca^2+^ bound to CB-D28k, the corresponding value is ∼12%. This effect is greatly outweighed by the fact that the concentration of Ca^2+^ bound to CB is much higher than that of free Ca^2+^ so the net effect is a marked increase in the rate of Ca^2+^ diffusion (≈50-fold with the values of TABLE 3). It should also be noted that free Ca^2+^ ions will bind to fixed charges in the cell, slowing their diffusion. Charge neutralization by binding to CB will thereby accelerate diffusion. We could not find measurements of the contribution of fixed buffers in epithelia, but, given the high concentration of CBs, it is probable that Ca^2+^-transporting epithelia represent a case where the majority of buffering comes from mobile rather than fixed buffers.

Evidence suggests that CB-D9k plays the major role as the CBP in the intestine. A similar mechanism involving CB is also important for renal reabsorption of Ca^2+^ in the distal convoluted tubule (DCT), but here CB-D28k is the major player. Indeed, the appearance of CB in urine has been proposed as a biomarker for damage to the distal nephron (542). PV is also expressed in the cells of the DCT (543). Knockout of PV resulted in diuresis. PV also appeared to act as a Ca^2+^ buffer and thereby reduced the amplitude of intracellular Ca^2+^ signals, suggesting that the effects of PV are as an intracellular Ca^2+^ buffer modulating Ca^2+^ signaling as opposed to transport (544). It has also been shown that most of the PV in the kidney is located in the early DCT, a region where there is little Ca^2+^ transport (543, 545). In other sections we have discussed the importance of the kinetics of PV in shaping Ca^2+^ signals. Given the slow kinetics of changes of [Ca^2+^]_i_ in the DCT (544), it is unclear whether these kinetics are relevant. It is, of course, possible that fast changes of [Ca^2+^]_i_ in a subcompartment of the cytoplasm are affected by PV.

Work showing that CB-D9k is localized to the basolateral membrane in the rat distal nephron has also led to the suggestion that it may regulate Ca^2+^ transport as well as buffering (543). The entry of Ca^2+^ along its electrochemical gradient from lumen into cell occurs in many epithelia through TRPV5 channels, with the Ca^2+^ subsequently binding to CB and being transported across the cell. It has been shown that the expressions of CB and TRPV5 are coordinated (546). As well as being present in the cytoplasm, a fraction of the CB is localized on the apical membrane, and this apical fraction is decreased in TRPV5 knockout mice. It has been suggested that Ca^2+^ buffering by CB in the vicinity of the TRPV5 channel increases influx through this channel.

Mg^2+^ is also reabsorbed by the DCT, and it has been suggested that it enters the cell from the lumen via a TRPM6/7 channel (547). That work showed that the TRPM channel was located close to either PV or CB-D28k, and it was suggested that binding of Mg^2+^ to buffers may be important in transepithelial Mg^2+^ transport (548). As pointed out in sect. 2.2, many Ca^2+^ buffers can also bind Mg^2+^. The major Mg^2+^ buffer is probably ATP. The relative molecular weights (atomic mass of Mg^2+^ is 24 and MW of ATP is 500) mean that the bound form diffuses at (24/500)^0.33^ = 0.37 of the free. Given a free Mg^2+^ of 1 mM, a total ATP concentration of 5 mM and *K*_d_ of 100 µM for Mg^2+^ binding to ATP, we obtain from *Eq. 27* that the flux of bound will be only 12% of that at the free. The contribution to Mg^2+^ diffusion of protein buffers, with higher molecular weights and lower concentrations than ATP, will be even smaller. Consistent with this, knockout of CB-D28k does not affect Mg^2+^ balance (549).

## 11. IMMUNE CELLS

Early studies using the Ca^2+^ indicator quin-2 found that the intrinsic buffering in human neutrophils could be represented by ∼0.76 mM of a buffer with *K*_d_ of 0.55 µM (550), equivalent to a buffer power of ∼1,000 at low [Ca^2+^]_i_. Neutrophils and monocytes were then shown to contain CBPs of the S100 family (551). The major components are migration inhibitory factor-related proteins 8 and 14 (MRP8 and MRP14), more commonly known, respectively, as S100A8 and S100A9, which form a dimer known as calprotectin. Two such dimers can form a tetramer, particularly at elevated [Ca^2+^], and this tetramerization is essential for biological function (552). Calprotectin can also bind Mn^2+^ and Zn^2+^, and it has been shown that release of calprotectin into the extracellular fluid has an antibacterial action by chelating these ions (553–555). Mn^2+^ is required for the function of bacterial superoxide dismutase as well as a variety of other bacterial enzymes, and Zn^2+^ is required for several bacterial enzymes (556). It appears that Ca^2+^ binding to the EF hands on calprotectin increases the affinity for Zn^2+^. Consequently, the affinity for Zn^2+^ and Mn^2+^ will be much greater outside, where Ca^2+^ is higher, than inside the cell, thereby allowing calprotectin to avidly bind Mn^2+^ or Zn^2+^ once it is released into the extracellular fluid where the bacteria are present (557, 558). The sequestration of extracellular Mn^2+^ and Zn^2+^ will therefore deprive bacteria of these essential metal ions, thereby preventing their growth (556).

Activated macrophages have been shown to release β-PV (oncomodulin), and this stimulates the outgrowth of axons from retinal ganglion cells (559). This effect could not be mimicked by other CBPs, suggesting that it results from an action of oncomodulin other than Ca^2+^ binding. Subsequent studies have also shown that oncomodulin released from neutrophils has similar effects (560).

A very different role of Ca^2+^ buffers has been suggested as a defense against West Nile virus. Infection of a cell by this virus has been shown to increase [Ca^2+^]_i_ (561). Subsequent work found that CB-D28k decreased viral replication in cultured cells (562). A study with another virus (Borna disease virus) found that infection increased expression of CB-D28k in submucous and myenteric neurons (563). Although the effects of CB on viral invasion may be due to effects on [Ca^2+^]_i_, direct measurements are required to confirm this.

## 12. CONCLUSIONS

The physiological properties of Ca^2+^ buffers have been studied in a variety of tissues. Their biochemical parameters such as ion specificity, binding kinetics, affinity, and cooperativity of binding generate a range of properties required for optimal cell function. Endogenous and exogenous Ca^2+^ buffers shape local and global [Ca^2+^]_i_ transients in predictable ways in different cell compartments. Together with Ca^2+^ influx and extrusion, the specific properties of Ca^2+^-binding molecules enable cells to encode activity using the universal second messenger Ca^2+^ and relay signals to specific intracellular pathways. By shaping and confining local [Ca^2+^]_i_ domains, Ca^2+^ buffering allows local signaling at spatially separate sites to coexist with global Ca^2+^ actions.

Some Ca^2+^-binding proteins serve a dual role as detectors of Ca^2+^ signals and Ca^2+^ buffers, whereas others act mainly as buffers shaping the spatiotemporal properties of intracellular Ca^2+^ signals. Ca^2+^ buffers can sculpt intracellular Ca^2+^ signaling in many ways. They can accelerate or retard the diffusion of Ca^2+^ in the cell, sharpen the spatial profile of local Ca^2+^ domains that build up during transmembrane Ca^2+^ flux, and thereby contribute to isolating local from global Ca^2+^ signaling pathways.

Each Ca^2+^ buffer can be used in several cell types. For example, the calbindins facilitate both epithelial Ca^2+^ transport and neuronal signaling, and the slow kinetics of parvalbumin are used to selectively accelerate the decay of Ca^2+^ signals in both neurons and skeletal muscle without interfering with their rise and amplitude. There are, however, tissue-dependent differences in buffering. Cardiac muscle is at one extreme, with most of the buffering provided by the nondiffusible buffers TnC and SERCA, essential for excitation-contraction coupling, with only a small contribution from molecules whose major function is to buffer. Skeletal muscle is broadly similar but also makes use of the diffusible buffer parvalbumin. In contrast, in neurons a high cytosolic Ca^2+^ buffering power is often achieved by expressing high amounts of one or two species of mobile Ca^2+^ buffer proteins. In general, neurons with low Ca^2+^ buffering power exhibit relatively fast decays of global [Ca^2+^]_i_ transients, whereas those with high buffer power, as a consequence of expression of high amounts of Ca^2+^ binding proteins, exhibit slower or biphasic global [Ca^2+^]_i_ transients. In neurons, this type of multiphasic kinetics of [Ca^2+^]_i_ transients allows cells to shape responses to action potentials in cell-specific ways and thereby produce a variety of short-term plasticity patterns. Slow tails of [Ca^2+^]_i_ transients build up during repetitive activity and may support asynchronous transmitter release and enable various forms of synaptic plasticity at intermediate timescales. Understanding of the quantitative effects of buffering has advanced less for many other tissues than is the case in nerve and muscle, and more research is required. For now, we will have to be satisfied with knowing which buffers are present and applying the basic principles learned from work on excitable cells.

A major area for future work concerns the role of changes of Ca^2+^ buffering in disease. Although there is evidence for such a link in cardiac muscle, it remains to be seen what the situation is in other tissues.

## GRANTS

D.E. was funded by a British Heart Foundation Chair (CH/2000004/12801) and Grant FS/CRTF/21/24140. E.N.’s and H.T.’s work is supported by the German Research Foundation (DFG), Collaborative Research Center 1286 “Quantitative Synaptology.” E.N. acknowledges funding by the DFG under Germany’s Excellence Strategy-EXC 2067/1-390729940. G.S. was funded by British Heart Foundation Grant PG/19/55/34545.

## DISCLOSURES

No conflicts of interest, financial or otherwise, are declared by the authors.

## AUTHOR CONTRIBUTIONS

D.E., E.N., H.T., and G.S. conceived and designed research; D.E., H.T., and G.S. prepared figures; D.E., E.N., H.T., and G.S. drafted manuscript; D.E., E.N., H.T., and G.S. edited and revised manuscript; D.E., E.N., H.T., and G.S. approved final version of manuscript.
